# Fish species identification and composition across the Scotia Sea during austral summer 2019

**DOI:** 10.1038/s41597-025-05217-z

**Published:** 2025-05-30

**Authors:** Tor Knutsen, Merete Kvalsund, Rupert Wienerroither, Kjell Bakkeplass, Julio Erices, Nicolas Straube, Monica Bente Martinussen, Alejandro Mateos-Rivera, Alina Rey, Jon Rønning, Georg Skaret, Bjørn Arne Krafft

**Affiliations:** 1 Institute of Marine Research, Bergen, Norway; 2 Department of Natural History, University Museum of Bergen, Bergen, Norway

**Keywords:** Ichthyology, Ecology

## Abstract

An assessment of the fish species composition was conducted during the austral summer of 2019 in the Southwest Atlantic sector of the Southern Ocean (FAO Fishing Areas 48.1–48.4). Samples were collected from stations systematically spaced across the Scotia Sea region using a fine meshed pelagic trawl (“Macroplankton trawl”) towed through the ~200-0 m depth stratum (n = 127 hauls) and a coarser meshed and larger pelagic trawl (“Harstad trawl”) hauled from ~1000-0 m (n = 8 hauls), particularly suitable for catching somewhat larger sized specimens. In total, 56 fish taxa were identified from both traditional taxonomic visual and molecular identification methods. The dominating taxa, both in numbers and catch weight across the study area, were the two lanternfish species *Electrona antarctica* and *Gymnoscopelus braueri* (Myctophidae), together with the deep-sea smelt genus *Bathylagus* (Bathylagidae). The given description of taxonomic identification features including images of type specimens presented, constitutes a high-resolution and comprehensive encyclopedia that will facilitate future assessment of fish communities and interspecific relationships.

## Background & Summary

Populations in a geographic area are not static but change over time. Knowledge about species composition and distribution is fundamental for assessing the well-being of animal groups and can allow evaluations of how species’ populations are likely to change in the future. Habitats change due to several factors like climate change, resource exploitation and pollution. To a certain degree a species’ ecological plasticity will determine how its population dynamics and reproductive output will be affected. Monitoring of the global climate shows that the Scotia Sea and the waters near the Antarctic Peninsula in the Southern Ocean have experienced rapid warming during the second half of the twentieth century^1–3^. A commercial fishery for Antarctic krill *Euphausia superba* commenced in the 1970s and has been managed by the Commission for the Conservation of Antarctic Marine Living Resources (CCAMLR) since 1982 (www.ccamlr.org). This fishery has mainly been taking place in the Scotia Sea region during the last three decades, using midwater pelagic otter trawls, usually in the ~0–200 m depth stratum. Larval and juvenile fish are regularly found as bycatch in Antarctic krill fisheries; however, bycatch rates are comparatively lower than for other trawl fisheries globally^4^. Knowledge of vulnerable periods and areas when early life stages of fish are in abundance will be important for managing the spatial distribution of the fishery, particularly as it develops, but also if environmental conditions change^5–7^.

Assessments of fish species composition and distribution from the Scotia Sea and the waters near the Antarctic Peninsula in the Southern Ocean are scarce, often collected over small scales, hence data on species distribution across this region can be outdated. Moreover, small sized and often transparent early life stages of fish can be hard to detect visually (cf. Everson *et al*.^8^), but it can also make taxonomic identification difficult. There is both a lack of knowledge and adequate descriptions of key taxonomic characters of some species’ larval stages, and therefore identification keys need to be improved^9,10^.

The aim of this study was to assess the fish species bycatch composition and distribution at the lowest possible taxonomic level from sampling locations distributed over a large geographic area stretching from the Antarctic Peninsula to waters north of South Georgia during the austral summer of 2019. We provide data on geographical distribution, total weight per taxon and station, as well as length and sex (selected species only), for the 56 different fish taxa identified. Voucher specimens were deposited at the University Museum of Bergen (ZMUB), and through DNA barcoding mitochondrial Cytochrome c oxidase subunit I (COI) sequences were uploaded to GenBank. In both cases, accession numbers are given in the data repository. As supplementary information we also present photos annotated with taxonomic characters for all taxa (both adults and juveniles if present) as well as distribution maps and length histograms for the more abundant species found during the survey (Supplementary File 1). The annotated images will help develop more complete and updated field guides for identification of adults and larval fish in future studies. Even though we did not find all species previously reported from the covered region (e.g. Gon and Heemstra^11^), our data present a high number of species identified, which will fascilitate taxonomic identification of adults and early life stages of fish in future studies. The data are important as baseline for comparative studies and assessments and could be used also in predictions of future change, helping to understand potential impacts of environmental change and fishing activity.

## Methods

**Fig. 1:**
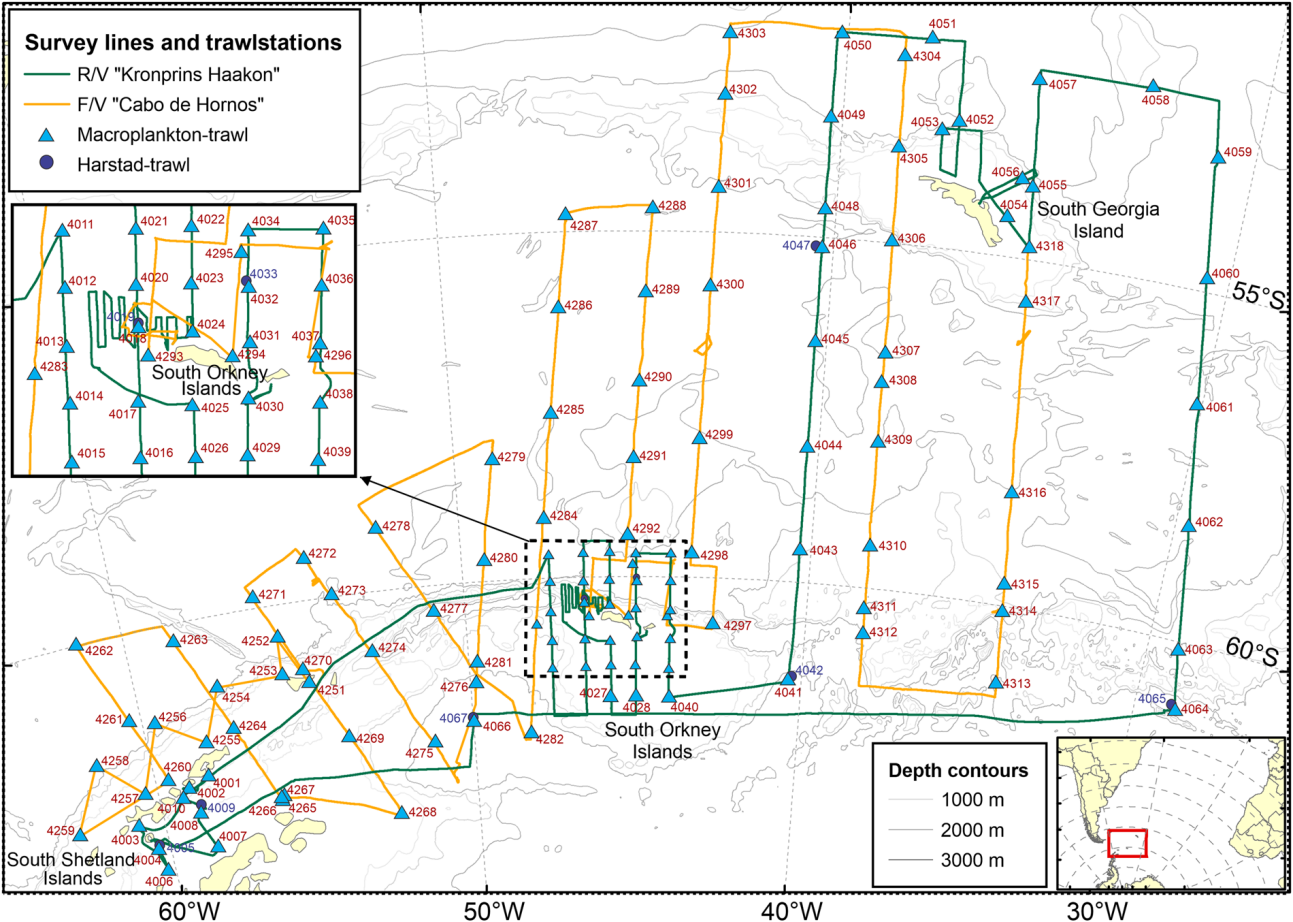
Fish and ichthyoplankton stations (N = 135) performed along the acoustic cruise lines by two vessels: RV Kronprins Haakon (N = 67 trawl hauls, 8 January – 24 February 2019) and FV Cabo de Hornos (N = 68 trawl hauls, 8 January – 3 March 2019). Blue circles: Harstad trawl ~1000-0 m. Cyan triangles: Macroplankton trawl ~200-0 m (cf. Table S1).

This study represents a two-vessel effort as part of a large-scale combined acoustic and trawl survey mainly designed to assess krill abundance in the Southwest Atlantic sector of the Southern Ocean and across the Scotia Sea during the 2018–19 austral summer^12^. Fish and ichthyoplankton were collected from a total of 135 trawl stations performed along acoustic transects by the two vessels: RV Kronprins Haakon (N = 67 trawl hauls, 8 January – 24 February 2019) and FV Cabo de Hornos (N = 68 trawl hauls, 8 January – 3 March 2019) (Fig. 1, Table S1).

A Macroplankton trawl was used on both vessels (N = 127 trawl hauls) with a nominal opening of ~36 m^2^ and a knot-to-knot diamond net mesh size of 3 mm (7 mm stretched) of the inner liner stretching from the trawl mouth to the cod-end^13,14^. A pelagic Harstad trawl, was used only on the RV Kronprins Haakon (N = 8 trawl hauls), with an opening of about 318 m^2^ and was equipped with a 22 mm meshed inner liner^15–17^. The trawls were deployed throughout the day at predetermined positions along the cruise lines. The trawls were deployed rapidly to max target depth, ~200 m (Macroplankton trawl) or ~1000 m (Harstad trawl), or shallower depending on bottom depth, and towed obliquely to the surface in a “single net oblique tow” (c.f. Wiebe *et al*.^18^). The trawls were presumed not to be fishing during deployment to the target depth. Vessel speed during trawling was between 1.5–3 knots. On RV Kronprins Haakon, trawl speed (Scanmar HC4-TSS) and trawl sounder (Scanmar HC4, SS4) sensors were attached to the trawl headline and recorded information about trawl depth, speed through water, and net-symmetry, together with trawl-door sensors provided information about tow duration, door-spread and overall trawl performance. On FV Cabo de Hornos, the Macroplankton trawl was towed using a 6 m wide steel beam with 200 kg weights at each lower wing tip to ensure best possible geometric stability of the trawl during sampling^14,19^. On both vessels, a Seabird SBE37SM CTD was mounted on the headline of the trawl to obtain additional data on the oceanography in the upper ~200 m of the water column. These data were also used to visualize the trawl trajectories and partly as support information when calculating volumes filtered for the Macroplankton trawl hauls. For both Seabird SBE37SM CTD’s the temperature and conductivity sensors were calibrated 20 October 2018, while the pressure sensor was calibrated 23 October 2018.

Onboard RV Kronprins Haakon the SCANMAR/SCANBASE system received navigation data from the vessel’s navigation system (GPS). These data in its raw form were stored in Scanmar custom built text files, as well as daily *.csv files, the latter each containing ~50–56 Mbyte holding Scanmar NMEA telegrams and GPS navigation telegrams for date, time (ZDA), and geographic position (latitude and longitude, GLL). The telegrams were recorded with a time resolution of approximately 3 seconds. These data could be coupled to the Scanmar depth sensor but could also be time synchronised to the CTD date, time and depth information obtained. From these files the vessel latitudes and longitudes when the trawl was at its maximum depth and when it surfaced and the trawling terminated, were retrieved and the horizontal distance between the two points calculated. Finally, the lengths of the oblique trawl paths applying Pythagoras’ theorem were computed. The latter, along with the normalized trawl opening of 36 m^2^ was used to calculate the trawl volumes filtered as a standardized way to present and compare abundance data (cf. Dornan *et al*.^20^). Another way of presenting standardized data and compare numbers, could be to use the weight or numbers per nautical mile trawled. A direct comparison of Macroplankton trawl catches with those of the Harstad trawl should not be undertaken, a) because the latter trawl was deployed to ~1000 m standard depth, while the Macroplankton trawl was typically deployed to ~200 m depth or less, and b) the Macroplankton trawl is a non-graded trawl while the Harstad trawl is a graded trawl having variable mesh size from the opening to the cod-end of the net.

On FV Cabo de Hornos there was no SCANMAR sensor to monitor trawl performance. However, navigation data were obtained from the ship’s GPS, and a custom-built cruise logger application ascertained that latitude and longitude along the cruise lines and at station, average vessel speed during trawling and the exact time for the trawl to reach maximum depth and to resurface were recorded. Maximum depth of trawling was derived from the CTD mounted on the headline of the trawl. Using the average vessel speed during trawling, the towed horizontal distances were determined from the point where the trawl was at its maximum depth to the point where it surfaced. Hence the length of the oblique trawl path was calculated applying Pythagoras’ theorem and finally the volume filtered for each haul was computed. Given that the FV Cabo de Hornos was rigged and operated as a beam trawler, the trawl opening area and geometry should be more stable compared to using trawl doors as on RV Kronprins Haakon.

On both vessels all fish specimens were separated from the rest of the catch and sorted. Total weight (Marel M2200/PL2220 marine scale) per tow and taxon as well as individual lengths were measured following standard IMR methodology^21–23^. Four species of myctophids (*Electrona antarctica*, *Gymnoscopelus fraseri*, *Krefftichthys anderssoni*, and *Protomyctophum bolini*) were sexed, based on external characters (presence of supra- and infracaudal luminous glands, or shape of the antorbital luminous organ). The fish catch on FV Cabo de Hornos was photographed, and most early life stage specimens, often hard to identify, were preserved in 96% ethanol for later examination and verification on land. Ethanol is an ideal preservation solution for samples to be analyzed using molecular methods as it denatures DNA and prevents enzymatic degradation. However, it is well known that it also induces weight-loss and shrinkage. Therefore, weights and individual lengths should be handled with caution and are probably not directly comparable to the data obtained from RV Kronprins Haakon. Species identification followed Gon and Heemstra^11^ and North and Kellermann^24^, with additional information from *e.g*. CCAMLR^25^, Figueroa and Ehrlich^26^, Iwami and Naganobu^27^, Kawaguchi and Butler^28^, Nakamuro and Parin^29^ and Smith and Heemstra^30^. Validity of scientific names was checked with Fricke *et al*.^31^, systematic order is according to Van der Laan *et al*.^32^.

Onboard the RV Kronprins Haakon images of each species, sometimes including separate images of specific characters, were captured with a Canon EOS 70D camera using a Canon EFS 60 mm f/2.8 Macro objective, mounted on a monopod. The images were imported to Photoshop CC 2019 for editing and annotation. Each taxon (species or genus, depending on lowest identifiable taxonomic resolution) is illustrated by an image with the main taxonomic characters highlighted. Characters defining order and family level are surrounded by a black frame and only provided on images of the first species of the family.

Tissue samples for mitochondrial DNA barcoding were taken from the catches on RV Kronprins Haakon to substantiate morphological identification methods. The tissue samples were preserved in 96% ethanol and processed following Mateos-Rivera *et al*.^33^. DNA was isolated either from the removed larval eyes or a piece of the larval tissue and placed in individual wells of a 200 µL 96 well-plates (Axygen Scientific, CA, USA) containing 75 µL of a solution 5% Chelex 100 Resin (BioRad, CA, USA) and 15 µL of Proteinase K (Qiagen, Germany). The 96 well-plates were then incubated at 56 °C for 1 h followed by 10 min at 96 °C.

The mitochondrial COI gene was amplified using PCR in 12 µL reactions containing 2.4 µL 5x buffer, 1 µL of MgCl_2_ [25 mM], 1.92 µL dNTPs [1.25 mM], 1.44 µL 10 µM primer pair combination with the M13F sequence incorporated in the forward primers (cf. Ivanova *et al*.^34^), 0.07 µL GoTaq G2 DNA polymerase (Promega, WI, USA), 3.17 µL dH_2_O and 2 µL template DNA. The PCR conditions were 2 min at 95 °C, followed by 35 cycles of 30 s at 94 °C, 52 °C for 30 s and 1 min at 72 °C. The PCR ended with 10 min at 72 °C. PCR products were then purified by mixing 5 µL of the PCR product and 2 µL ExoSap-IT PCR product Cleanup (ThermoFisher, MA, USA) and an incubation at 37 °C for 15 min and 80 °C for 15 min. Finally, sequencing was performed using 1 µL of M13F primer [0.35 µM] at the sequencing facility at the University of Bergen. Sequence analyses were performed in Geneious v8.0.5 (Kearse *et al*.^35^). The resulting sequences were used as queries for BLASTn (see Altschul *et al*.^36^) against the NCBI database (www.ncbi.nlm.nih.gov/genbank) and compared to reference sequences to determine taxonomic identities. Although there were 211 tissue samples sequenced in total, only one sequence per species was uploaded to GenBank. This was decided due to i) the high similarity (being almost identical) between the sequences that seemingly belonged to the same species and; ii) not overload an already good existing database as all the species found during our study were already present in the GenBank repository. The only exception was *Muraenolepis* sp. (accession number PQ057126) where only one individual was found in our study.

## Data Records

The dataset is comprised of two comma-separated files submitted to PANGAEA of which the first is a station data file that also contains information on the catch. It holds the information on unique station number (*Serial number*), coordinates (*Latitude of Event, Longitude of Event*), Date/Time (UTC) (*Date/Time of Event*) of the trawl stations as well as maximum fishing depth (*Depth water bottom/maximum*) and gear used (*Gear ID*). Furthermore, it provides the scientific name of the taxon (*Taxon/taxa, unique identification*), taxonomic codes (Aphia ID*: Taxon/taxa, unique identification (Semantic URI))*, the catch sample ID (*Sample ID*) a unique number per taxon per station), information on ontogenetic stage (*Life stage*: larvae, juvenile, adult, subadult), the conservation method (*Preservation*: fresh or 96% ethanol), catch weight (*Total catch*) and count (*Total counts, per catch*), type of length measurement (*Variable*: total length or standard length), as well as number of specimens length measured (*Fish, wet mass*), their associated number (*Number of individuals*). The *Sample number* indicates if a standard sample has been undertaken or measured (e.g. length) alternatively an additional sample has been undertaken such as for instance, *sex*. The second file contains length resolution (*Precision*), individual length (*Fish, standard length*, *Fish, total length, Fish preanal length*), *Sex*, and information whether a tissue sample (*Tissue, sampling*) was obtained from a given individual or not. The two files are linked by *Serial number* and S*ample ID*. Attention is drawn to the significance and meaning of the variable *Sample number* which is termed part-of-a-sample or aliquot in *IMR’s Handbook on field sampling*^22^ or “delprøve” in the Norwegian edition^23^. In the current material this variable holds a value of 1, 2 or 3. *Sample number* notifies a special sample collected that relates to a given taxonomic category (*Taxon/taxa, unique identification*) and *Serial number*. For instance, a species having a *Sample number* equal to 2 or 3, could mean that also sex was determined for a selected number of adult specimens, or it could be juveniles that were analysed separately and given its own *Sample number*. If for a given taxonomic category (e.g. *Electrona antarctica*) we aim to calculate the weight or number of individuals per volume of water filtered (m^3^) for a given *Serial number*, one needs to sum up the respective values for each identified *Sample number* for it to be representative for total catch. For many scientists, keeping juveniles as a separate category to their adult counterparts can give valuable information, although it is many times hard to catch both categories quantitatively with the same gear. Regarding the material collected by FV Cabo de Hornos, care should be exercised as some taxonomic categories and *Sample numbers* have missing weights. Although all fishes from a catch were registered and photographed, not all specimens were preserved and stored. As mentioned earlier, weights and lengths reported here should be treated carefully as specimens were mostly measured following preservation in 96% ethanol and are therefore not directly comparable to data collected on RV Kronprins Haakon. Conversion equations between fresh and ethanol preserved specimens as used for the myctophid *Benthosema glaciale* (see Knutsen *et al*.^37^) could be applied, as could also traditional length-weight relationship for other Antarctic fish species to facilitate conversion to fresh lengths and weights (cf. Wang *et al*.^38^).

The NMD Biotic database used by the Institute of Marine Research in Bergen (IMR), Norway is designed to meet all possible requirements of the various surveys conducted by the Institute, and therefore not all data fields are necessarily relevant or used. It depends on the cruise type and data to be acquire/collected. Hence, to simplify data transfer to a public repository a significant reduction of data fields has been undertaken without losing key information. However, some fields have also been added. The two first fields added are variants of the same measure, *Trawling distance* in meters [m] and nautical miles [nmi] respectively. These two variables can be used for standardizing data as can also the third variable added, namely water volume filtered by the trawl in [m^3^] (*Water volume, filtered*). See Methods for details on how these variables have been derived. Associated with each taxonomic category is an annotated image of that same taxonomic category [*Image, specimens*], a JPG image that also holds information of size and specific characters of the category it refers too. We have also added a reference to accession numbers in GenBank (*Accession number, genetics*), for those individuals from which sequences were uploaded. Similarly, another column variable (*Voucher Specimen Code*) refers to the collection ID of the specimens deposited in the ichthyology collection of the University Museum of Bergen (Department of Natural History, Bergen, Norway). GenBank accession numbers (PQ057120 - PQ057146) for the COI genes and species identified are shown in Table S2.

The datasets with links to the high-resolution annotated images presented in this work can be found at PANGAEA - Data Publisher for Earth and Environmental Science (cf. Knutsen *et al*.^39,40^). Annotated images are also available in Supplementary File 1. Further inquiries can be directed to the corresponding authors.

## Technical Validation

The data submitted to PANGAEA have been extracted from the *NMD Biotic* database at IMR. The data have been subject to thorough quality control and validation from the point of collection, laboratory at sea and on land analyses, to storing data in our local data repository. Subsequent to initial entry into *NMD Biotic*, several manual and automated data checks were performed to ensure that the entered data were of prime quality. This included but was not restricted to checking for taxonomic updates, missing information, duplicate entries, typos, spelling errors, inconsistencies, valid parameters, and value ranges. The taxonomic data obtained from RV Kronprins Haakon was all worked up at sea by the experienced fish taxonomist and co-author Dr. Rupert Wienerroither, or under his direct supervision using appropriate literature^11,24–30^. Scientific names and taxonomy were checked against Eschmeyer’s Catalog of Fishes^31^ and WoRMS^41^ to ensure taxonomic consistency. Taxonomic identification of the biological material from FV Cabo de Hornos was carried out by trained zooplankton personnel, with substantial training and experience also in fish taxonomy. For additional control all fish specimens were photographed onboard, and bulk or individual specimens fixed and brought back to the onshore laboratory for verification. Of a total of 6133 fish specimens caught, 1548 were not identified to species, but to higher taxonomic levels, i.e. genus or family level (seven genera and two family-level accounts). All of the latter specimens were either juvenile, badly damaged, of unresolved taxonomic status, or only documented by picture. A selection of them were sequenced, and it is considered likely that the entire species diversity of the two surveys was covered. Of the 56 identified taxa, 27 were sequenced for the COI gene (several specimens per taxa). This was done to verify the morphologically often difficult specimens, because of missing description of characters, or impossible to identify juveniles of the genera *Artedidraco*, *Bassango*, *Dissostichus*, *Electrona*, *Lepidonotothen*, *Muraenolepis*, *Notolepis*, *Notothenia*, *Pogonophryne*, *Pseudochaenichthys*, and *Trematomus*. It was also done for some adult specimens where the taxonomic characters were partly unclear or controversial like *Bathylagus* and *Nansenia*. Genetic analyses confirmed the morphological identification of juvenile *Chaenocephalus*, *Chionodraco*, *Pagetodes*, and *Prionodraco*, as well as some adult *Gymnoscopelus* and *Protomyctophum*. For the rest (29 taxa) morphological identification was unambiguous and no genetic samples were taken.

## Usage Notes

The main objective of this paper is to allow researchers the complete access to the taxonomic fish data, a first step in a follow up production of a fish atlas for the study area. This will aid future taxonomic work so that data can be re-analysed with objectives that might be completely different from the ones they were collected for. Therefore, raw data are provided so that each researcher may analyse them using appropriate methodologies that could differ from those used in previous studies but also use them as a future baseline for the distribution of taxa across the Scotia Sea while also acting as an aid to others in their taxonomic work to reduce uncertainty in future and similar taxonomic assessment work.

However, we recommend paying attention to some of the specificities from the two vessels that are described here. With different vessels having undertaken work that in principle should be close to identical, there are always some small differences that might matter when trying to understand and reanalyse such data. Even though the trawls were identical for the two vessels, rigging of the trawls were not. Different crews could possibly also affect how vessels were operated, and trawls deployed, even if instructions and information from the science PIs were identical. If the aim is to standardize catches, differences like these could affect vertical haul speed, towing times, and calculated filtered trawl volumes.

A key issue to be aware of is the two different trawls that were used on RV Kronprins Haakon. The Macroplankton trawl is non-graded while the Harstad trawl is graded, having decreasing mesh sizes from the opening to the cod-end of the net. Thus, it is advised to avoid direct comparison of Macroplankton and Harstad trawl catches. The variable mesh size of the Harstad trawl makes it difficult to understand and quantify the trawl selectivity for a given species over its size range. For example, two fish species with the same total body length could escape through the trawl meshes at different positions along the trawl length because one species has a far better swimming capacity or is significantly slimmer than the other. Hence, the position along the length of the trawl of what should be considered “the trawl opening area” could be quite different for the two species. This task and its implications could be addressed during carefully designed field experiments. However, this is far beyond the scope of the present work. For the abovementioned reasons, we have not calculated filtered volumes for the Harstad trawl hauls in the present work.

Supplementary File 1 contains photos of all taxa identified, annotated with taxonomic characters. For the more abundant species also length histograms and distribution maps are given. Although symbols and colours used in the maps consistently represent trawl type and juvenile/adult specimens, please be aware that the size of the symbols varies from species to species. This provides a better visualization of differences in numbers of specimens caught. Same symbol sizes used throughout the maps would result in only small symbols on many maps and some larger ones in maps of the few dominant species.

## Supplementary information

Supplementary Information: Taxonomic characteristics, fish distribution and catches across the Scotia Sea

## Data Availability

No custom code was used to generate or process the data described in this paper.
