# Supplementary material for: Fish species identification and composition across the Scotia Sea during austral summer 2019
Source: Sci Data. 2025 May 30;12:916. doi: 10.1038/s41597-025-05217-z (PMC12125196; doi:10.1038/s41597-025-05217-z)
Supplement: Supplementary file 1 — Supplementary Information: Taxonomic characteristics, fish distribution and catches across the Scotia Sea [file 41597_2025_5217_MOESM1_ESM.pdf]

Identification of fish taxa collected across the Scotia Sea during the austral summer of 2019

Tor Knutsen[1], Merete Kvalsund[1], Rupert Wienerroither[1], Kjell Bakkeplass[1], Julio Erices[1], Nicolas Straube[2], Monica Bente Martinussen[1], Alejandro Mateos-Rivera[1], Alina Rey[1], Jon Rønning[1], Georg Skaret[1] & Bjørn Arne Krafft[1]

1. Institute of Marine Research, Bergen, Norway
corresponding author(s): Tor Knutsen (tor.knutsen@hi.no) and Bjørn Arne Krafft (bjorn.krafft@hi.no).
2. Department of Natural History, University Museum of Bergen, Bergen, Norway.

Images of each species were captured with a Canon EOS 70D camera and imported to Photoshop CC 2019 for editing and annotation of taxonomic characters. The species accounts basically include an image with the main taxonomic characters, a map, and a histogram of the length distribution. Characters at higher taxonomic level (order and family) are bordered by a black frame and only provided on the image of the first species of the family. Maps are presented for species that were identified at more than two stations, otherwise the station information is given in the text. The size range of the symbols varies from species to species. Length histograms are based on data from R/V Kronprins Haakon only and are presented for species with an abundance of more than 20 specimens, otherwise or in case of a very narrow length range, the data is given in the text. The histograms show differences between the two trawl types and for four species of myctophids also the length distributions based on sex are shown.

Congridae - conger eels

Bassanago albescens (Barnard, 1923) - hairy conger

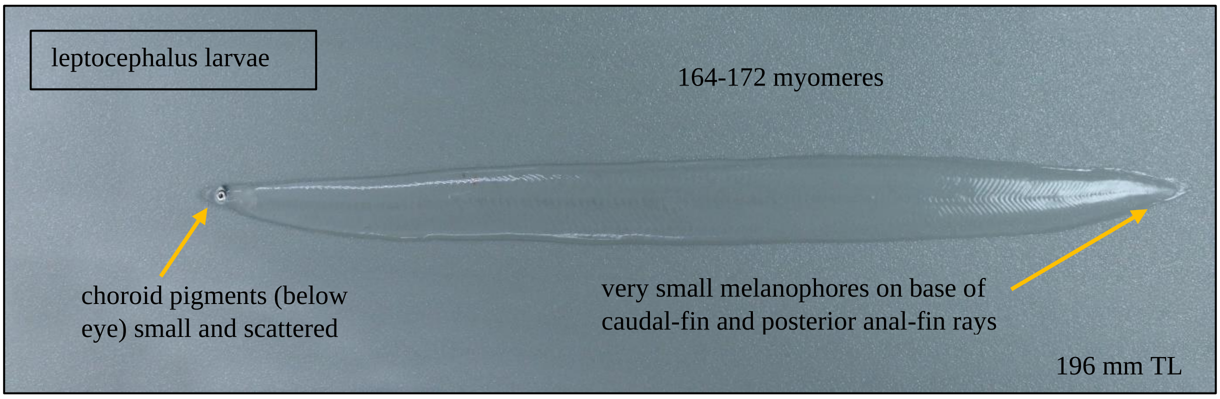

Two specimens were caught at station 4051 and 4060, 179 and 196 mm TL.

Microstomatidae - pencilsmelts

Nansenia antarctica Kawaguchi and Butler, 1984

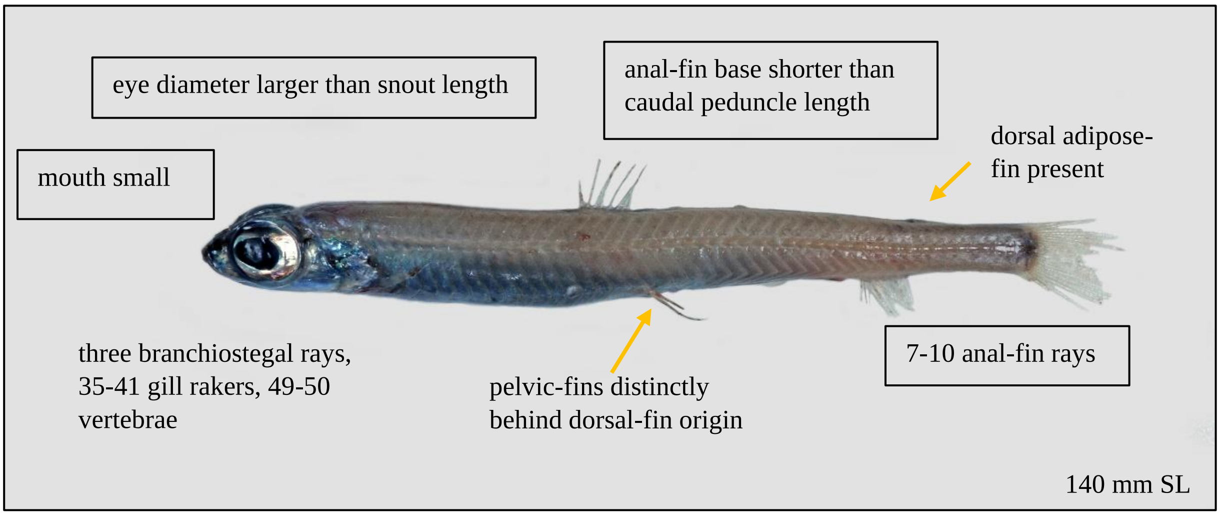

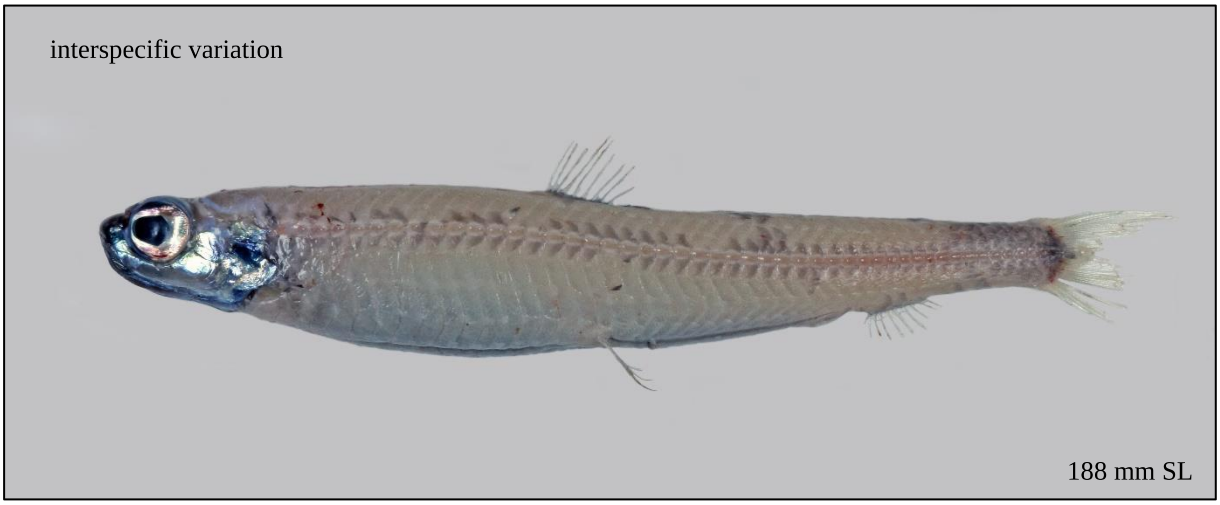

Two specimens were caught at station 4033.

Bathylagidae - deepsea smelts

Bathylagus spp.- juveniles and indet.

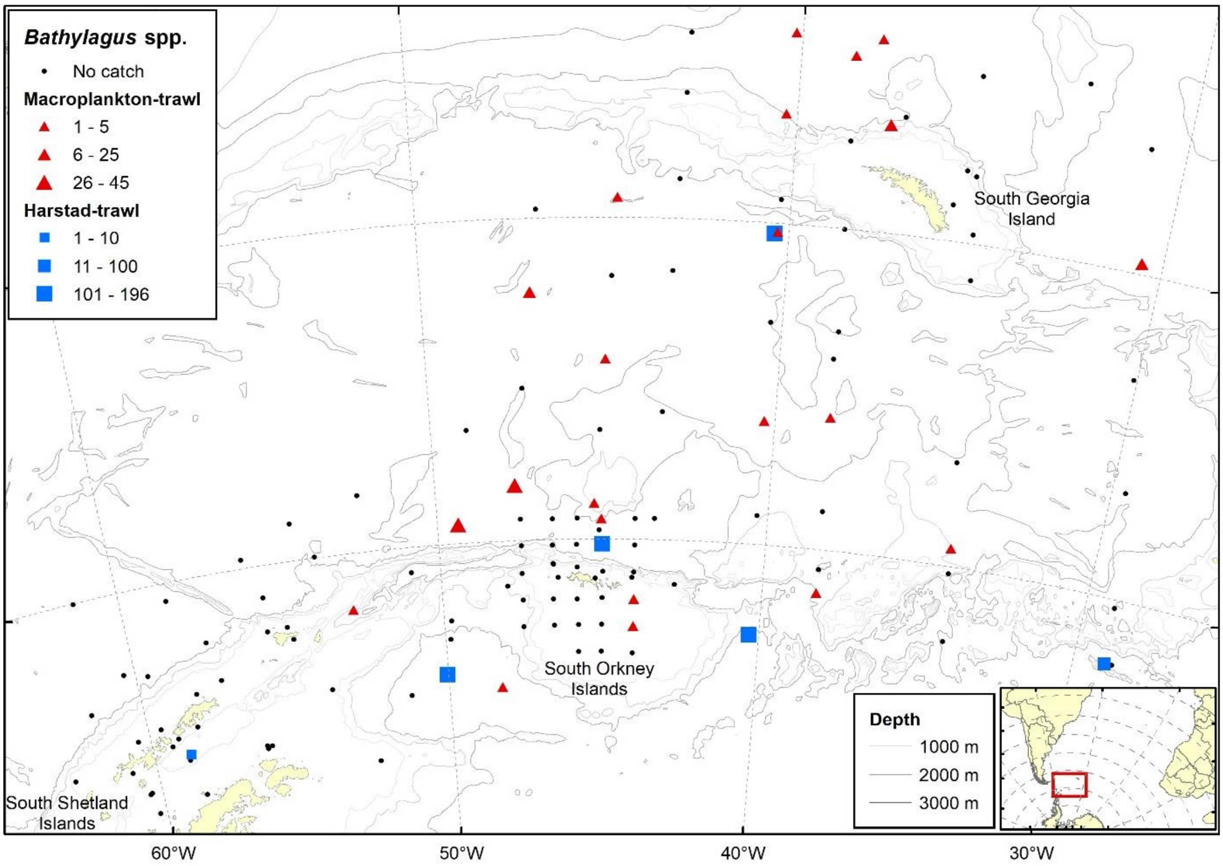

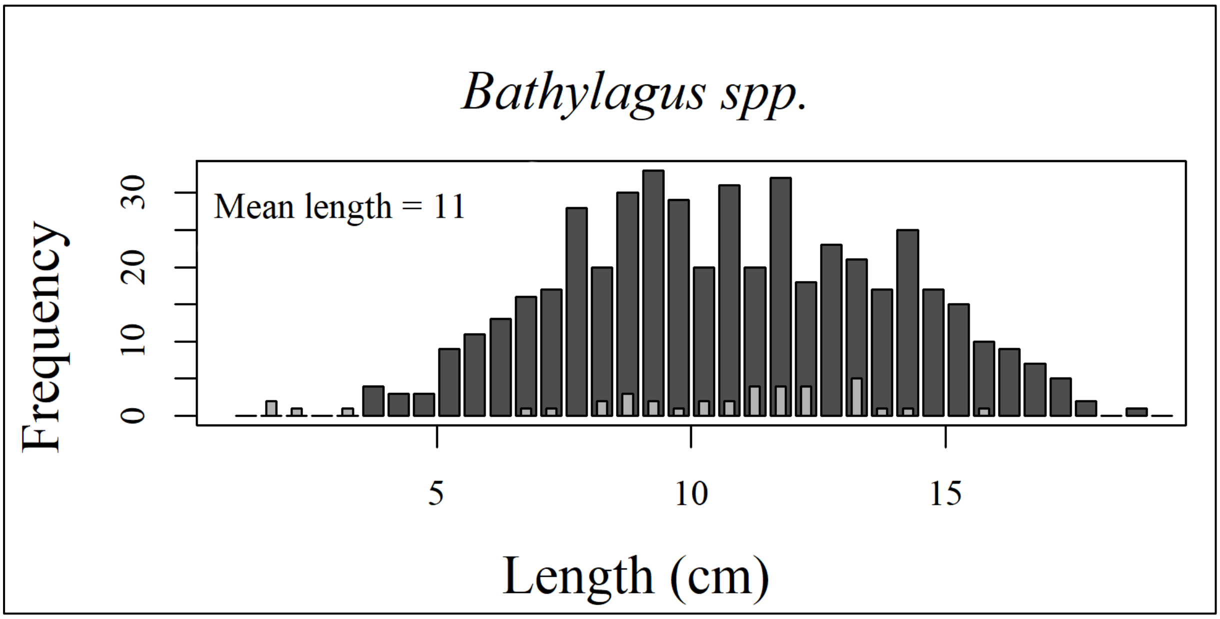

Length distribution of *Bathylagus* spp., light grey: Macroplanktontrawl (N = 38), dark grey: Harstadtrawl (N = 489).

The taxonomic differences between *B. antarcticus* and *B. tenuis* are mainly based on meristic and morphometric characters. These are often overlapping and difficult to use in the field. The

identification to species level in the present work is exclusively based on DNA sequencing data. From a total of 845 specimens, genetic samples were arbitrarily taken of 128 specimens and then sequenced, 118 turned out to be *B. antarcticus*, and ten to be *B. tenuis*.

Bathylagidae - deepsea smelts

Bathylagus antarcticus Günther, 1878 - Antarctic deepsea smelt

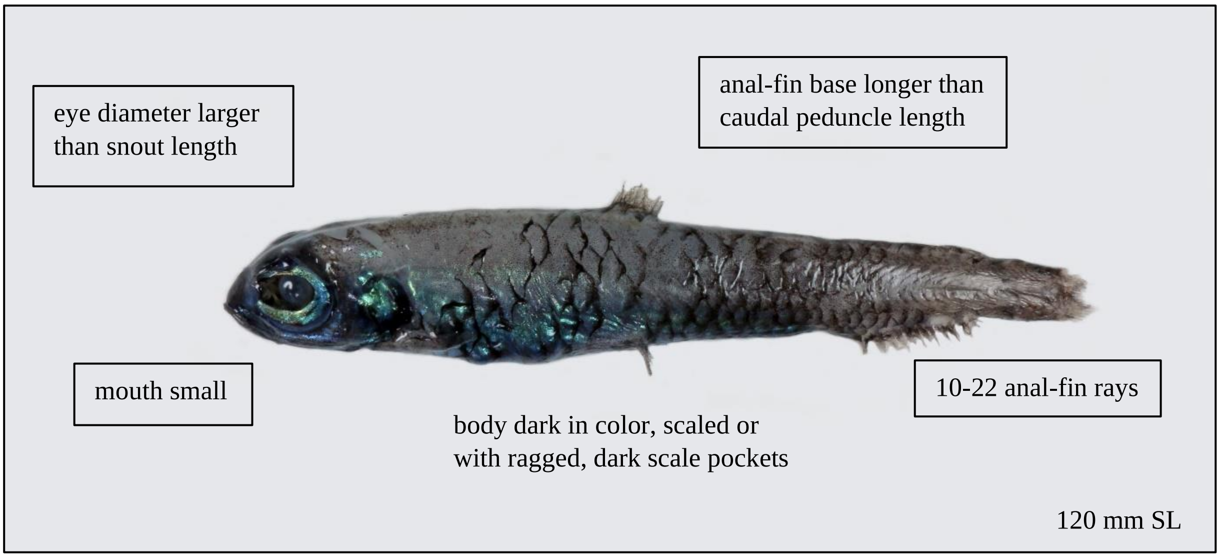

The 118 genetically verified specimens were caught at eight different stations (4009, 4034, 4038, 4042, 4047, 4060, 4065, and 4067), indicating presence of the species over large parts of the area. Their lengths ranged from 31 to 179 mm SL, mean 111 mm.

Bathylagidae - deepsea smelts

Bathylagus tenuis Kobyliansky, 1986

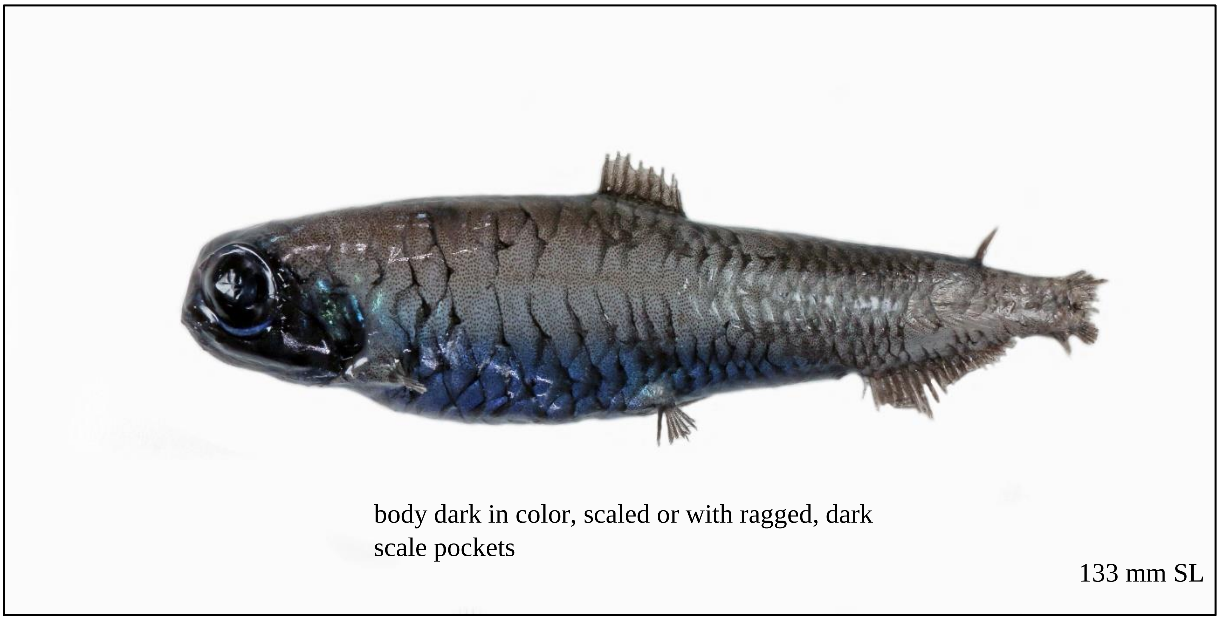

The ten genetically verified specimens were caught at three different stations (4034, 4044, and

4047), but this might not reflect the actual distribution of the species. Their lengths ranged from 106 to 158 mm SL, mean 131 mm.

Gonostomatidae - bristlemouths

Cyclothone microdon (Günther, 1878) - veiled anglemouth

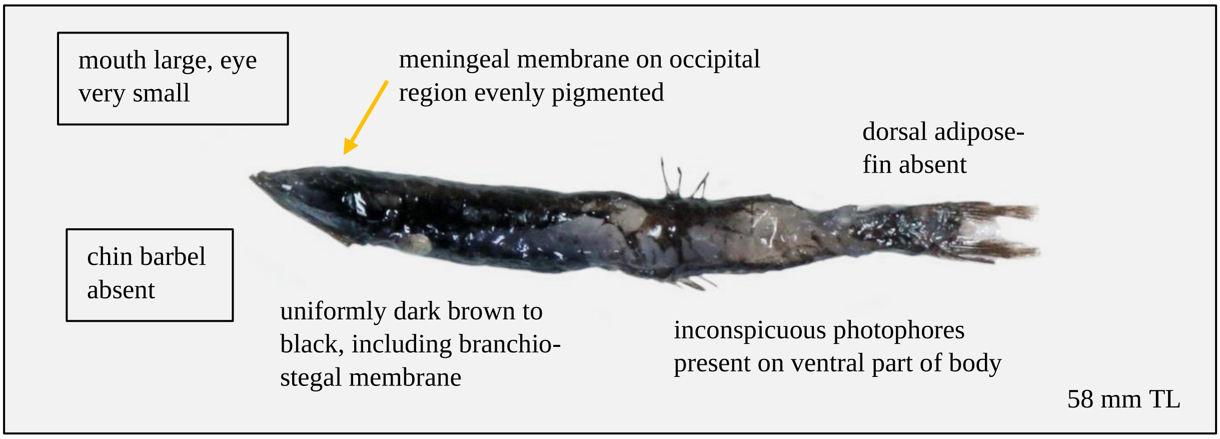

Twenty specimens were caught, ranging in length from 41 to 61 mm SL.

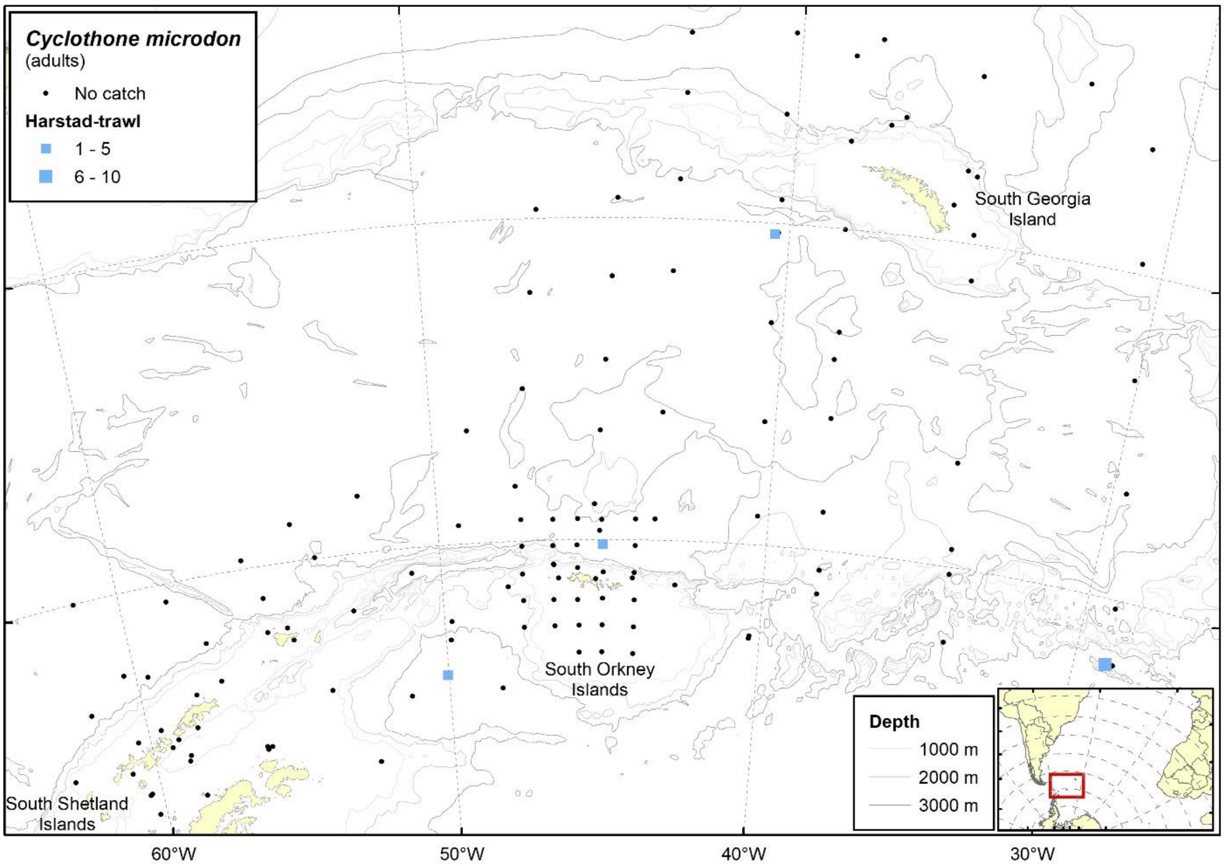

Stomiidae - dragonfishes

Borostomias antarcticus (Lönnberg, 1905) – snaggletooth

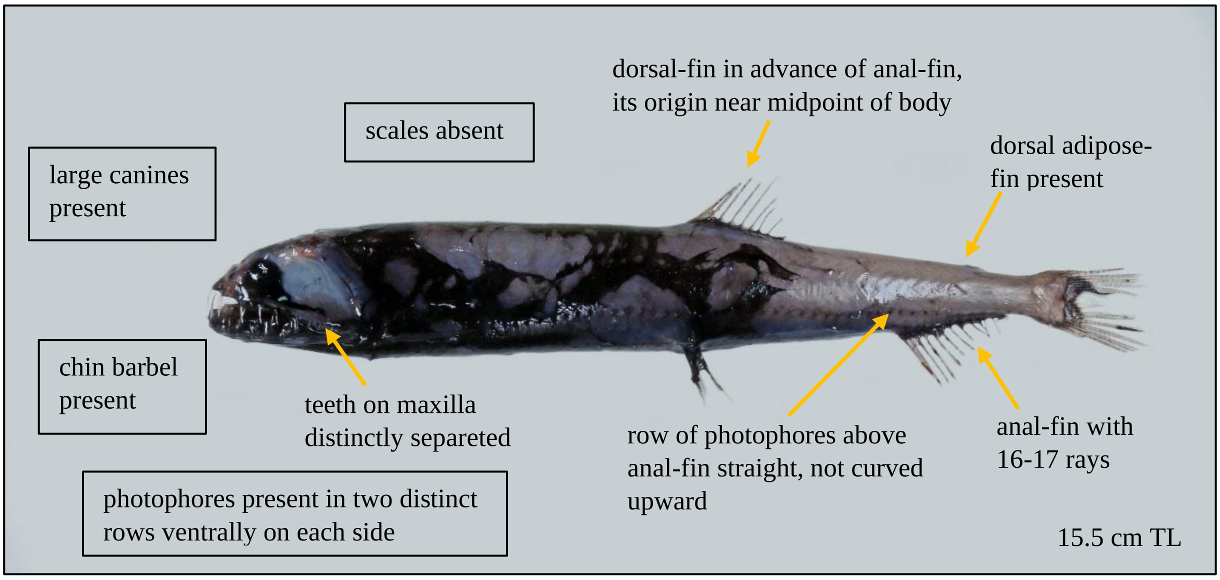

Nine specimens were caught at station 4033 and 4047, ranging in length from 13.5 to 25 cm TL.

Stomiidae - dragonfishes

Stomias gracilis Garman, 1899 - boa dragonfish

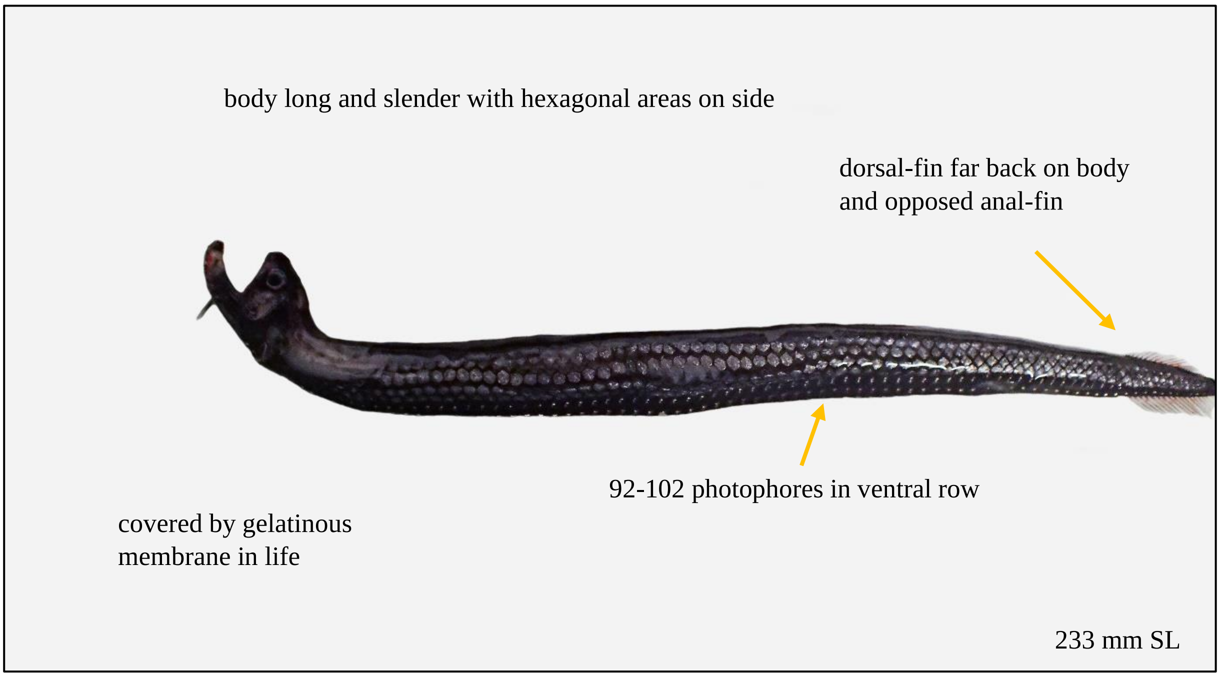

Three specimens were caught at station 4304 and 4306, ranging in length from 160 to 233 mm TL.

Scopelarchidae - pearleyes

Benthalbella elongata (Norman, 1937)

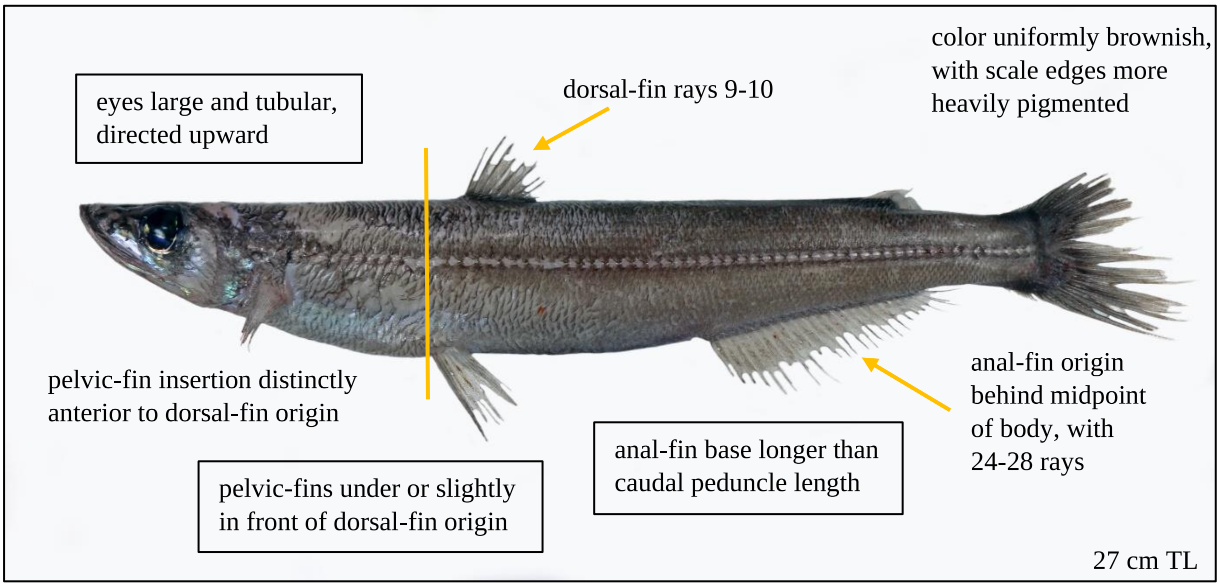

Three specimens were caught at station 4033, ranging in length from 16.5 to 27 cm TL.

Paralepididae - barracudinas

Notolepis spp. - juveniles

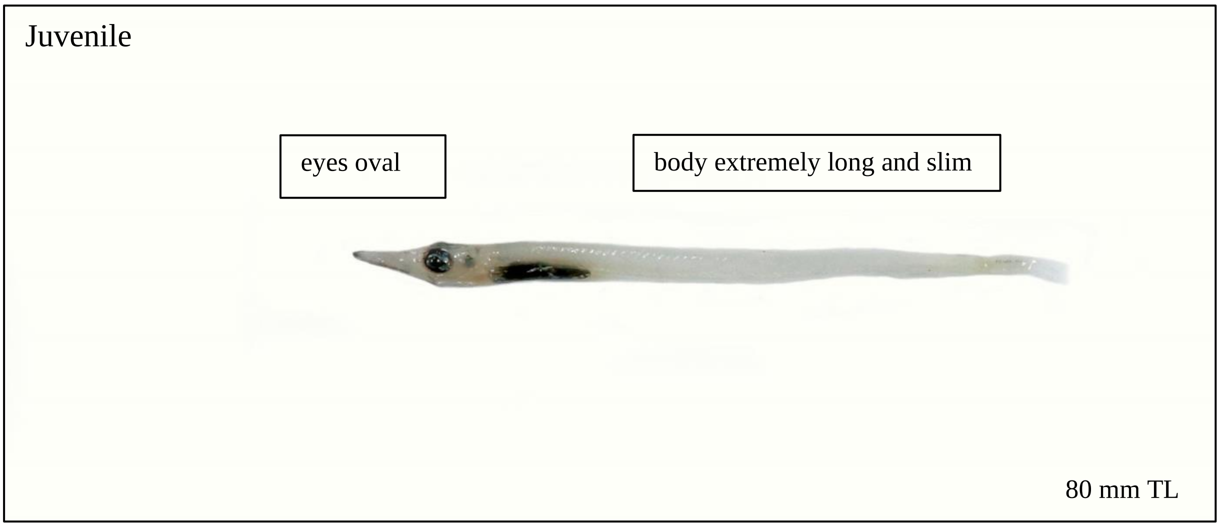

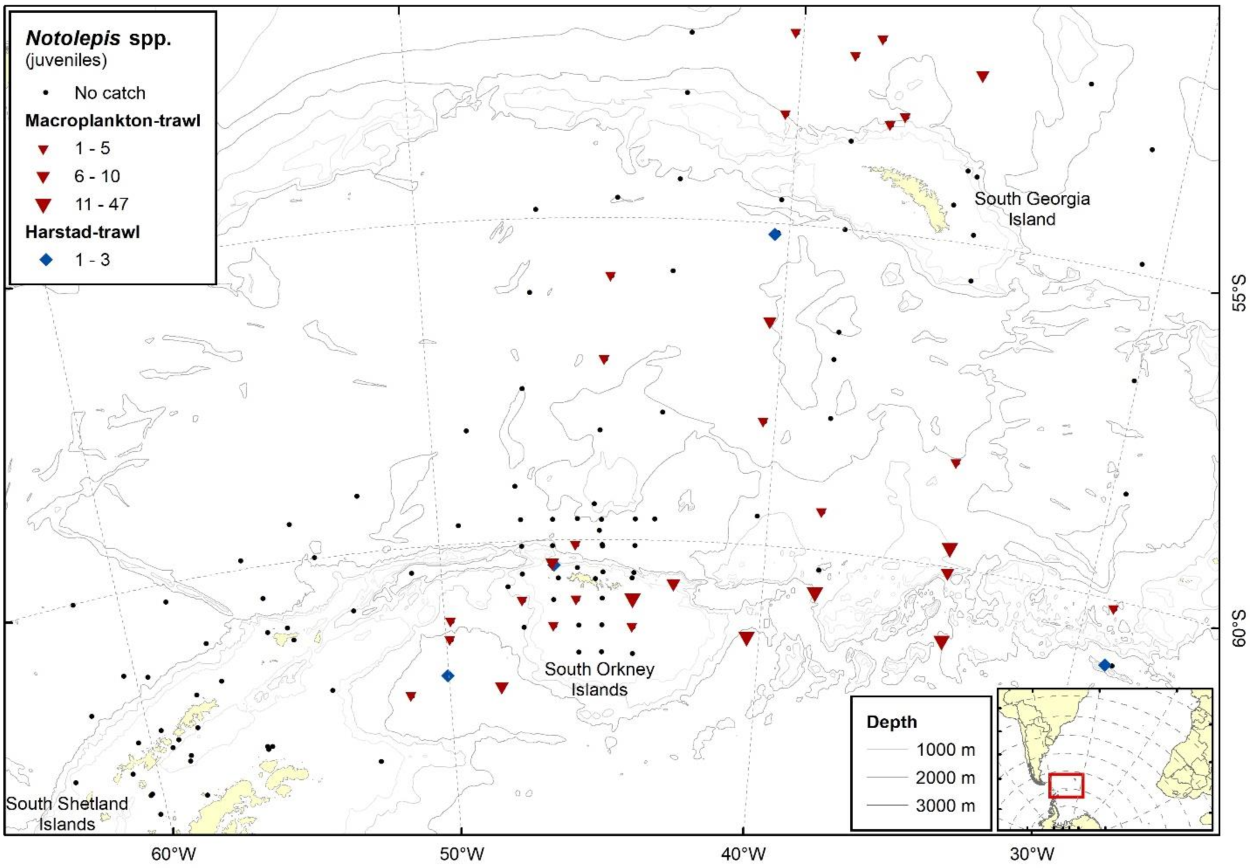

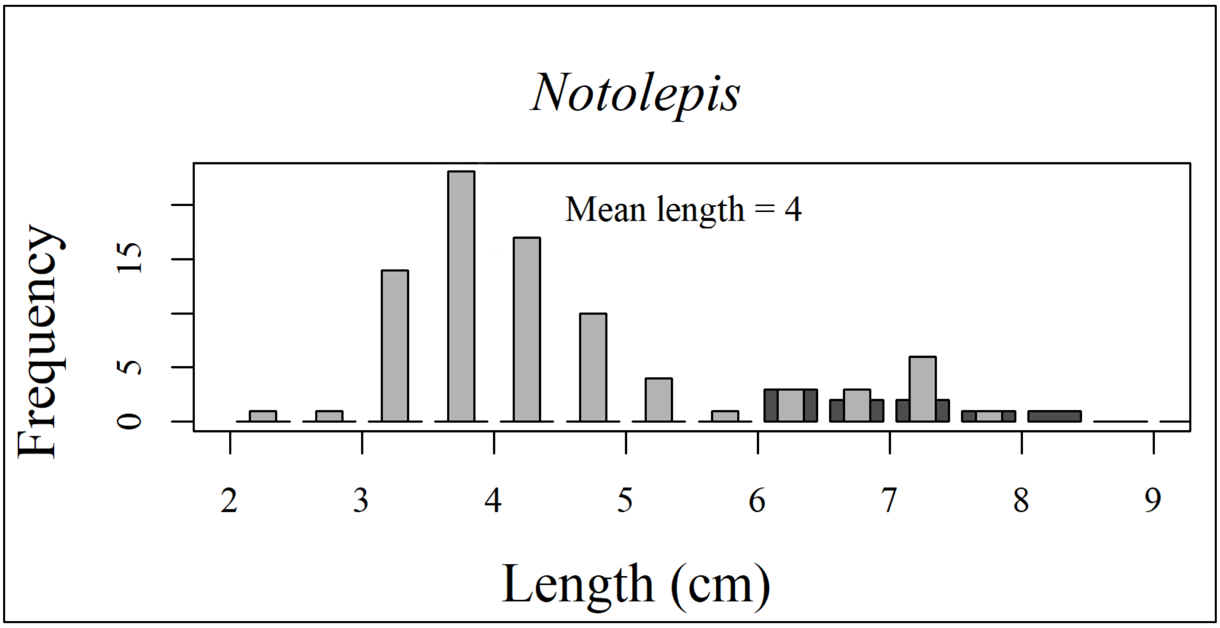

Length distribution of *Notolepis* spp., light grey: Macroplanktontrawl (N = 84), dark grey: Harstadtrawl (N = 9).

Paralepididae - barracudinas

Notolepis annulata Post, 1978 - ringed barracudina

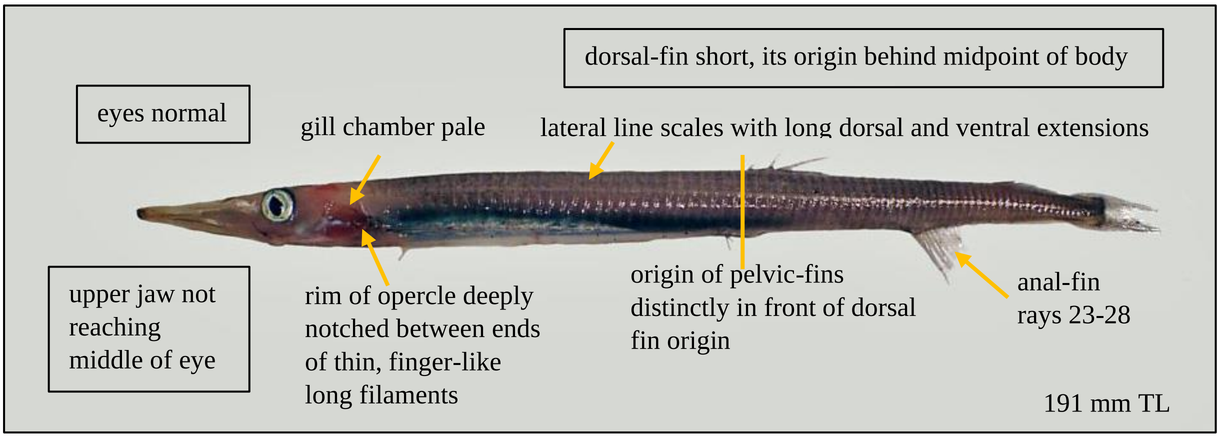

Two specimens were caught at station 4287, 78 and 191 mm TL.

Paralepididae - barracudinas

Notolepis coatsorum Dollo, 1908 - Antarctic jonasfish

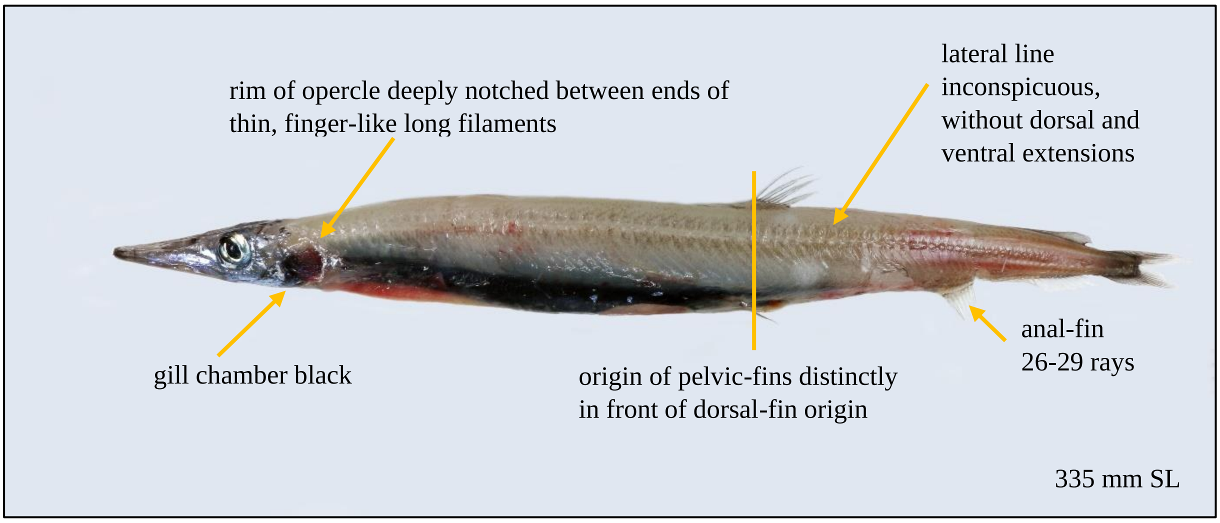

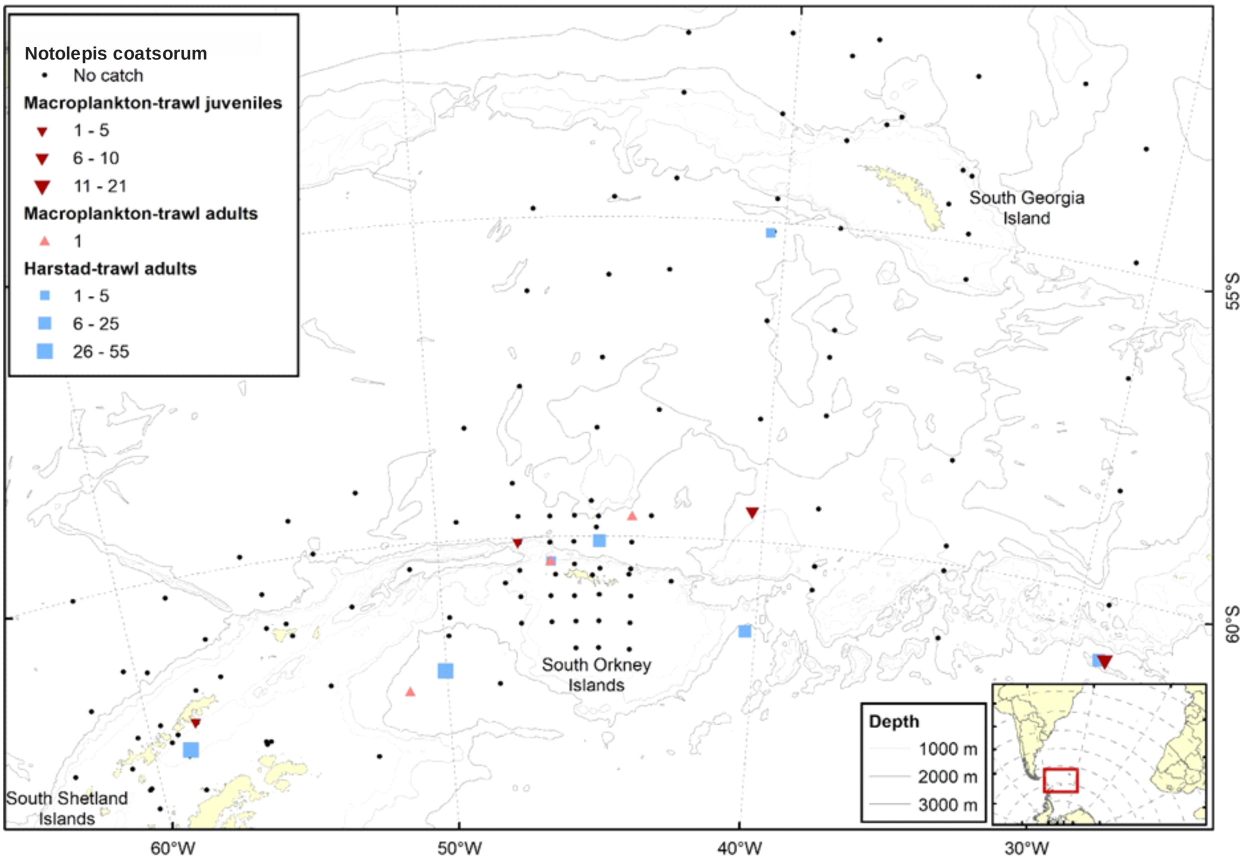

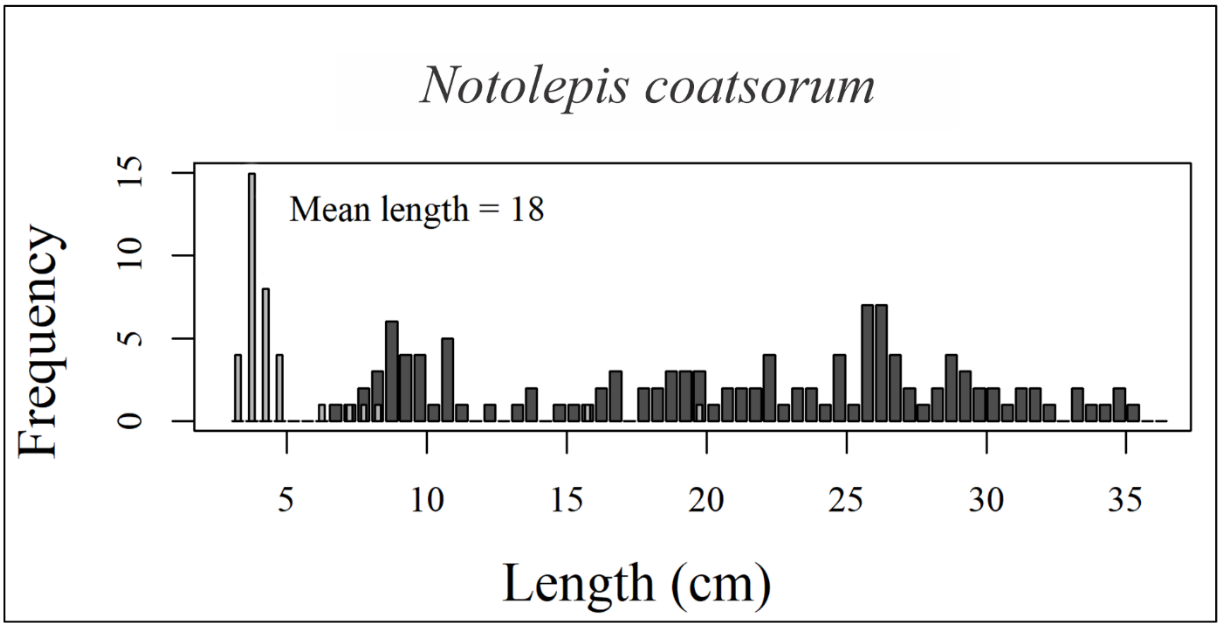

Length distribution of *N. coatsorum*, light grey: Macroplanktontrawl (N = 37), dark grey: Harstadtrawl (N = 122). The species identification of 21 out of the 35 juveniles measuring less than 10 cm caught with the Macroplanktontrawl at stations 4001, 4012, 4043, and 4064 was genetically verified.

Myctophidae - lanternfishes
Electrona spp. - juveniles

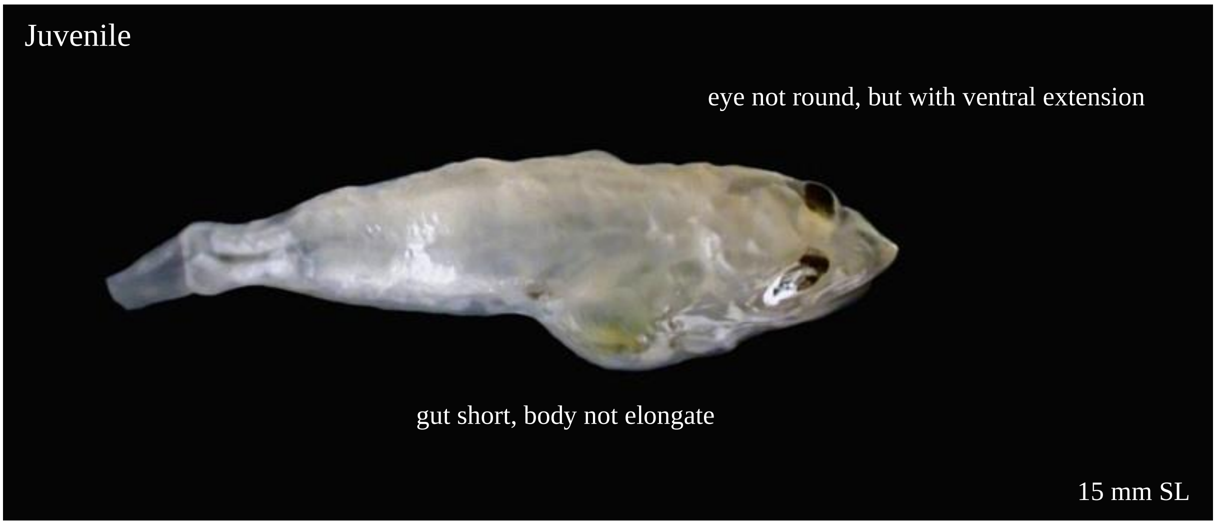

Thirty-eight specimens were caught, ranging in length from 11 to 24 mm SL, mean 15.5 mm SL.

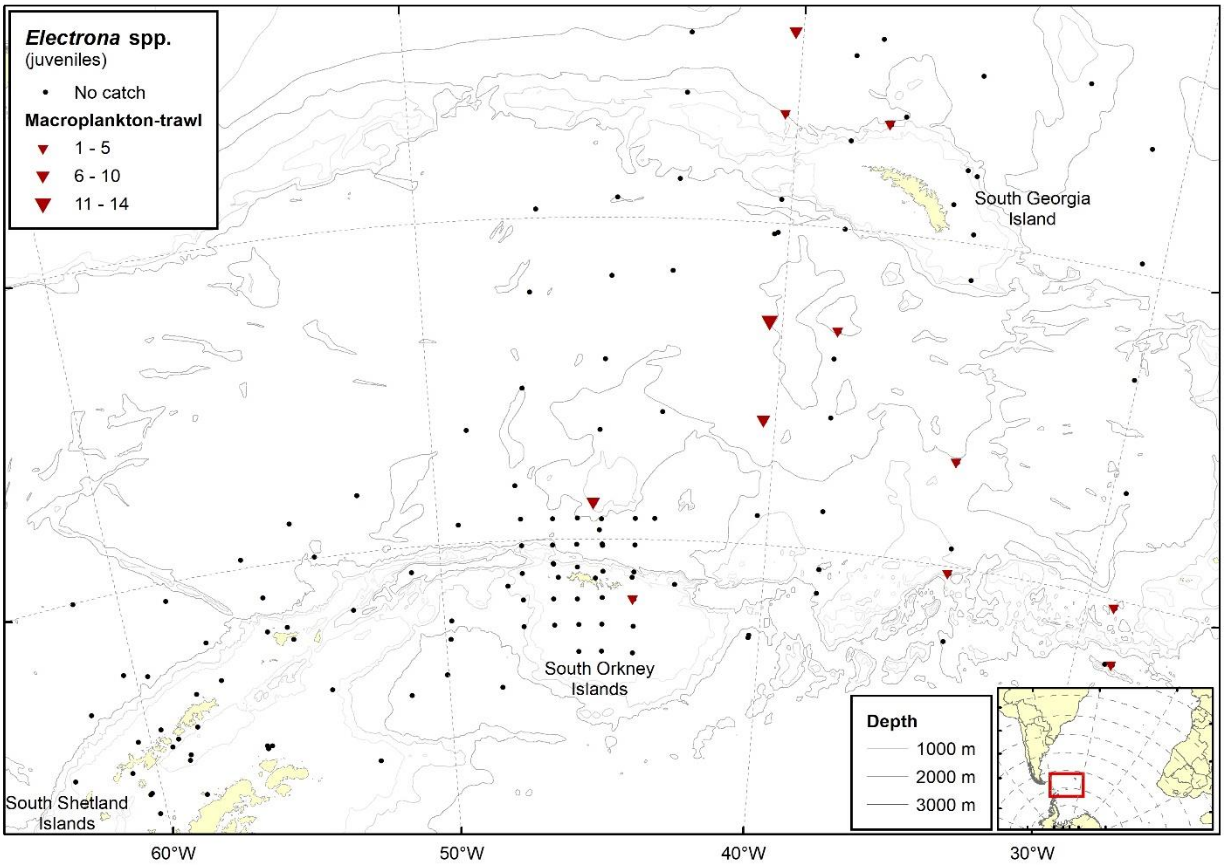

Myctophidae - lanternfishes

Electrona spp. - indet.

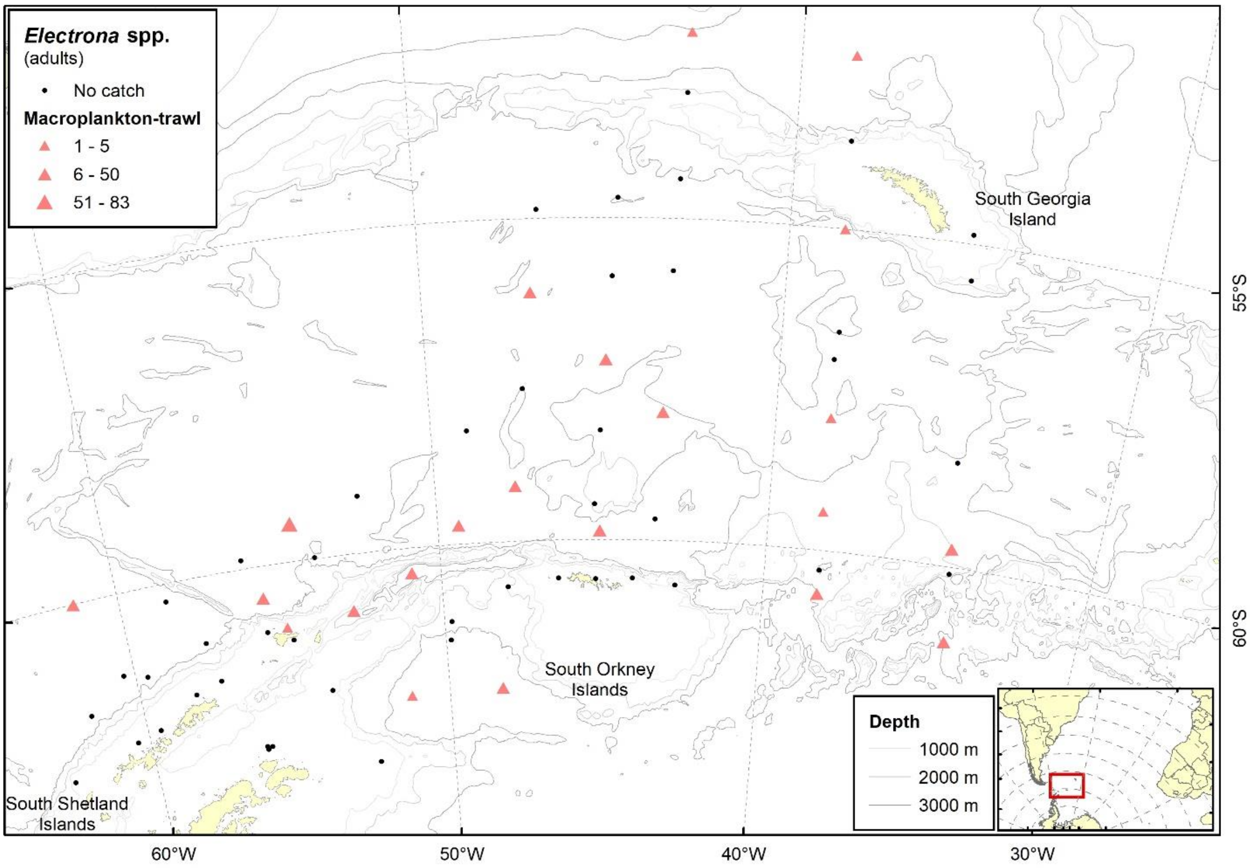

254 specimens taken on FV *Cabo de Hornos* could - based on pictures - not be verified to species level.

Myctophidae - lanternfishes

Electrona antarctica (Günther, 1878) - Antarctic lanternfish

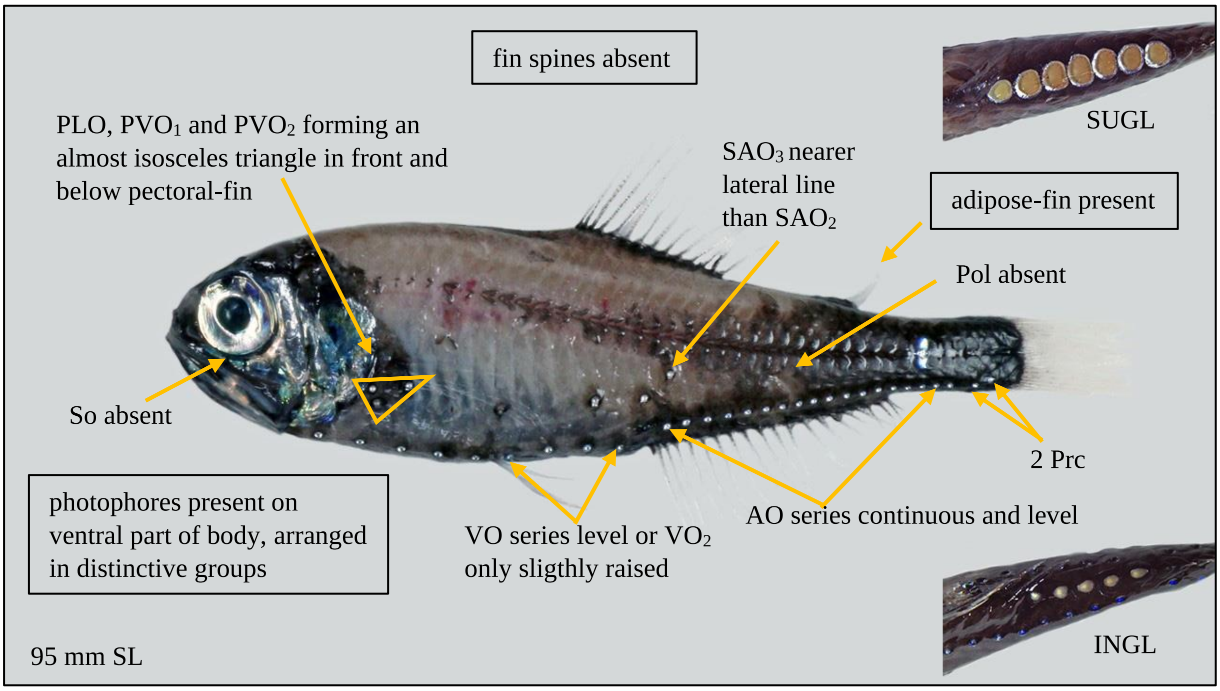

Inset: supracaudal luminous gland (SUGL) of male dorsally, and infracaudal luminous gland (INGL) of female ventrally on caudal peduncle.

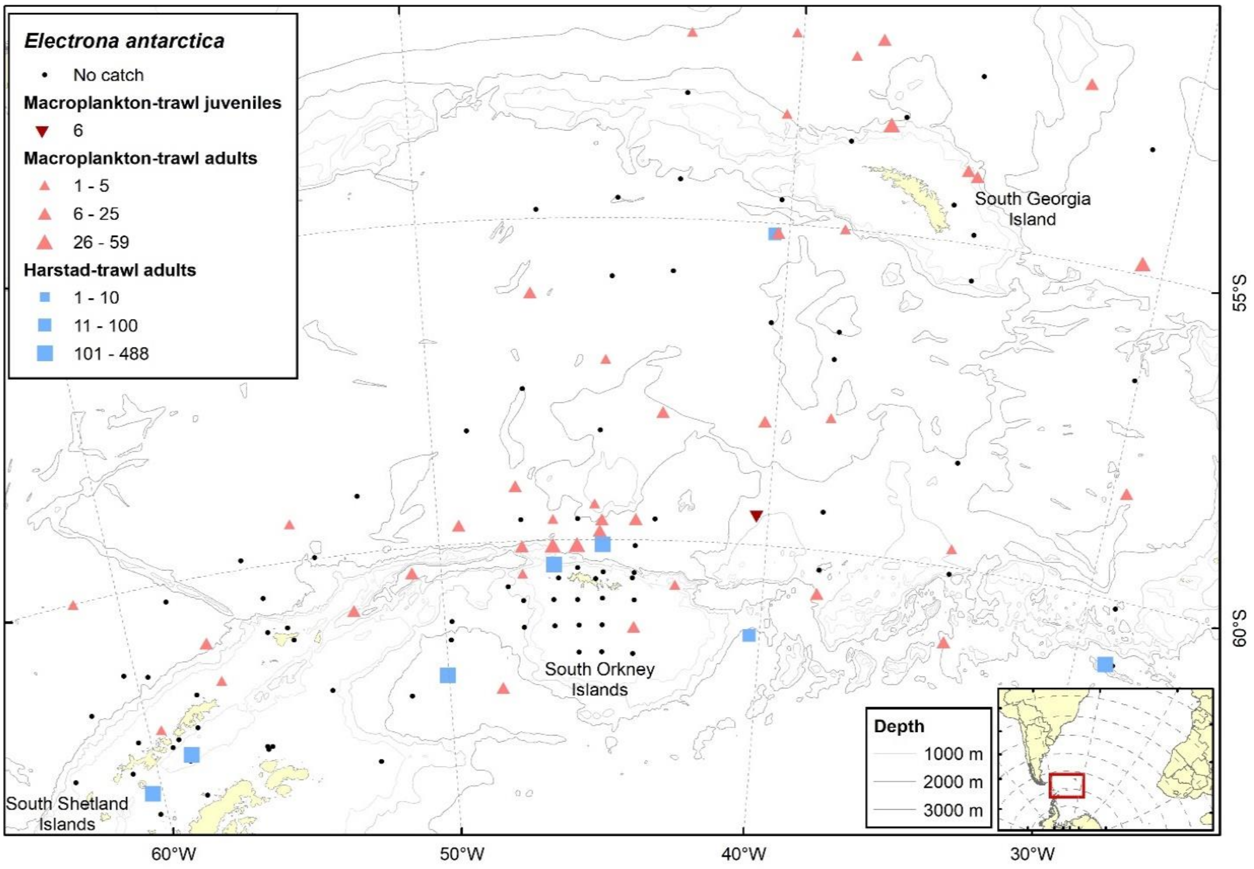

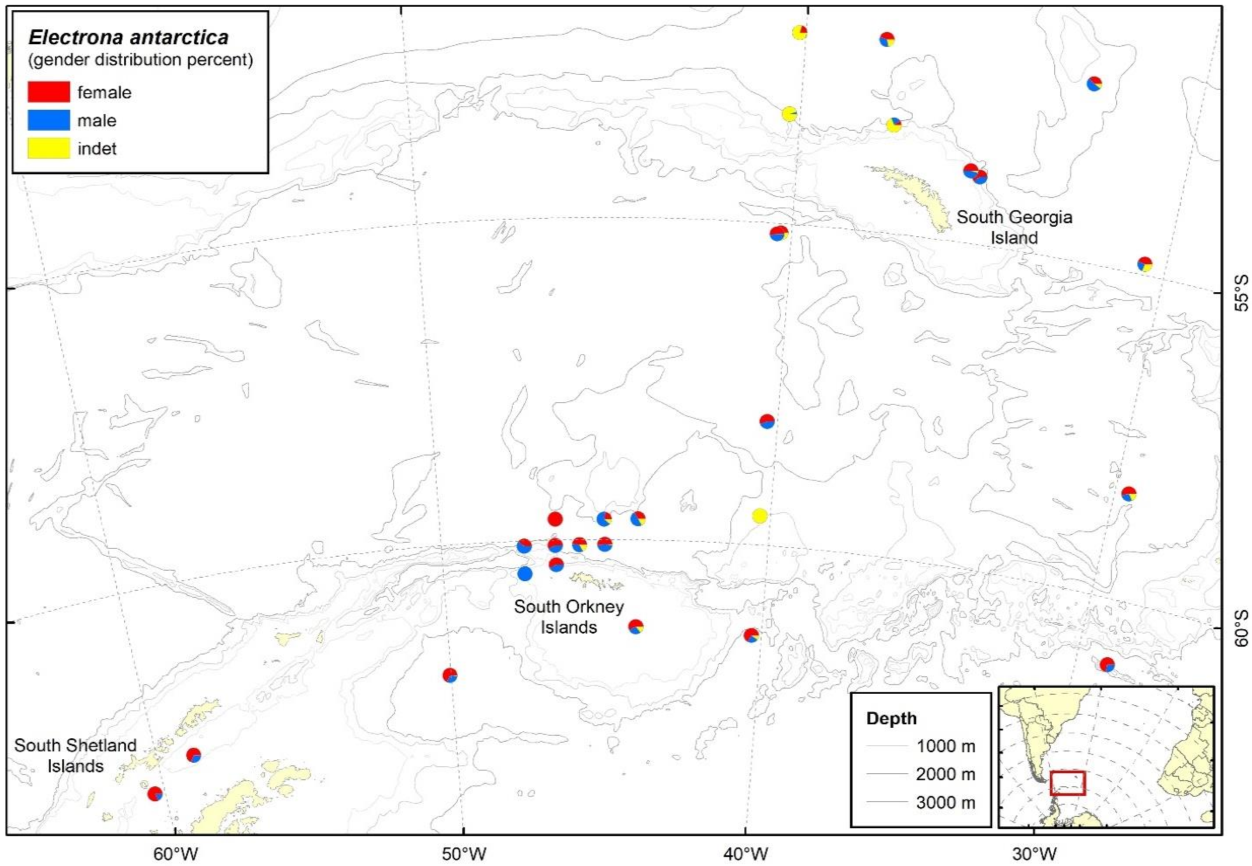

Electrona antarctica
(gender distribution percent)
female
male
indet
South Georgia Island
South Orkney Islands
South Shetland Islands
Depth
1000 m
2000 m
3000 m
55°S
60°S
60°W
50°W
40°W
30°W

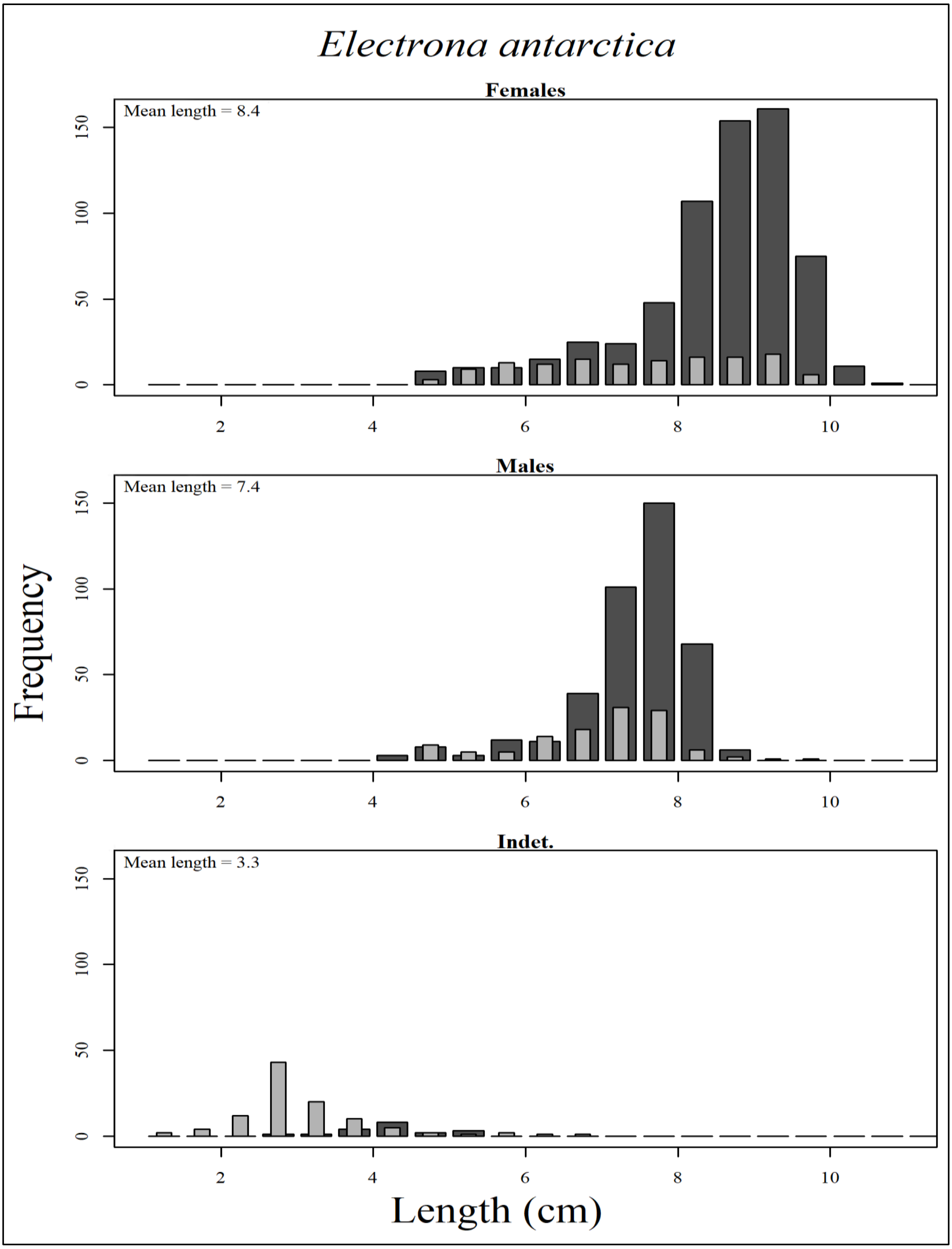

Length distribution of *E. antarctica*, light grey: Macroplanktontrawl, dark grey: Harstadtrawl, N = 142+649 (females), 119+401 (males), 97+19 (indet.). The species identification of five out of six juveniles measuring less than 2 cm caught at station 4043 was genetically verified.

Myctophidae - lanternfishes

Electrona carlsbergi (Tåning, 1932) - electron subantarctic

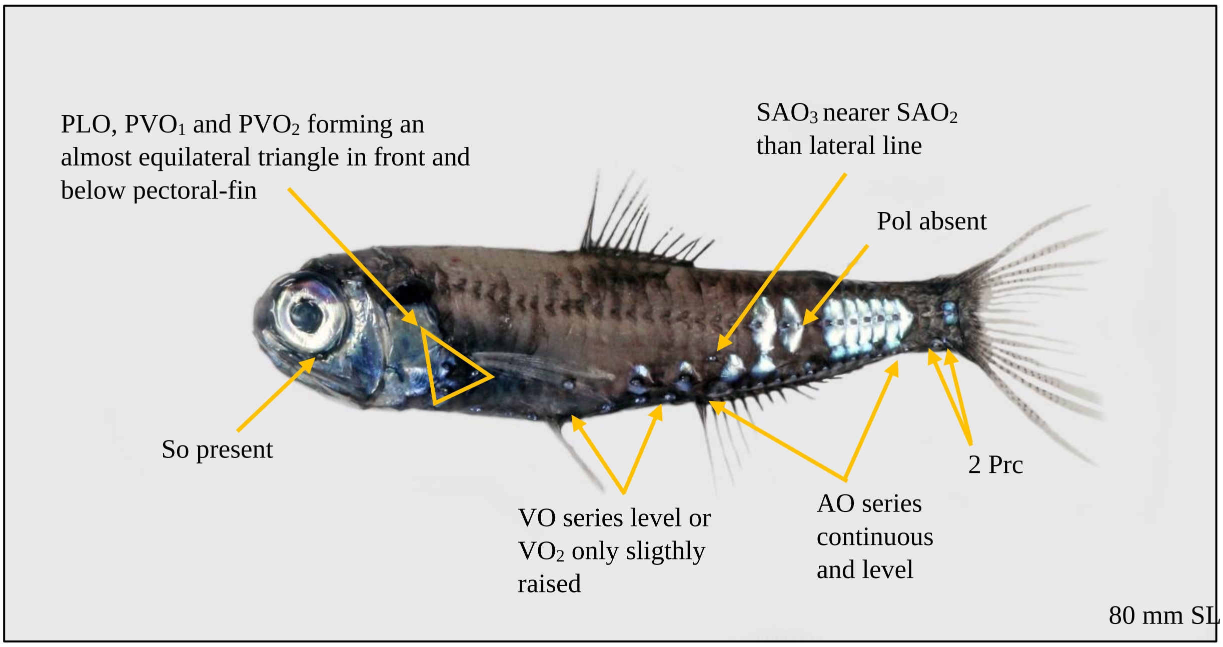

Here 122 specimens were length measured, ranging in length from 70-87 mm SL, mean 76 mm.

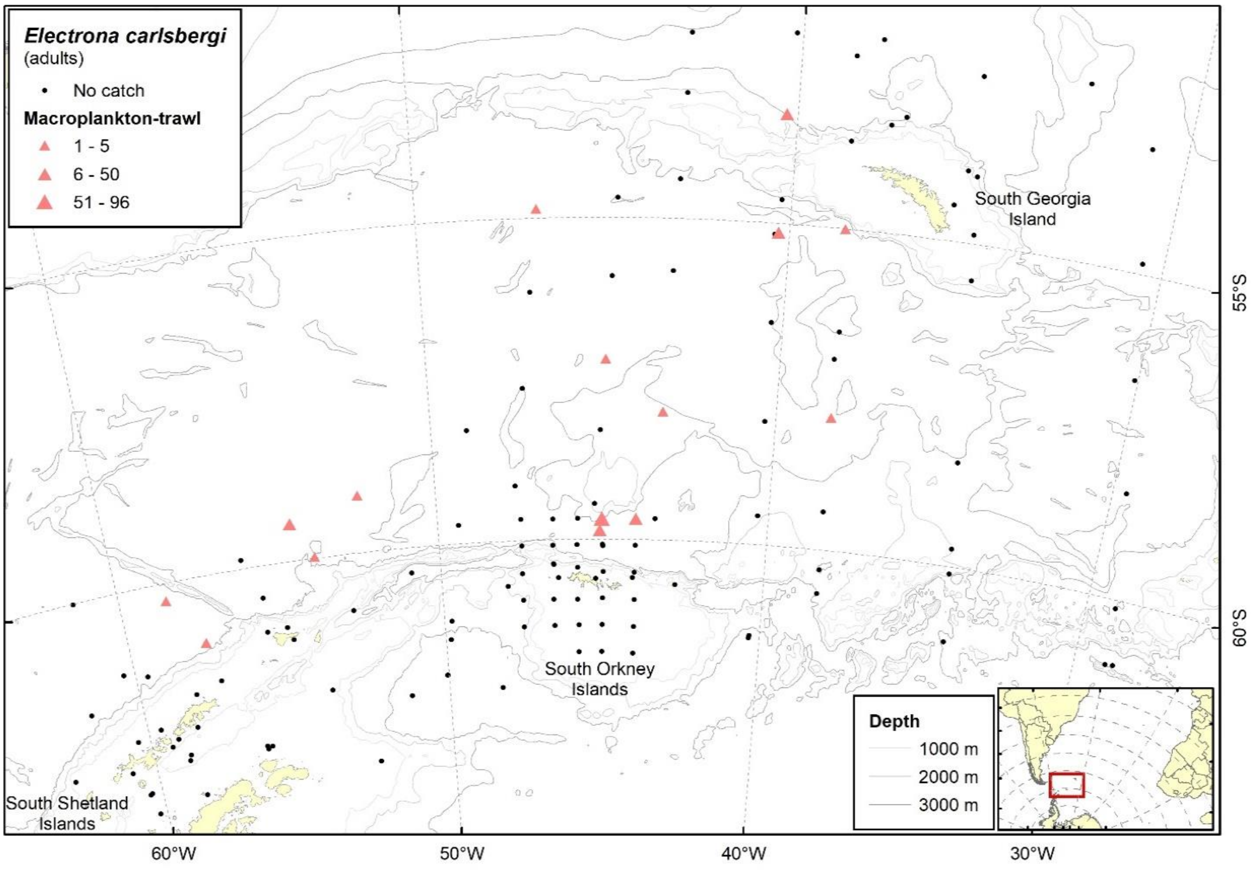

Myctophidae - lanternfishes

Electrona subaspera (Günther, 1864) - rough lanternfish

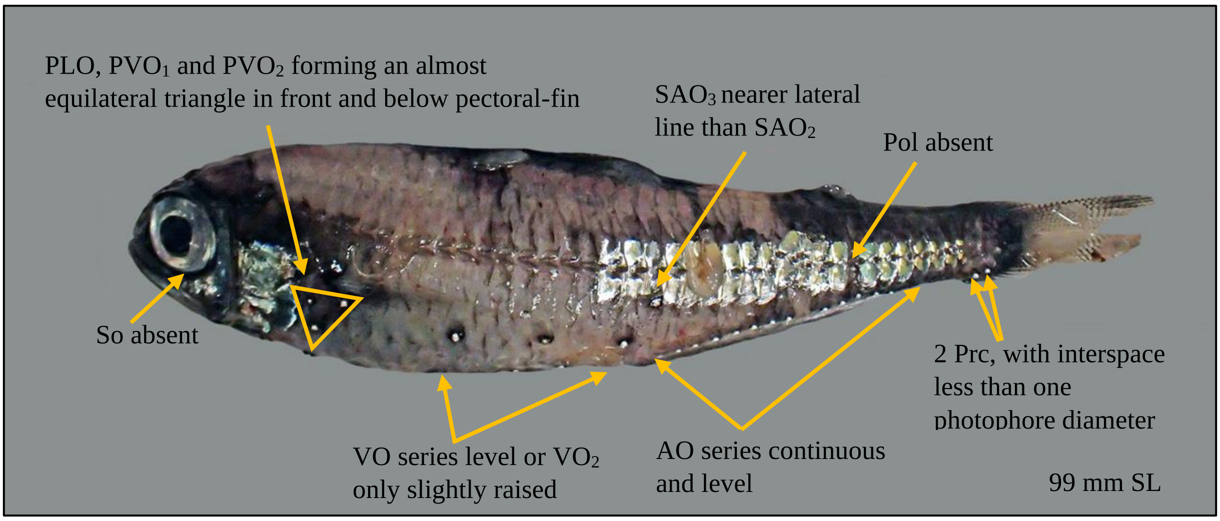

One specimen was caught at station 4304.

Myctophidae - lanternfishes

Gymnoscopelus spp. - indet.

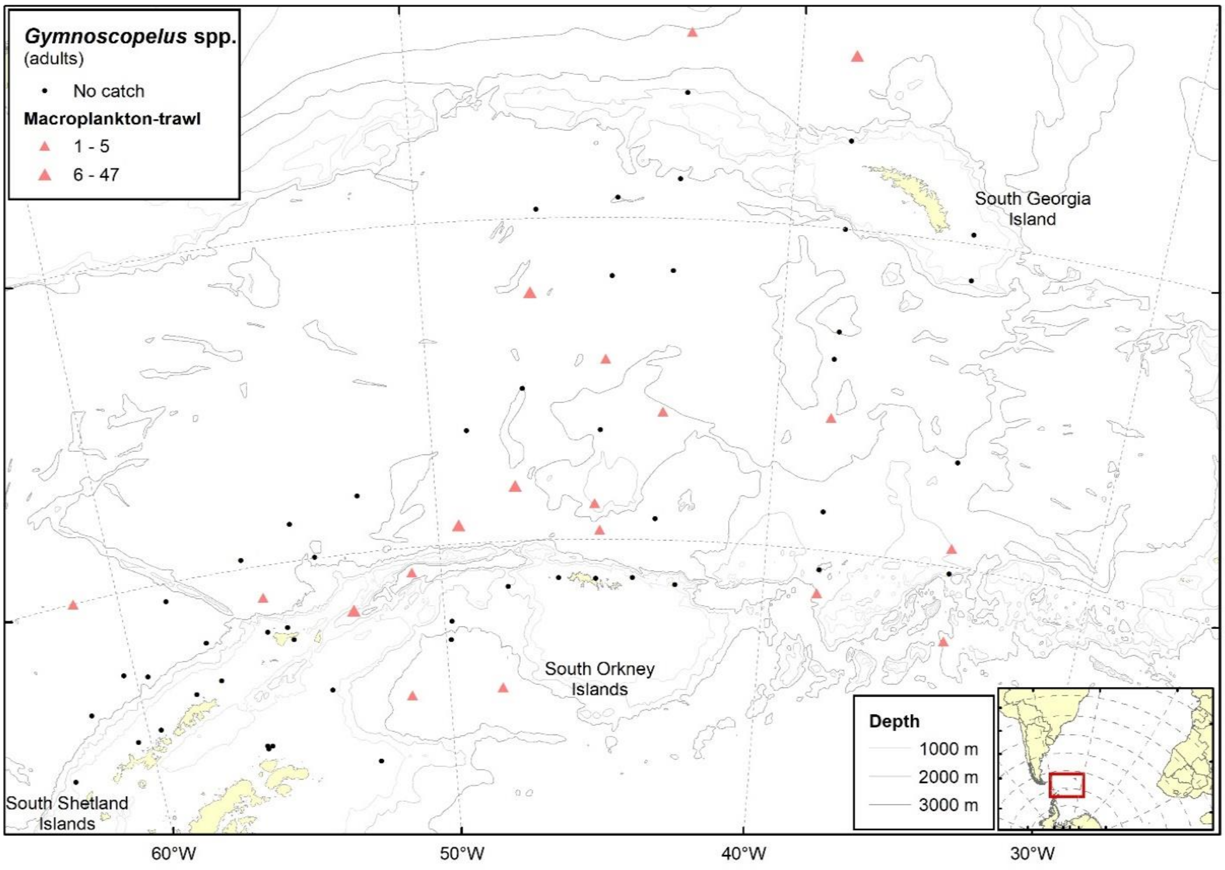

Here 181 specimens taken on FV *Cabo de Hornos* could - based on pictures - not be verified to species level.

Myctophidae - lanternfishes

Gymnoscopelus bolini Andriashev, 1962 - grand lanternfish

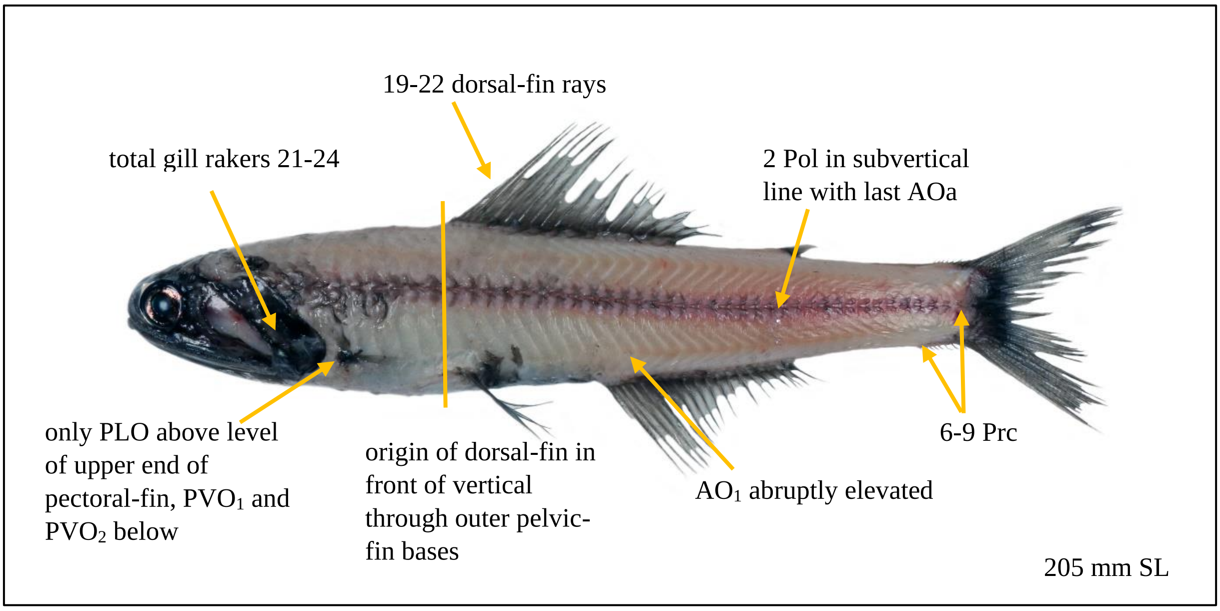

Seven specimens were caught, ranging in length from 147 to 230 mm SL.

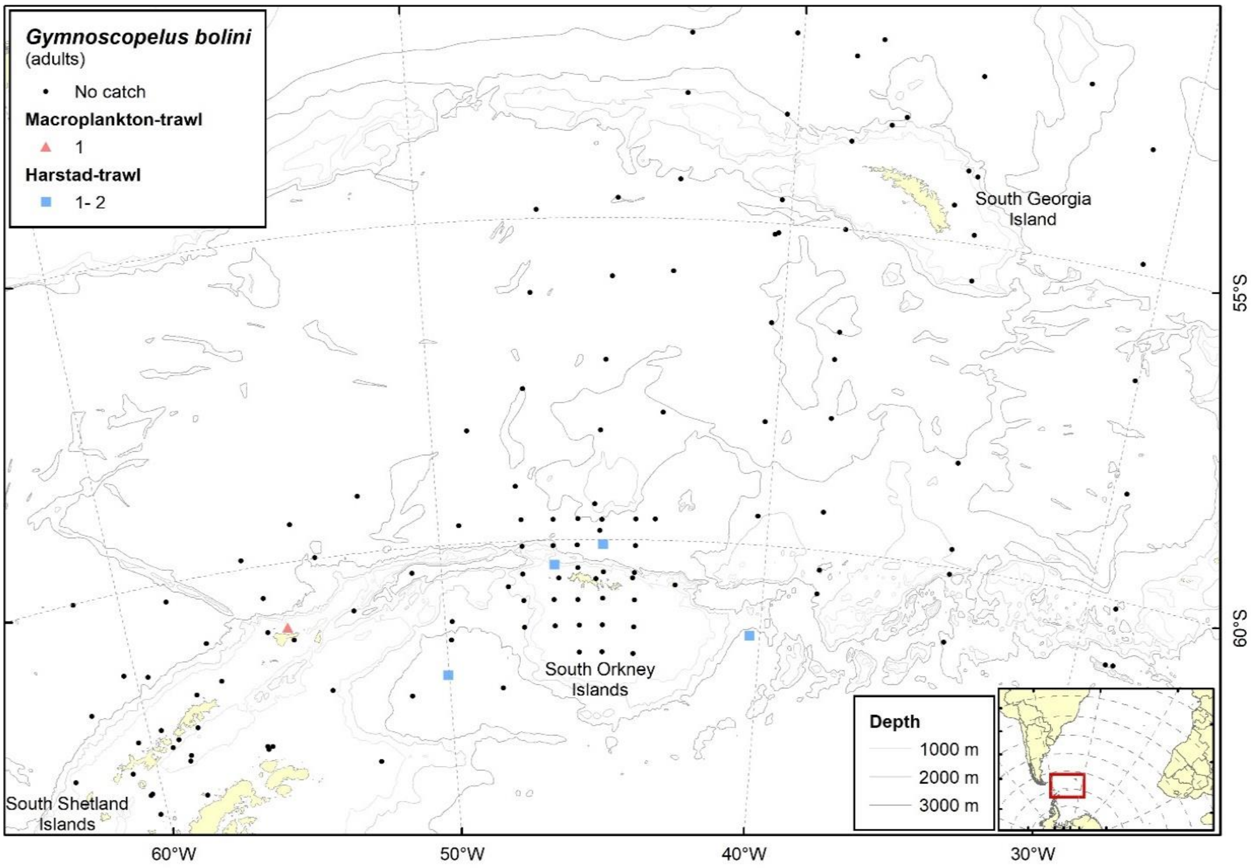

Myctophidae - lanternfishes

Gymnoscopelus braueri (Lönnberg, 1905) - Brauer's lanternfish

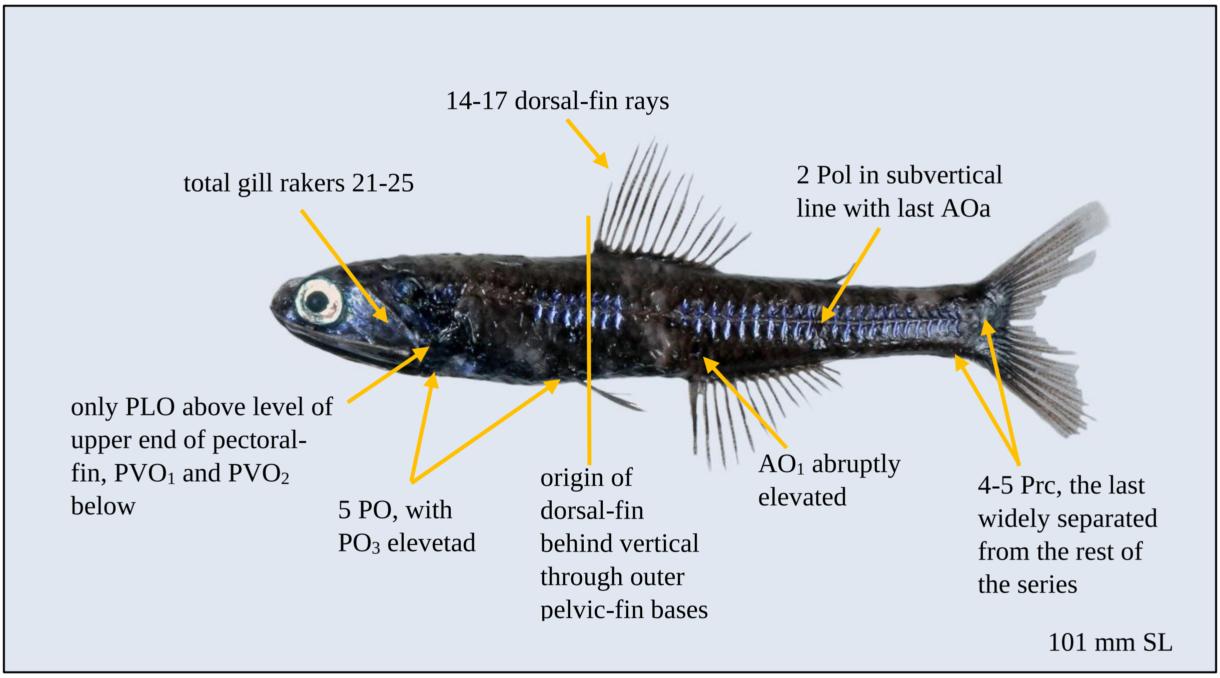

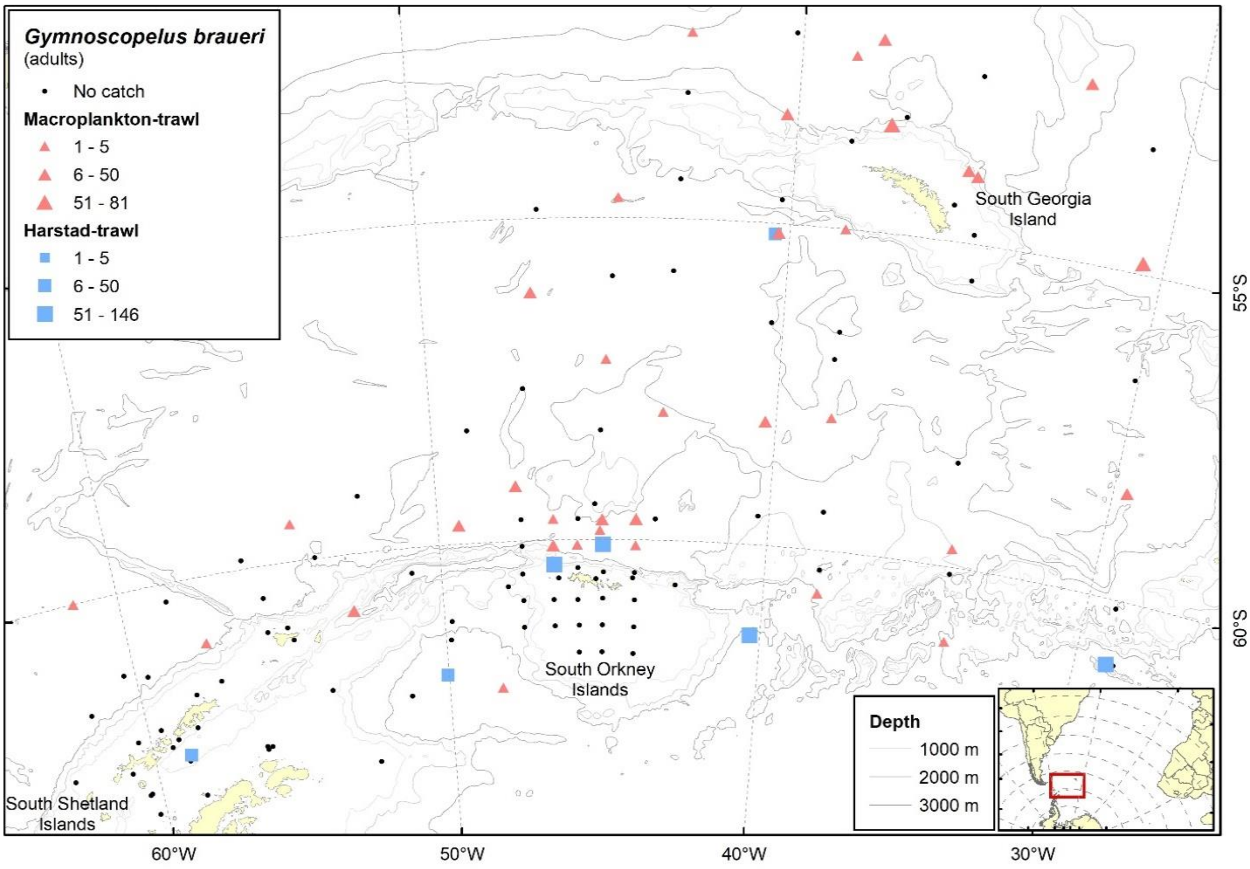

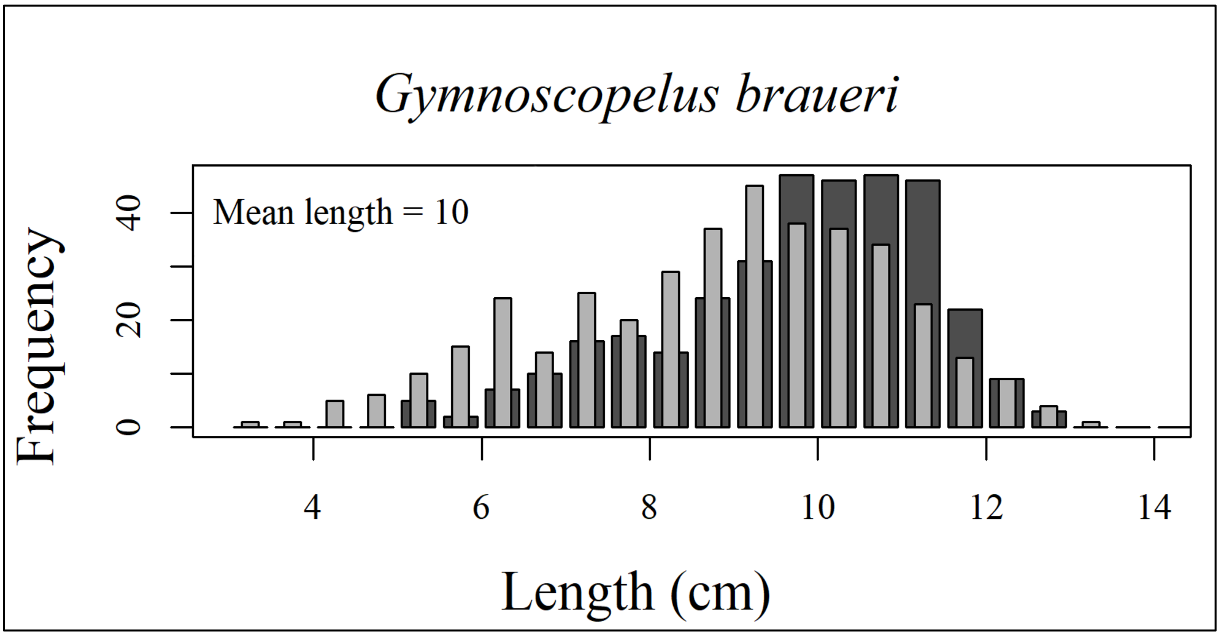

Length distribution of *G. braueri*, light grey: Macroplanktontrawl (N = 391), dark grey: Harstadtrawl (N = 346).

Myctophidae - lanternfishes

Gymnoscopelus fraseri (Fraser-Brunner, 1931) - Fraser's lanternfish

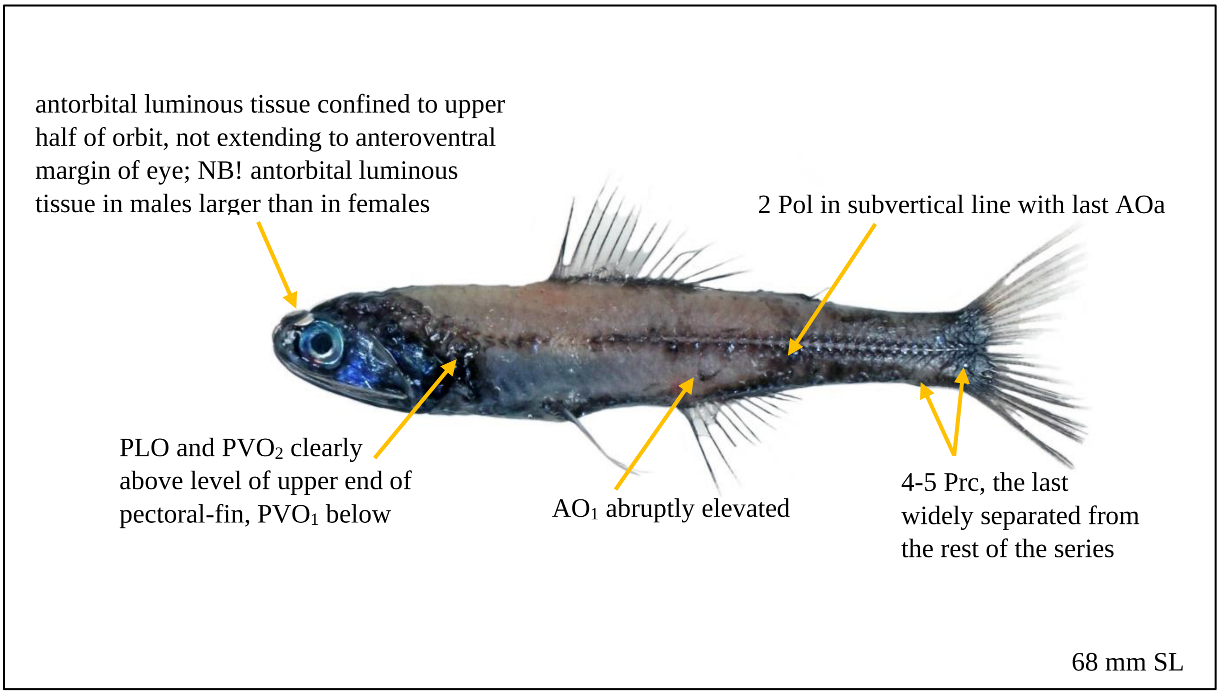

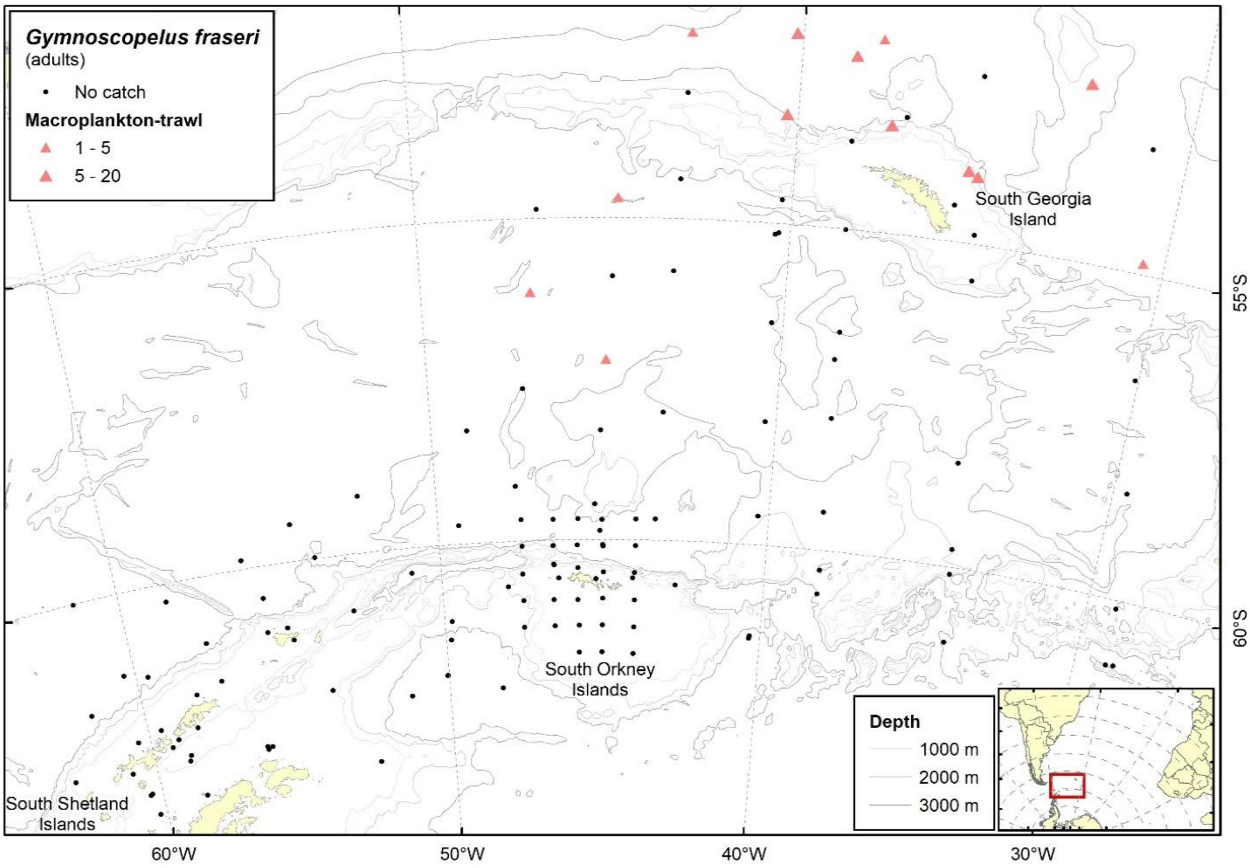
Gymnoscopelus fraseri
(adults)
No catch
Macroplankton-trawl
1 - 5
5 - 20
South Georgia Island
South Orkney Islands
South Shetland Islands
Depth
1000 m
2000 m
3000 m
55°S
60°S
60°W
50°W
40°W
30°W

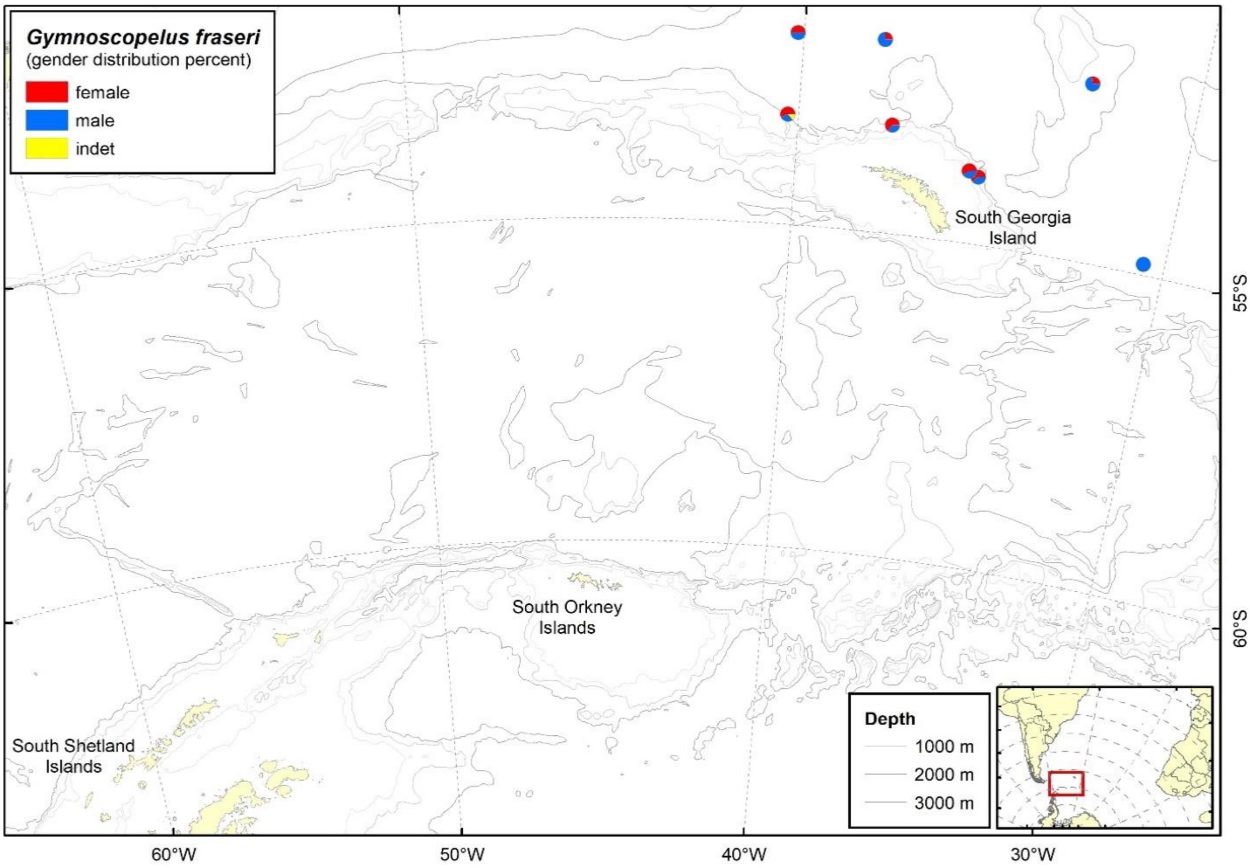
Gymnoscopelus fraseri
(gender distribution percent)
female
male
indet
South Georgia Island
South Orkney Islands
South Shetland Islands
Depth
1000 m
2000 m
3000 m
55°S
60°S
60°W
50°W
40°W
30°W

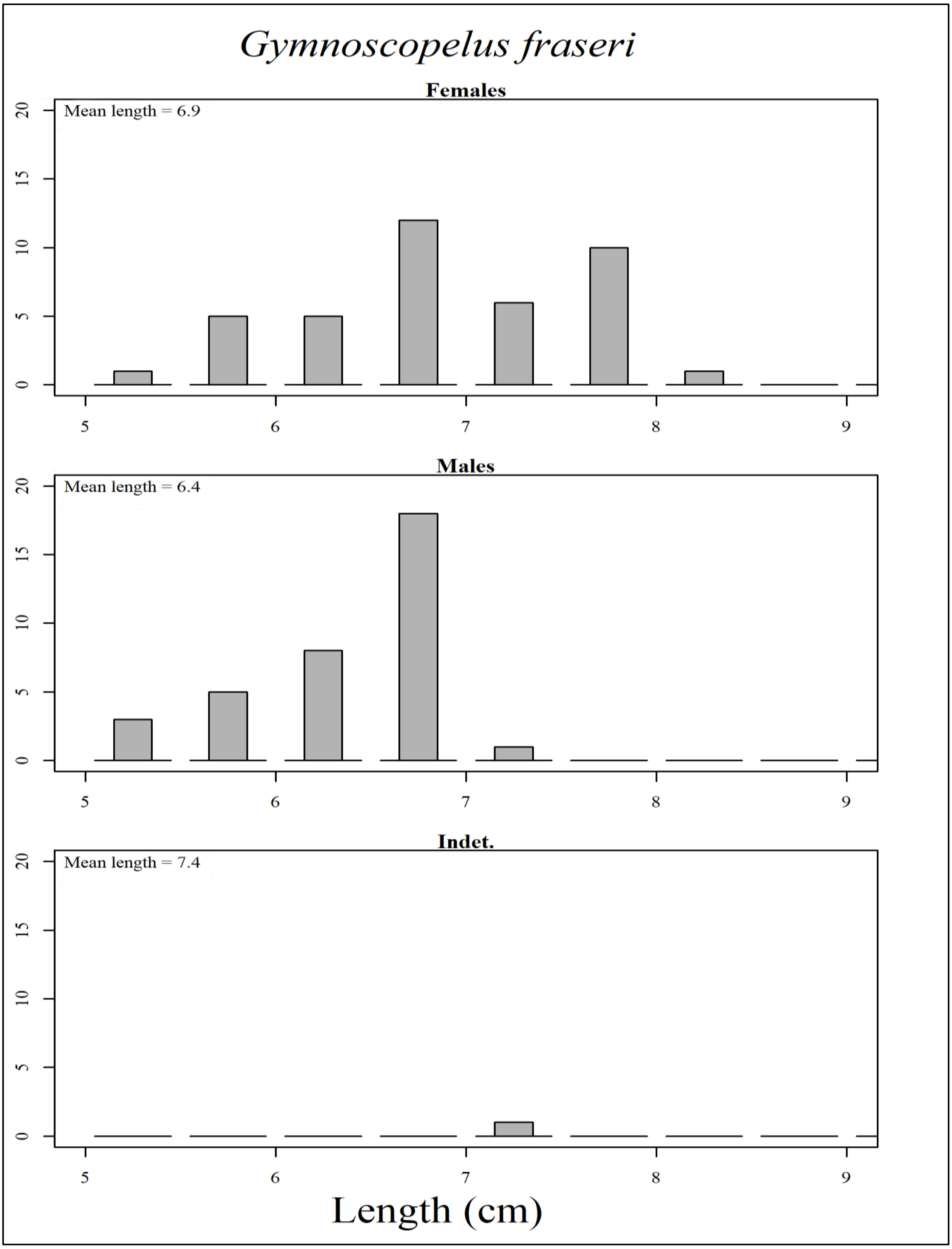

Length distribution of *G. fraseri*, N = 40 (females), 35 (males), 1 (indet.).

Myctophidae - lanternfishes

Gymnoscopelus hintonoides Hulley, 1981 - false-midas lanternfish

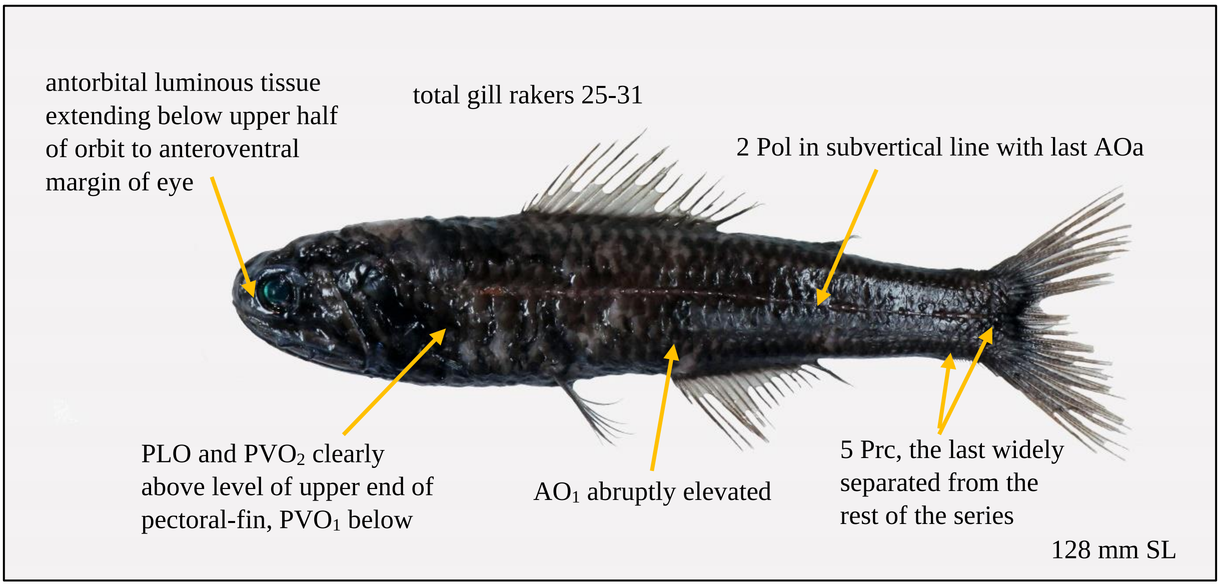

Two specimens were caught at station 4033 and 4047, 128 and 132 mm SL.

Myctophidae - lanternfishes

Gymnoscopelus nicholsi (Gilbert, 1911) - Nichol's lanternfish

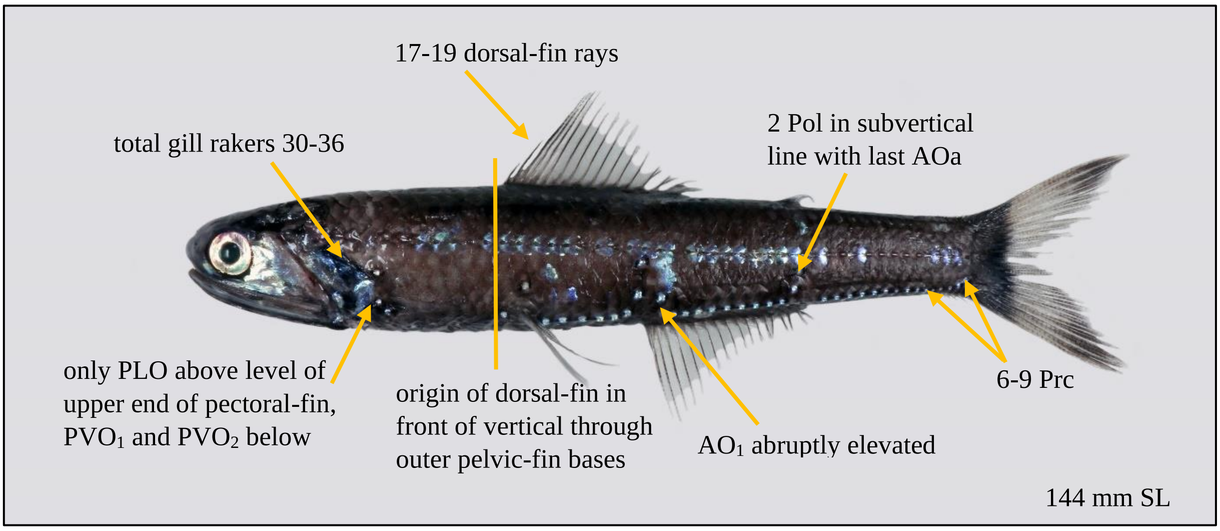

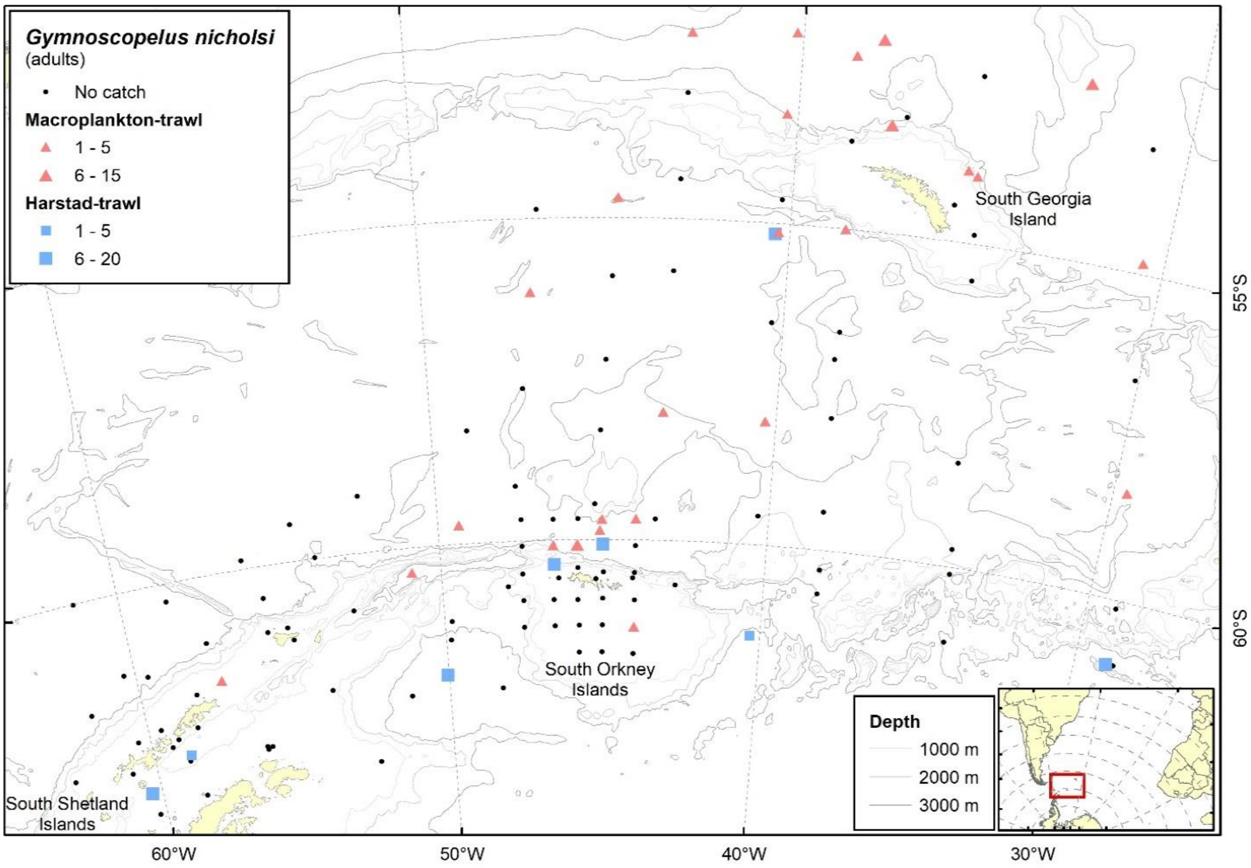

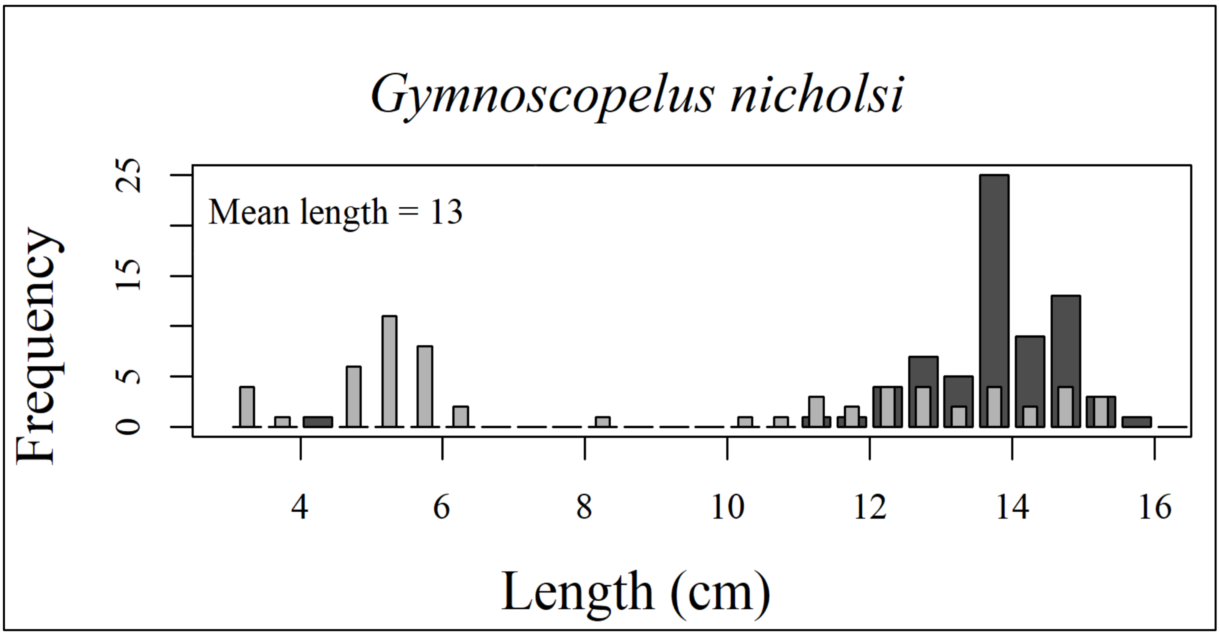

Length distribution of *G. nicholsi*, light grey: Macroplanktontrawl (N = 63), dark grey: Harstadtrawl (N = 70).

Myctophidae - lanternfishes

Gymnoscopelus opisthopterus Fraser-Brunner, 1949

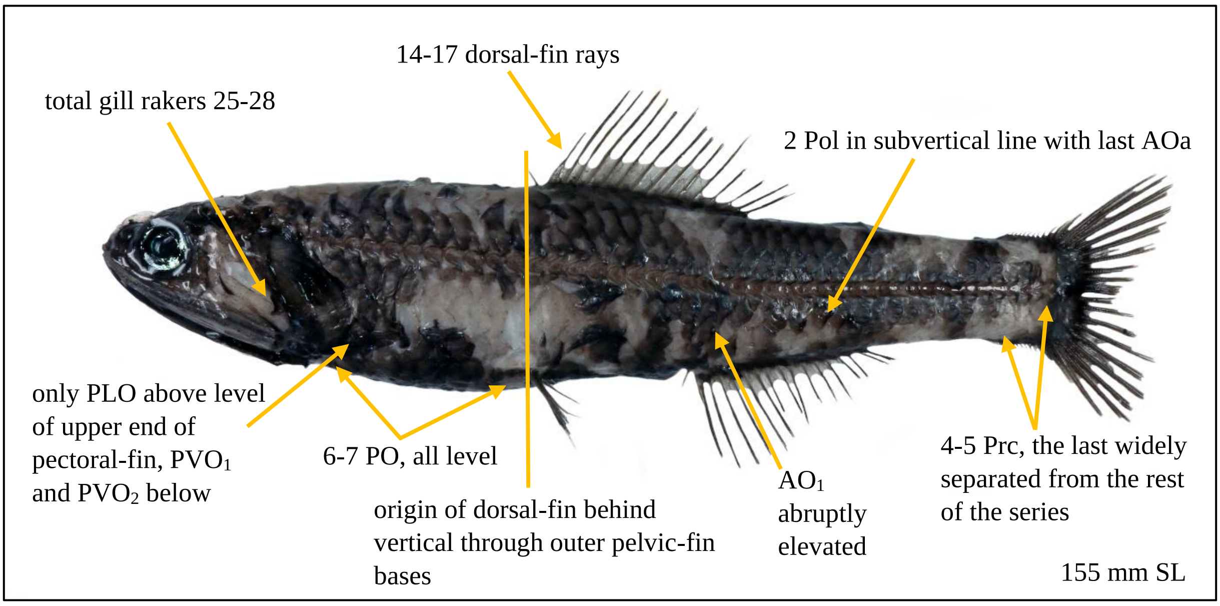

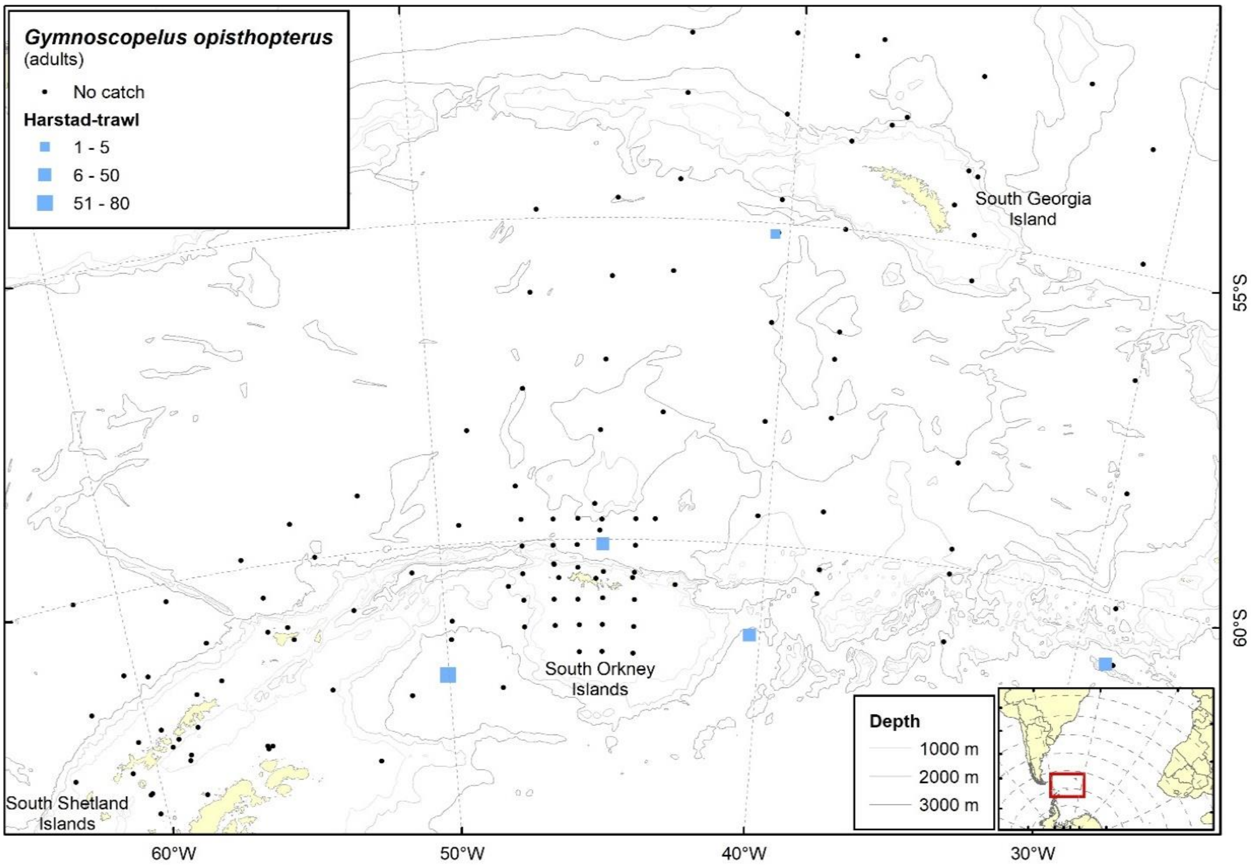

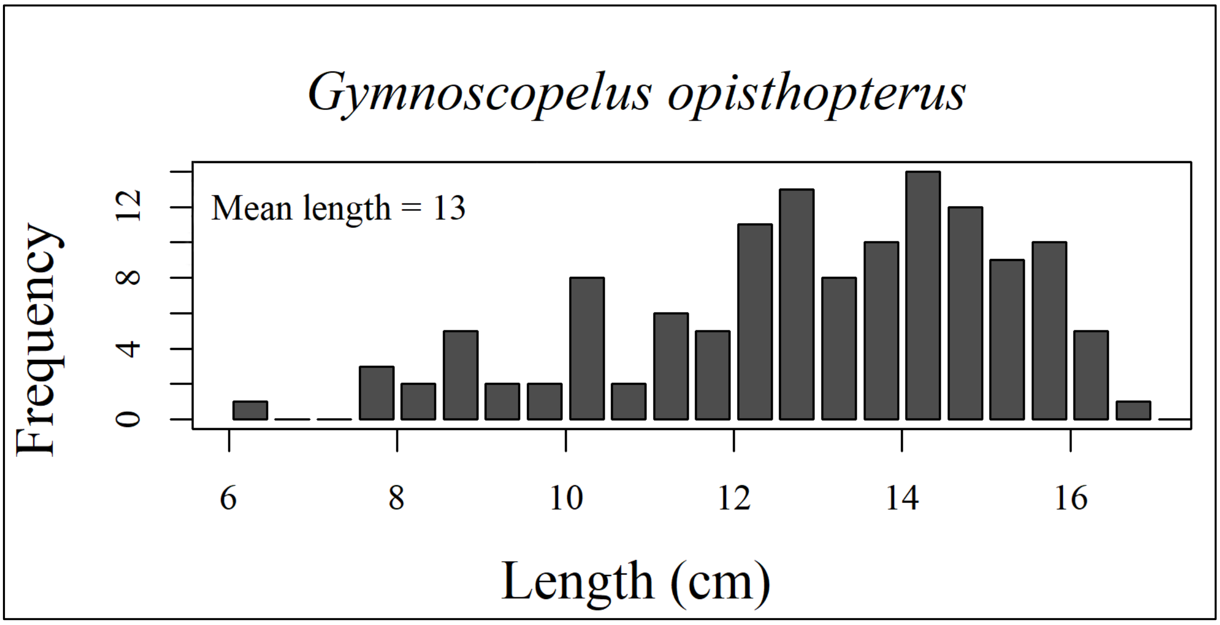

Length distribution of *G. opisthopterus*, N = 129.

Myctophidae - lanternfishes

Gymnoscopelus piabilis (Whitley, 1931) - Southern blacktip lanternfish

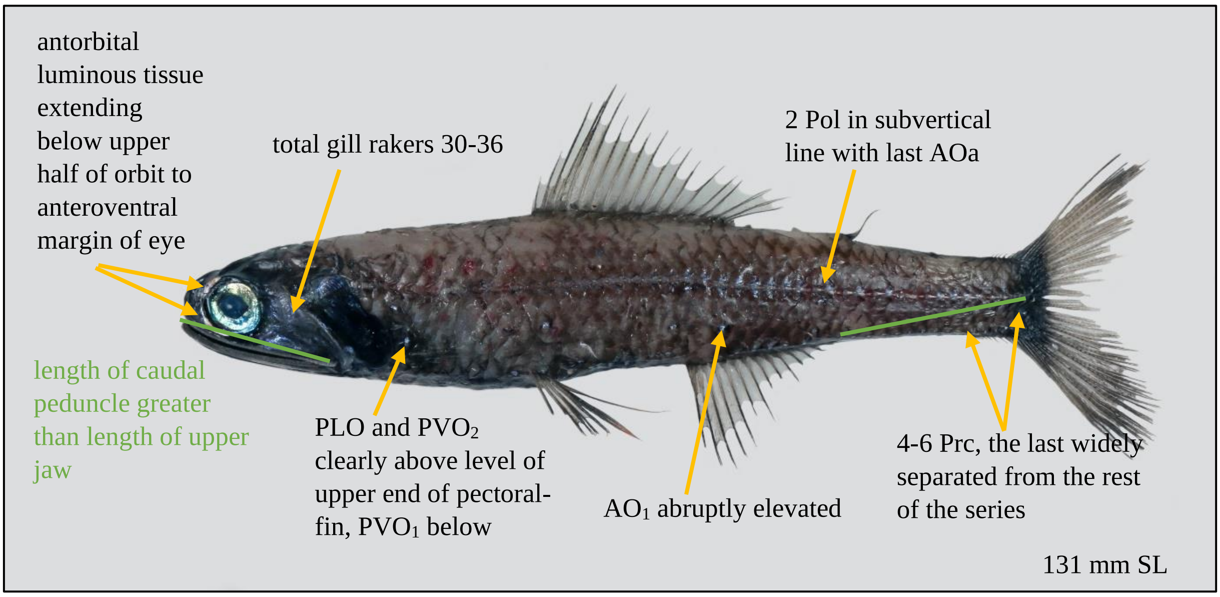

Sixteen specimens were caught, ranging in length from 103 to 147 mm SL.

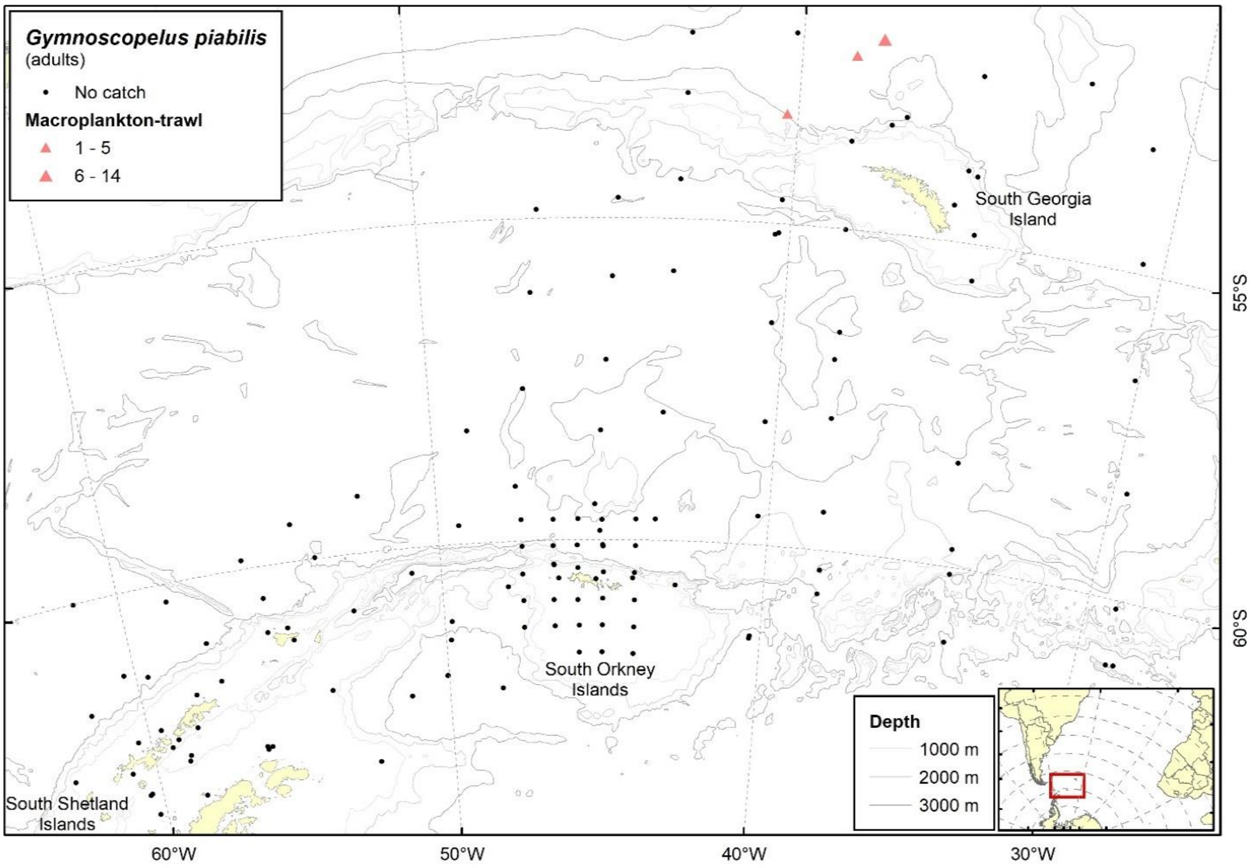

Myctophidae - lanternfishes

Krefftichthys anderssoni (Lönnberg, 1905) - rhombic lanternfish

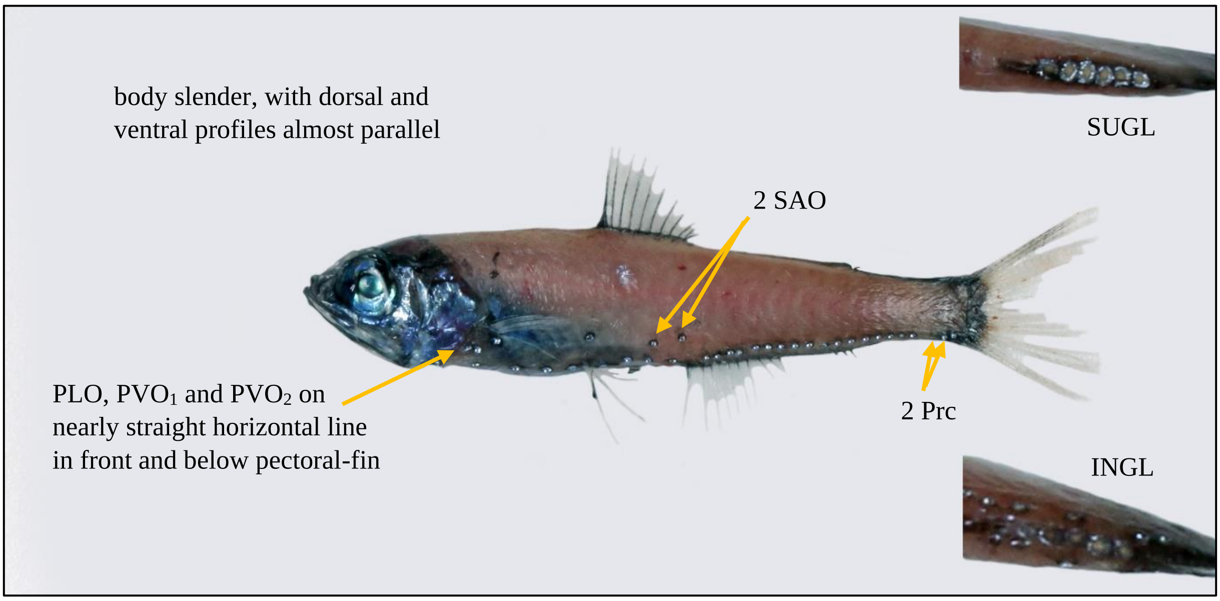

Inset: supracaudal luminous gland (SUGL) of male dorsally, and infracaudal luminous gland (INGL) of female ventrally on caudal peduncle.

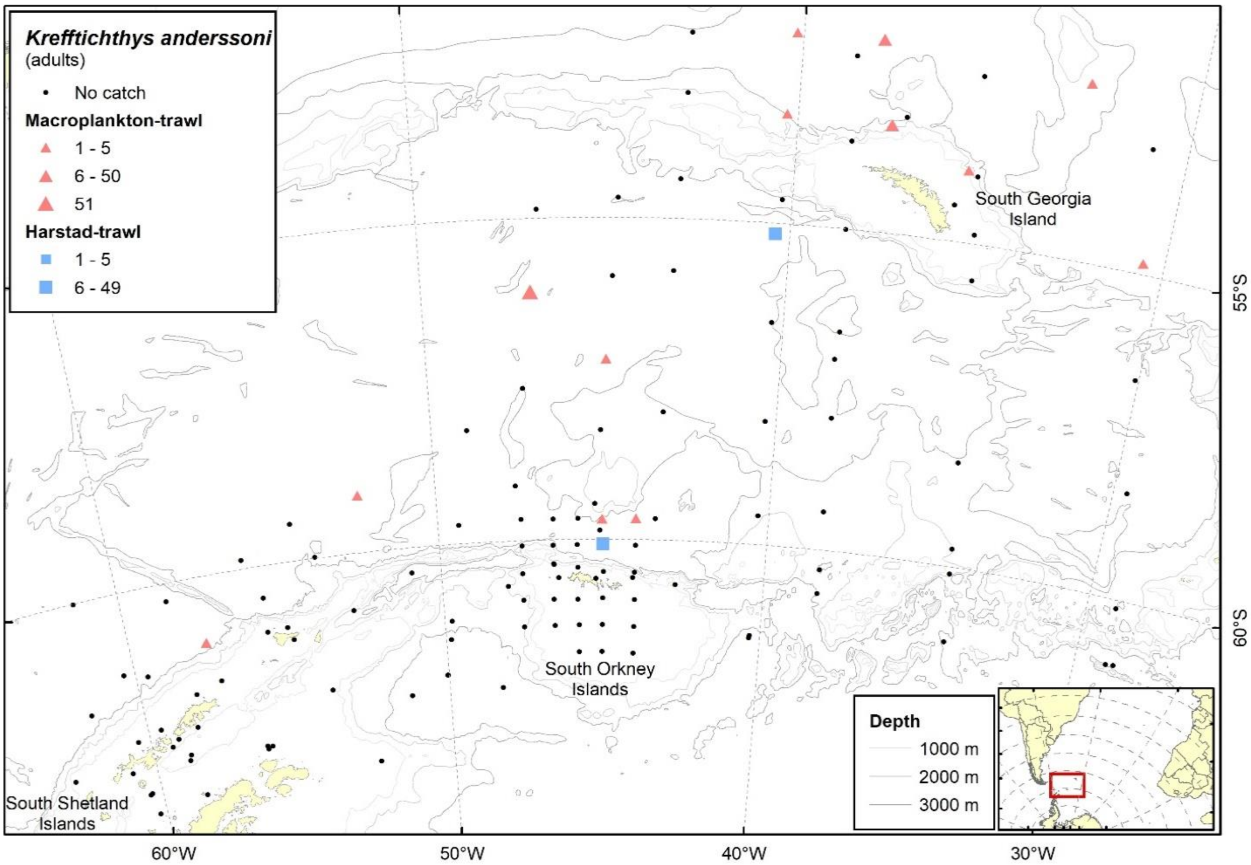
Krefftichthys anderssoni
(adults)
No catch
Macroplankton-trawl
1 - 5
6 - 50
51
Harstad-trawl
1 - 5
6 - 49
South Georgia Island
South Orkney Islands
South Shetland Islands
Depth
1000 m
2000 m
3000 m
55°S
60°S
60°W
50°W
40°W
30°W

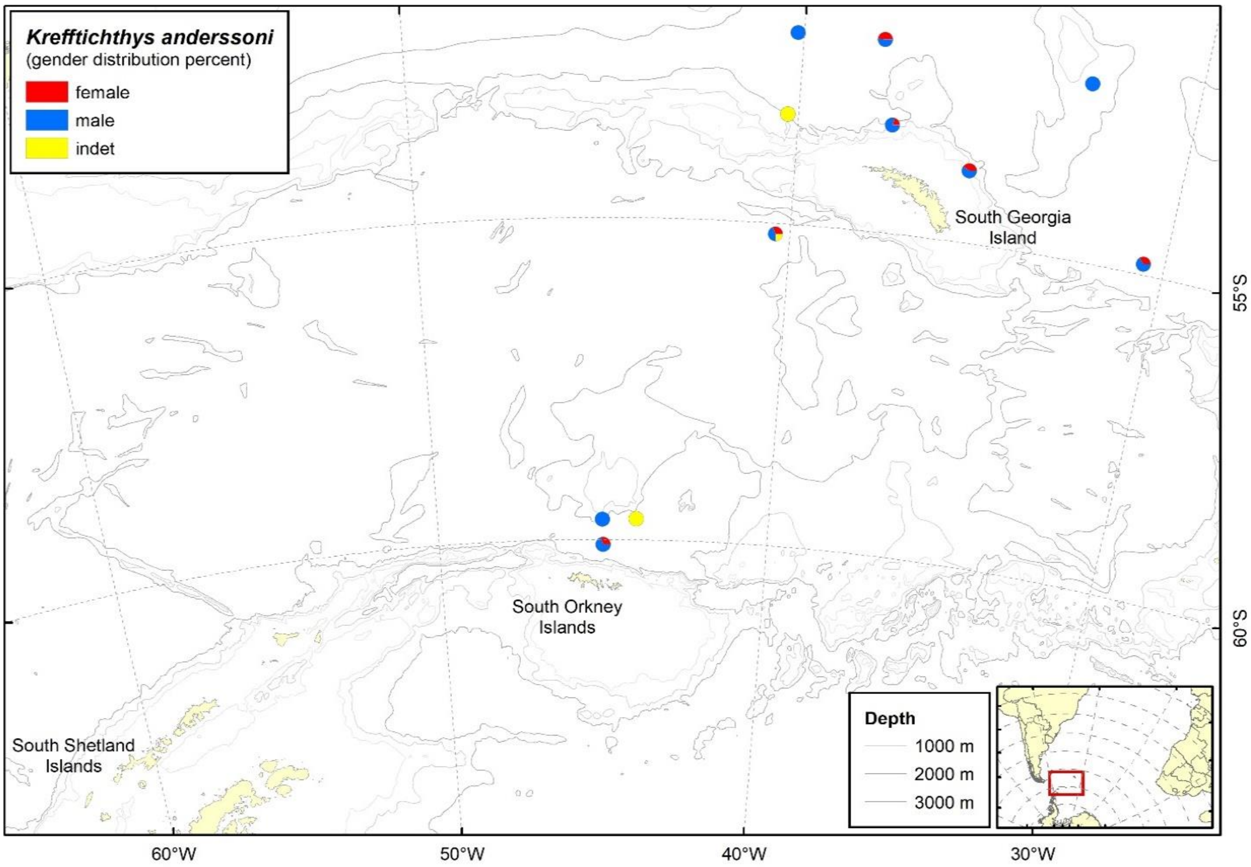
Krefftichthys anderssoni
(gender distribution percent)
female
male
indet
South Georgia Island
South Orkney Islands
South Shetland Islands
Depth
1000 m
2000 m
3000 m
55°S
60°S
60°W
50°W
40°W
30°W

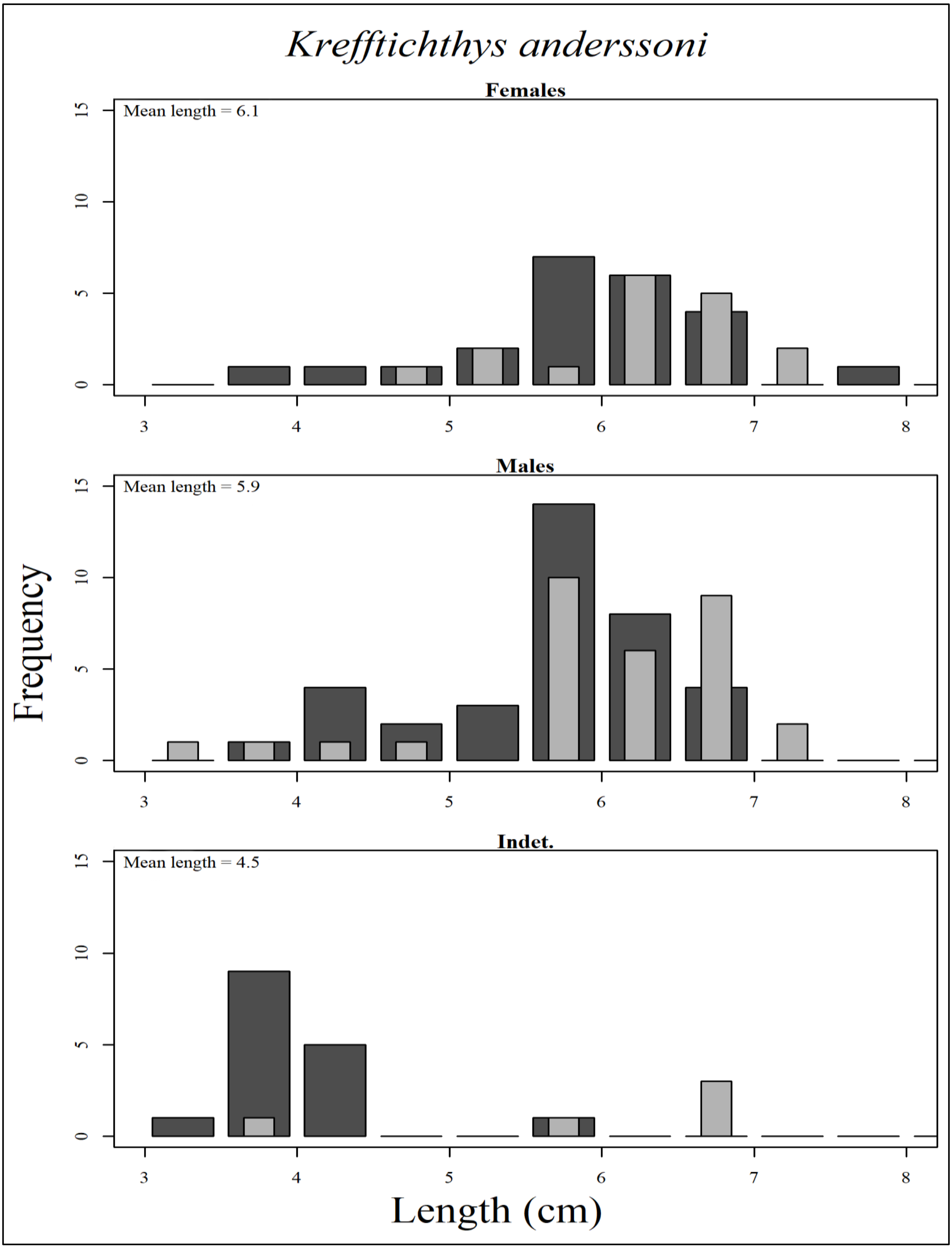

Length distribution of *K. anderssoni*, light grey: Macroplanktontrawl, dark grey: Harstadtrawl, N = 17+23 (females), 31+36 (males), 5+16 (indet.).

Myctophidae - lanternfishes

Lampanyctus achirus (Andriashev, 1962)

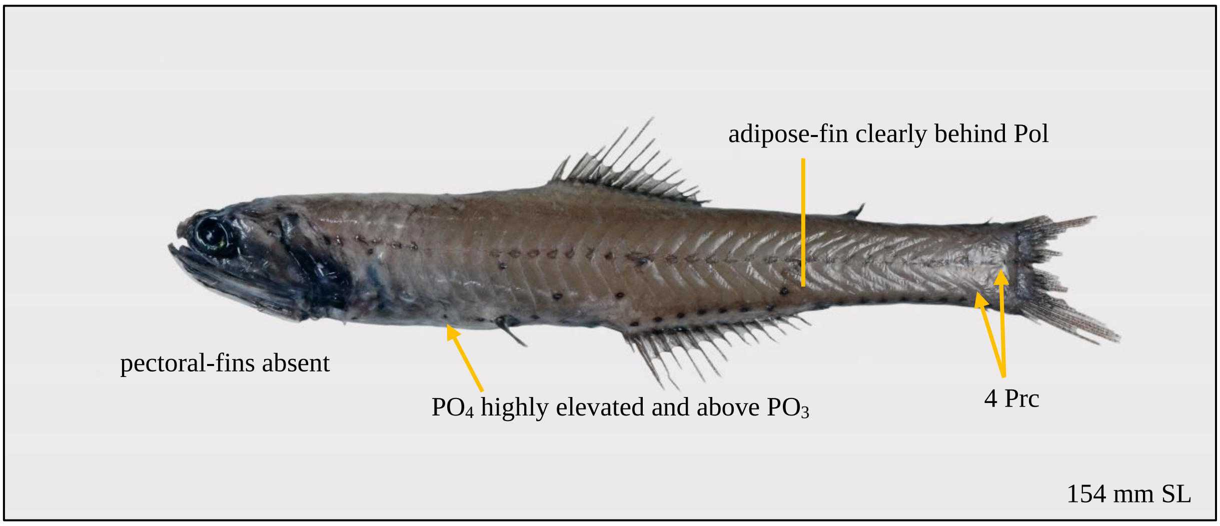

Five specimens were caught, ranging in length from 114 to 154 mm SL.

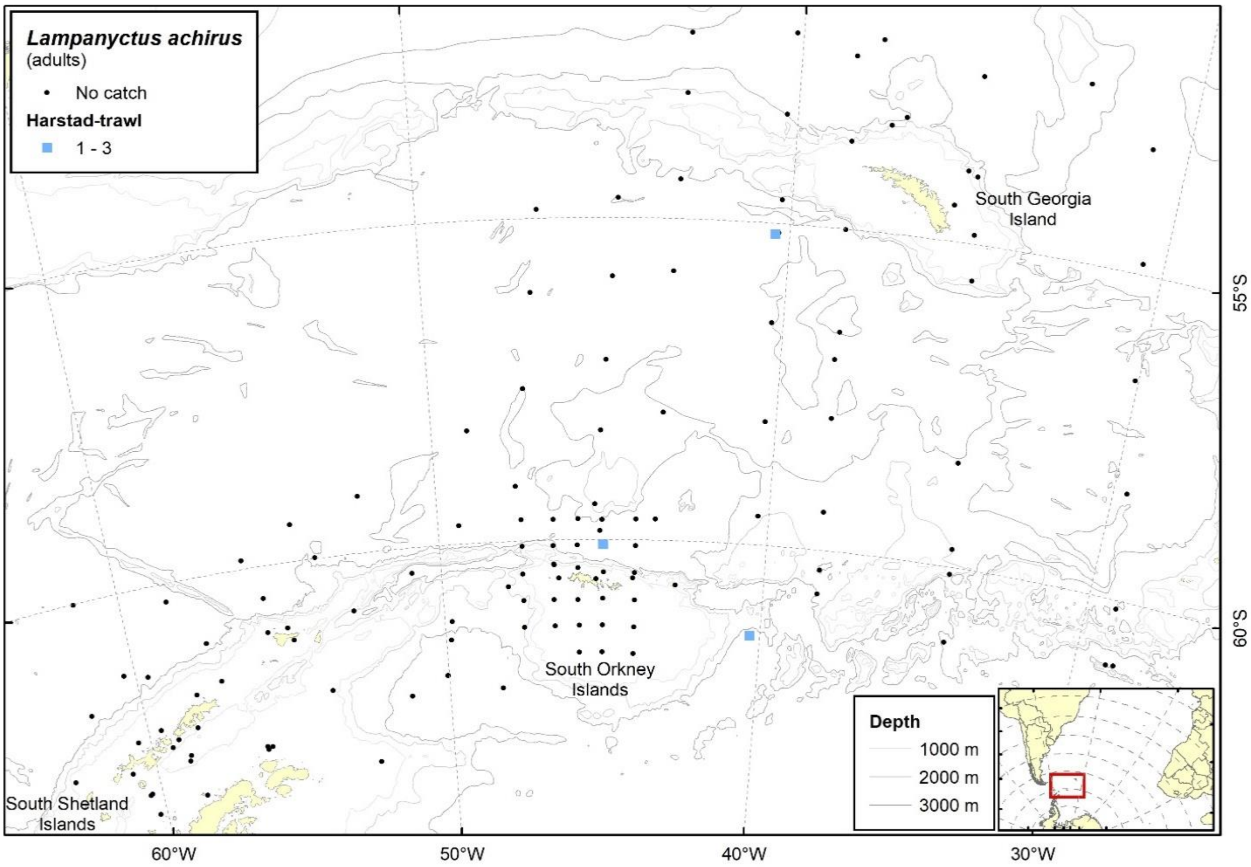

Myctophidae - lanternfishes
Protomyctophum spp. - indet.

Seven specimens caught on FV *Cabo de Hornos* at station 4290 and 4304 could - based on pictures - not be verified to species level.

Myctophidae - lanternfishes
Protomyctophum andriashevi Becker, 1963 - Andriashev's lanternfish

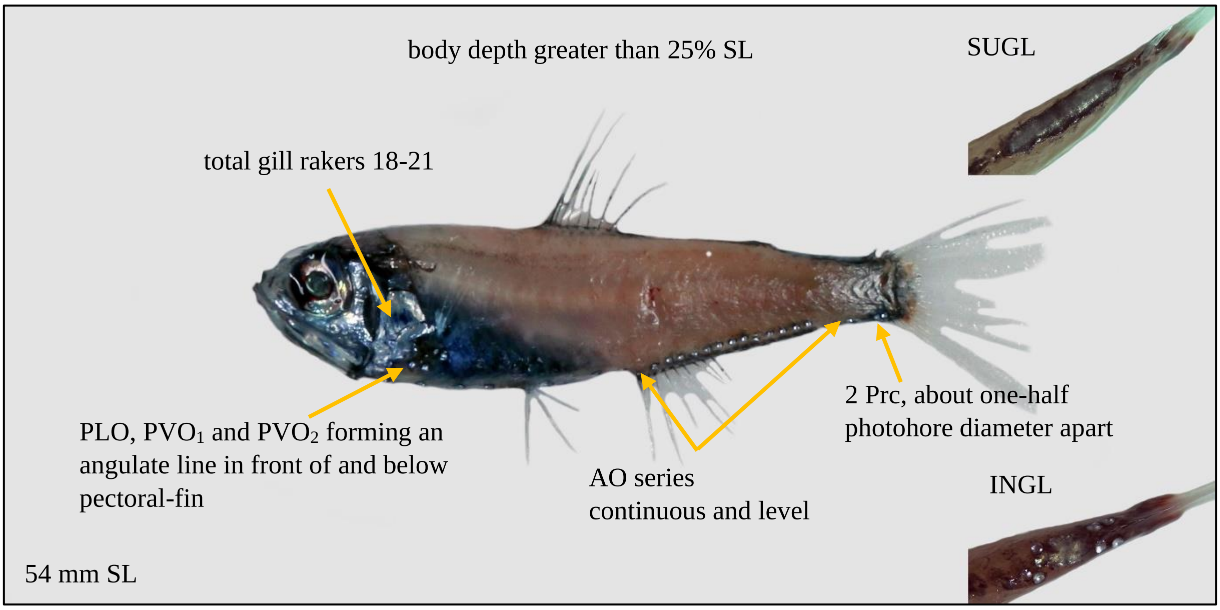

Inset: supracaudal luminous gland (SUGL) of male dorsally, and infracaudal luminous gland (INGL) of female ventrally on caudal peduncle.

Three specimens were caught at station 4050 and 4051, ranging in length from 38 to 54 mm SL.

Myctophidae - lanternfishes

Protomyctophum bolini (Fraser-Brunner, 1949) - Bolin's lanternfish

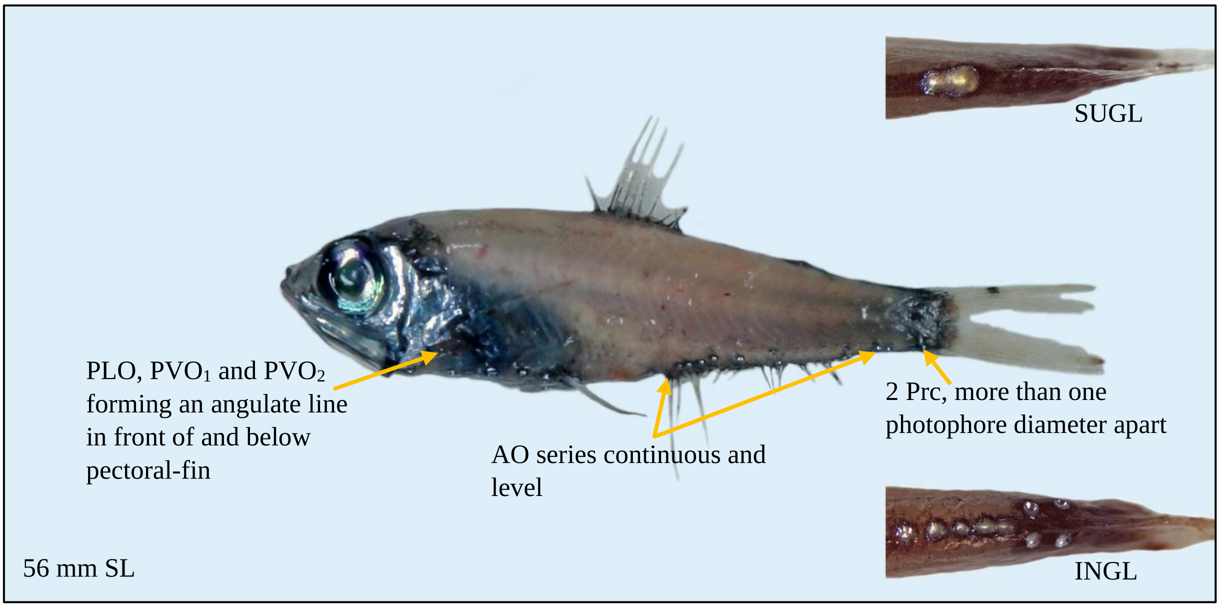

Inset: supracaudal luminous gland (SUGL) of male dorsally, and infracaudal luminous gland (INGL) of female ventrally on caudal peduncle.

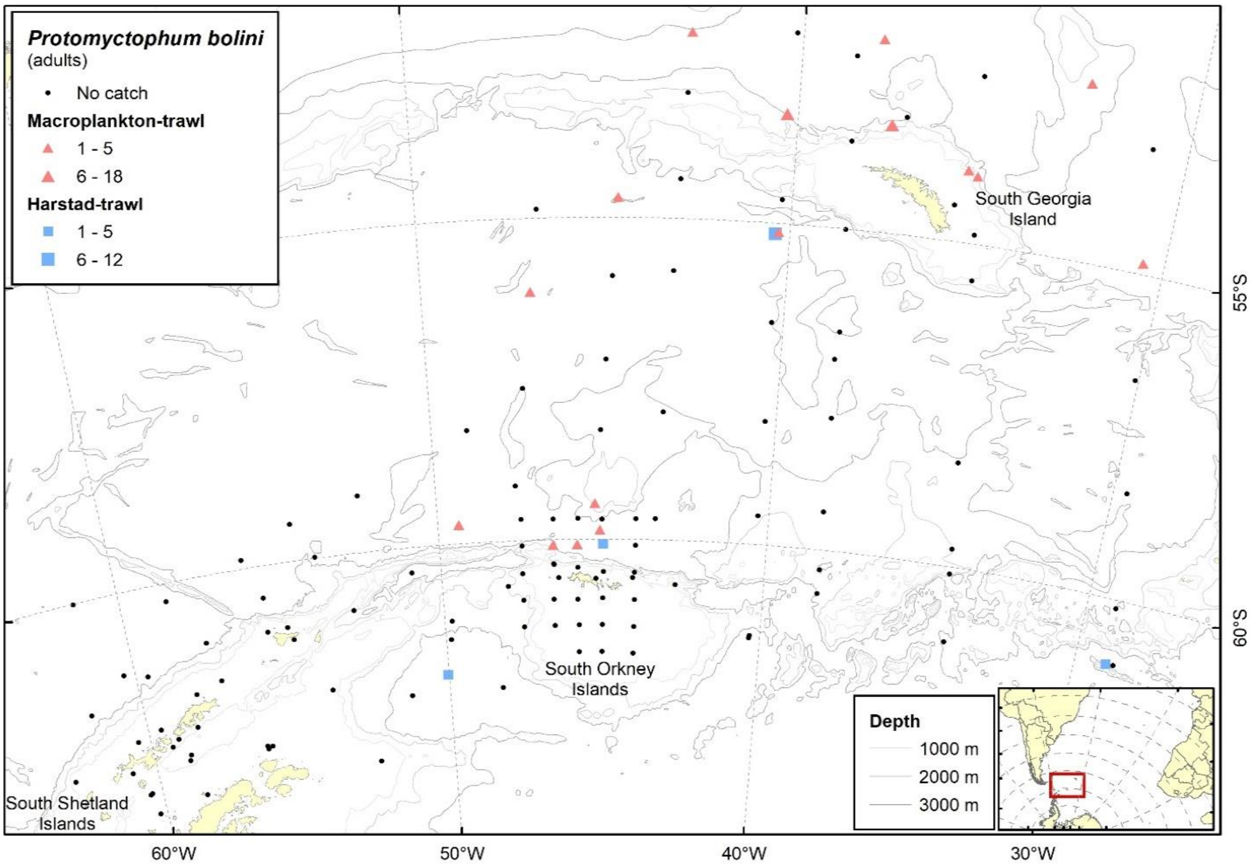

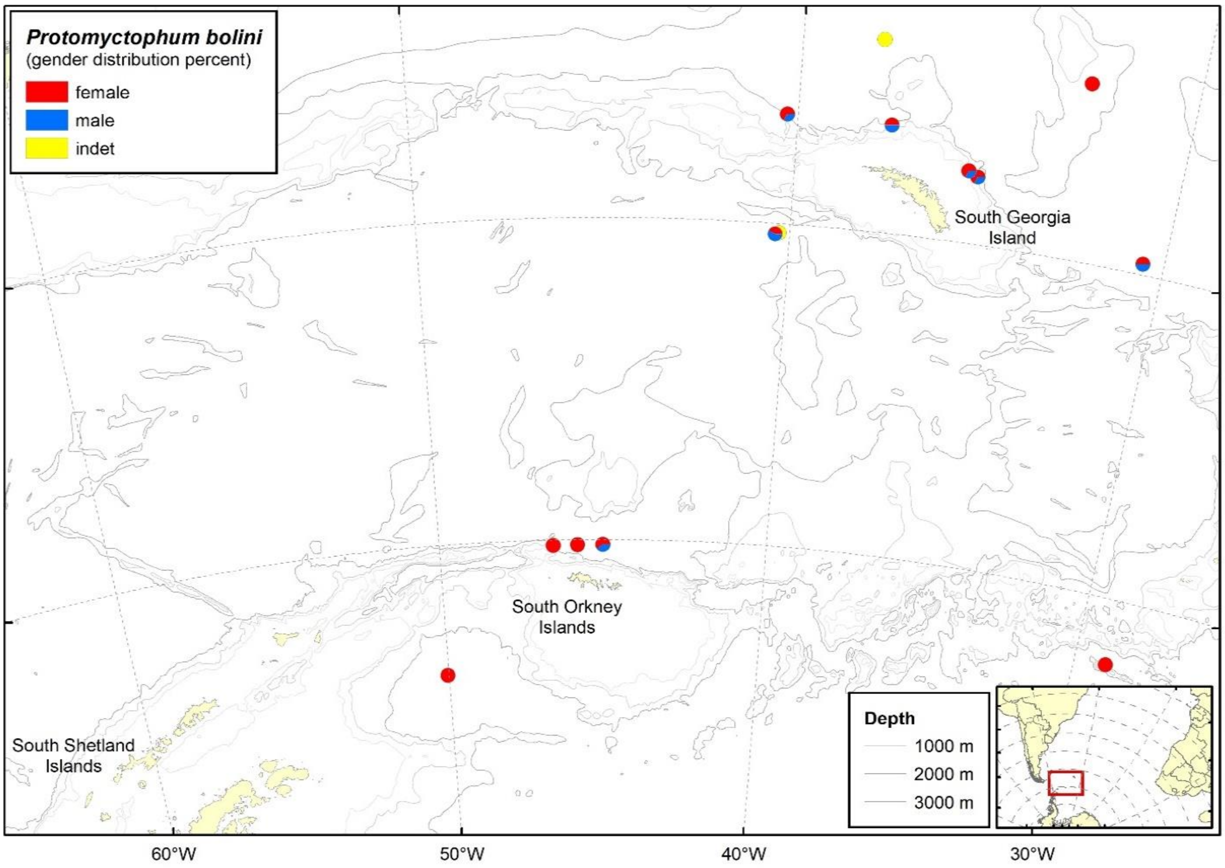

Protomyctophum bolini
(gender distribution percent)
female
male
indet
South Georgia Island
South Orkney Islands
South Shetland Islands
Depth
1000 m
2000 m
3000 m
55°S
60°S
60°W
50°W
40°W
30°W

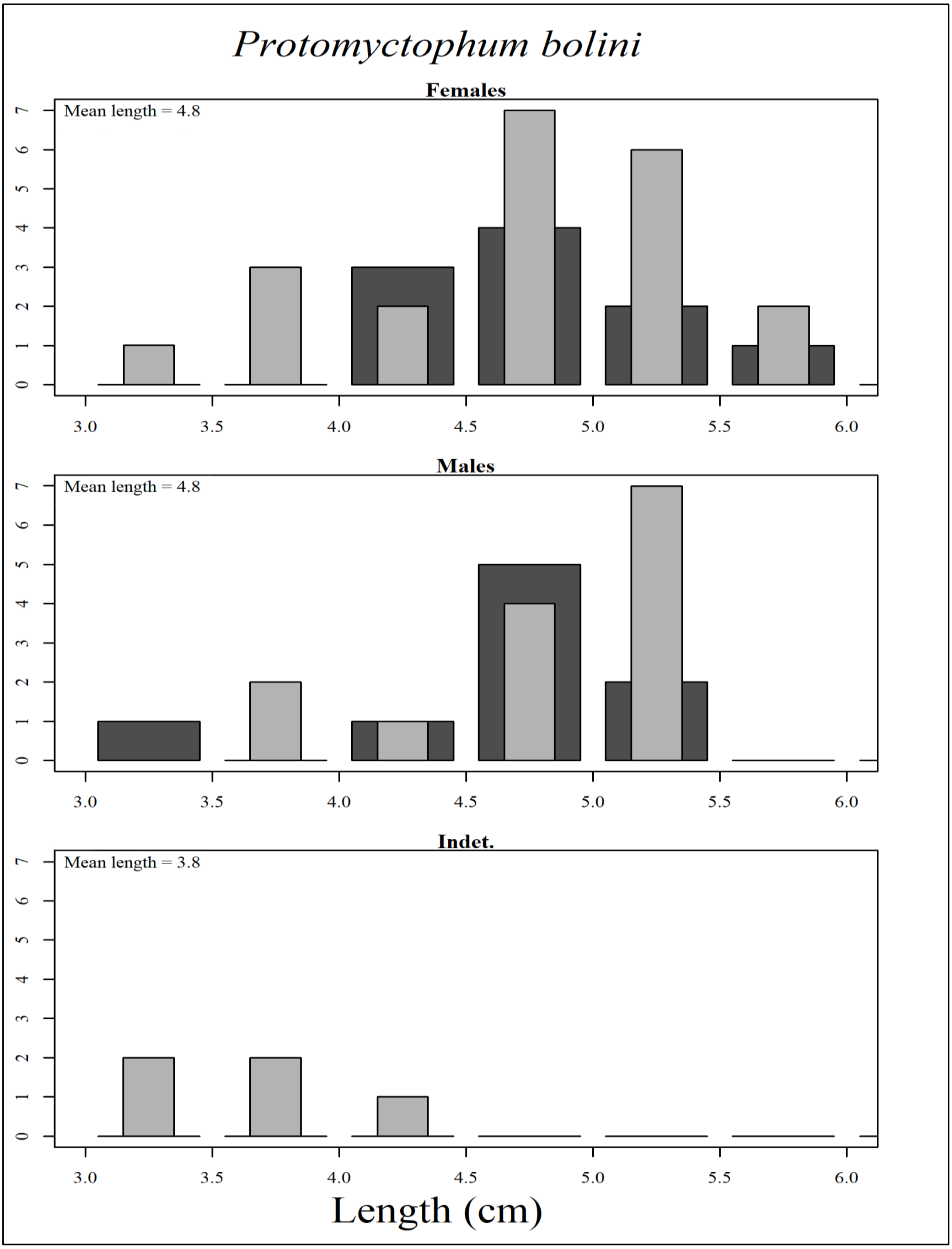

Length distribution of *P. bolini*, light grey: Macroplanktontrawl, dark grey: Harstadtrawl, N = 21+10 (females), 14+9 (males), 5 (indet.).

Myctophidae - lanternfishes

Protomyctophum choriodon Hulley, 1981 - gaptooth lanternfish

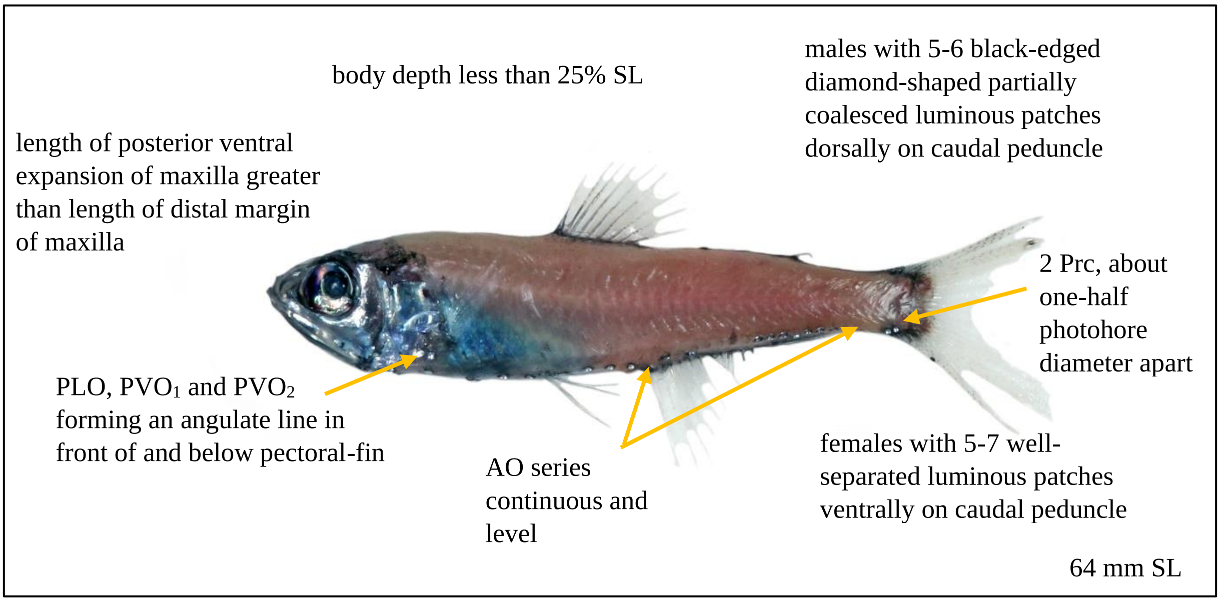

Seven specimens were caught, ranging in length from 35 to 68 mm SL.

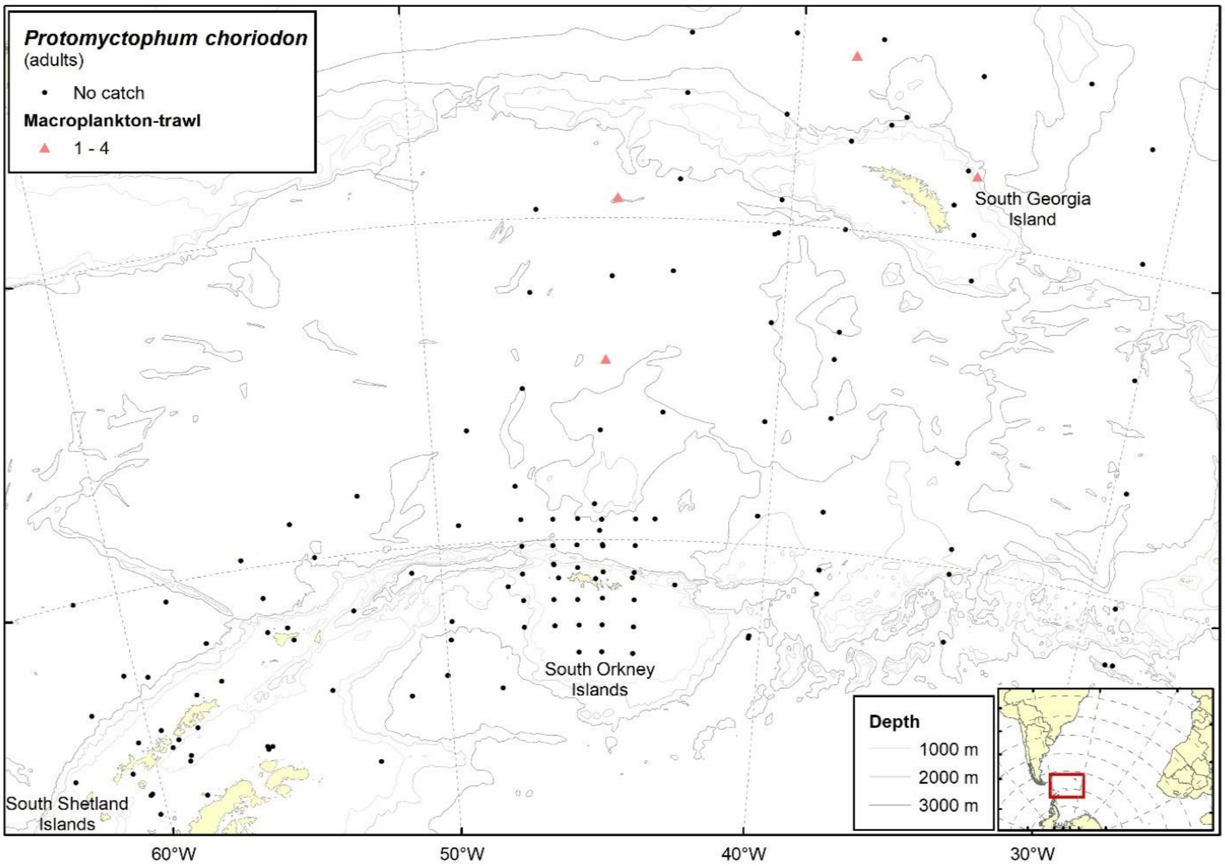

Myctophidae - lanternfishes

Protomyctophum tenisoni (Norman, 1930) - Tenison's lanternfish

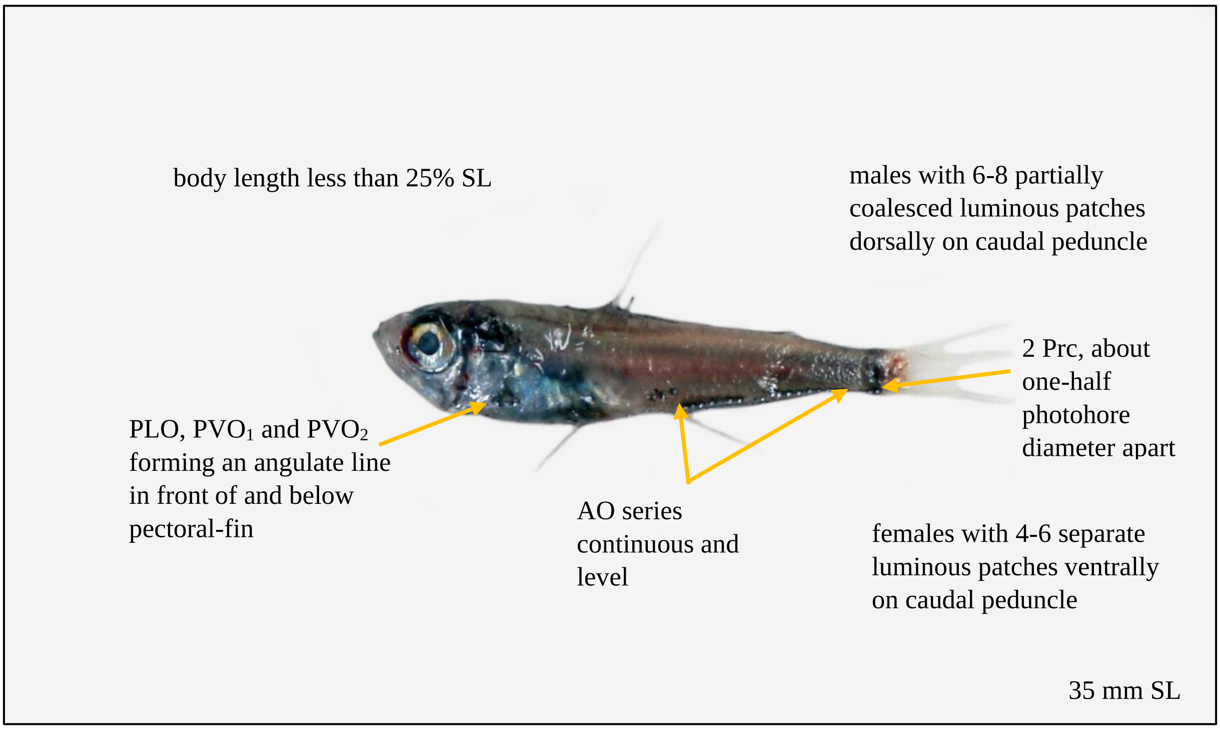

Four specimens were caught at station 4051, ranging in length from 30 to 35 mm SL.

Muraenolepididae - eel cods

Muraenolepis spp. – juvenile

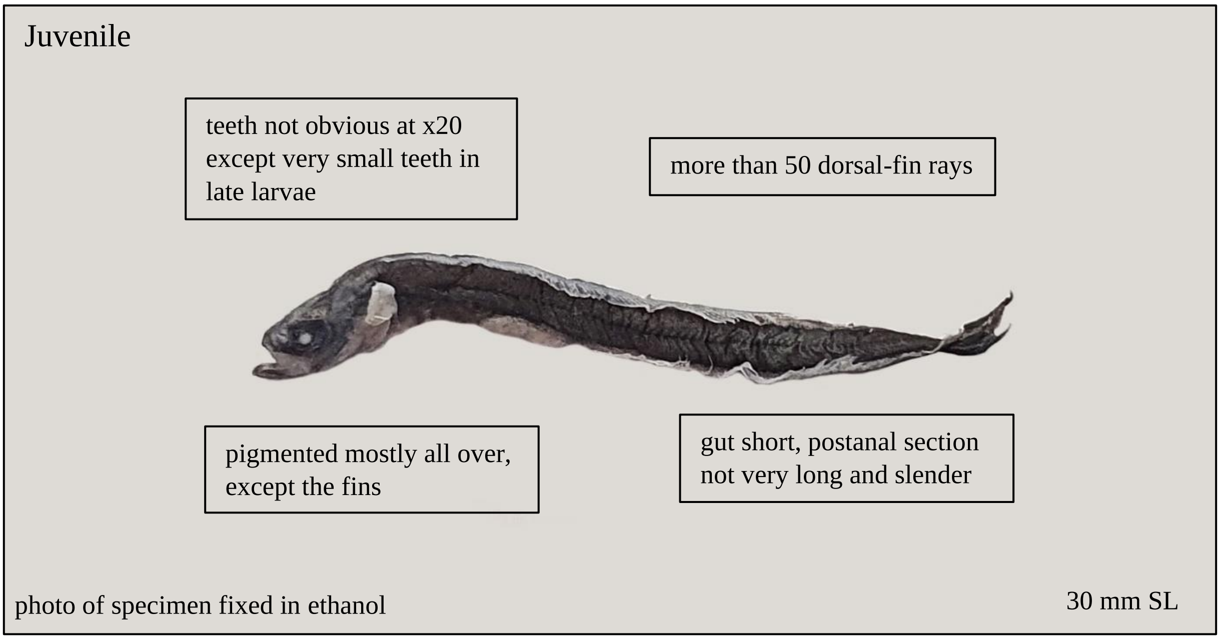

There were 32 specimens length measured, ranging from 19-38 mm TL, mean 26 mm.

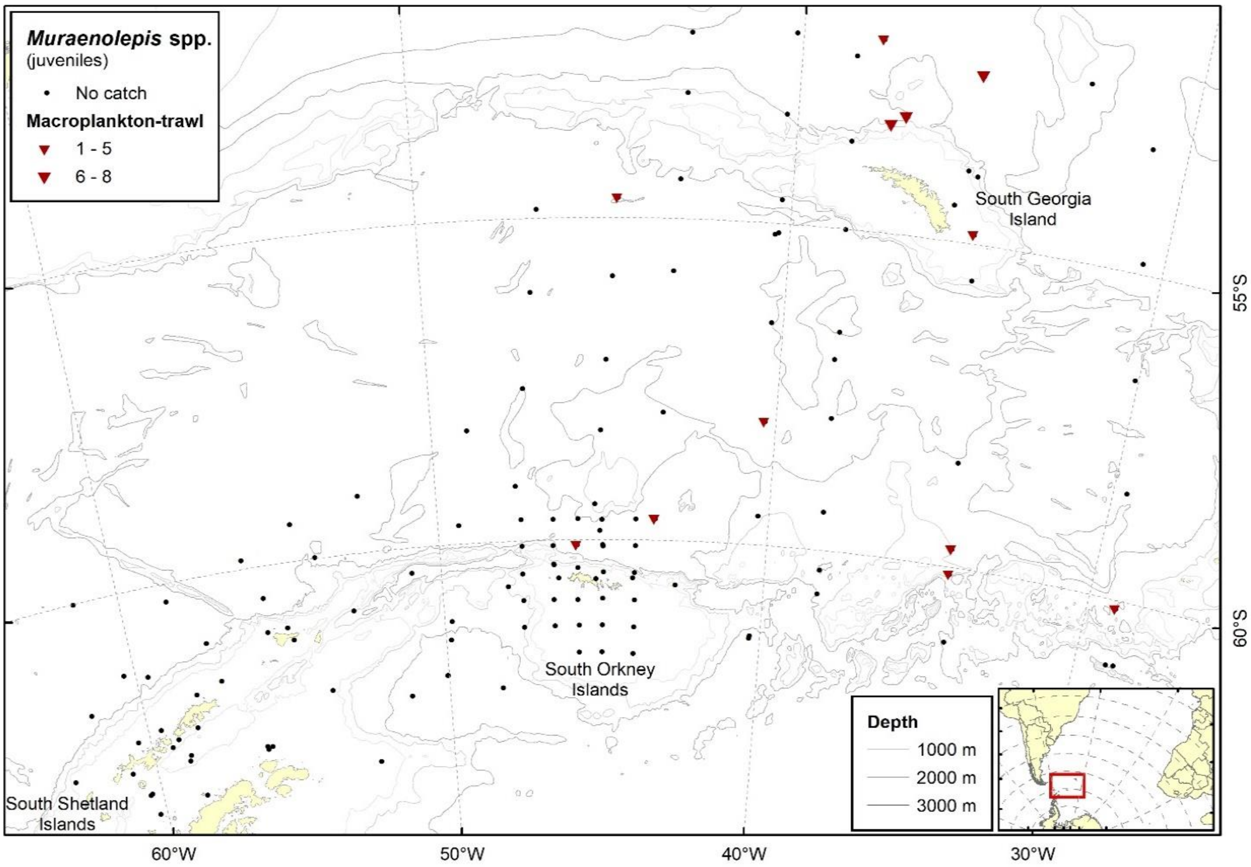

Muraenolepididae - eel cods

Muraenolepis marmorata Günther, 1880 - marbled moray cod

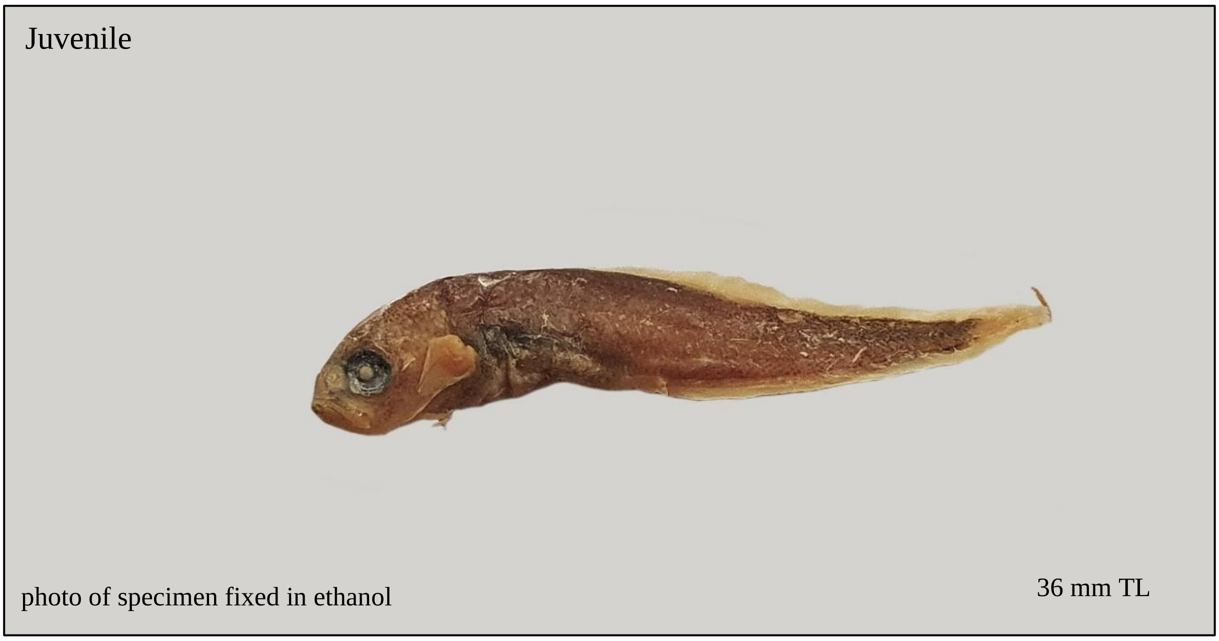

Eight specimens were caught at station 4049, five of these were genetically verified. Length range 27 to 37 mm TL.

Macrouridae - grenadiers or rattails

Cynomacrurus piriei Dollo, 1909 - dogtooth grenadier

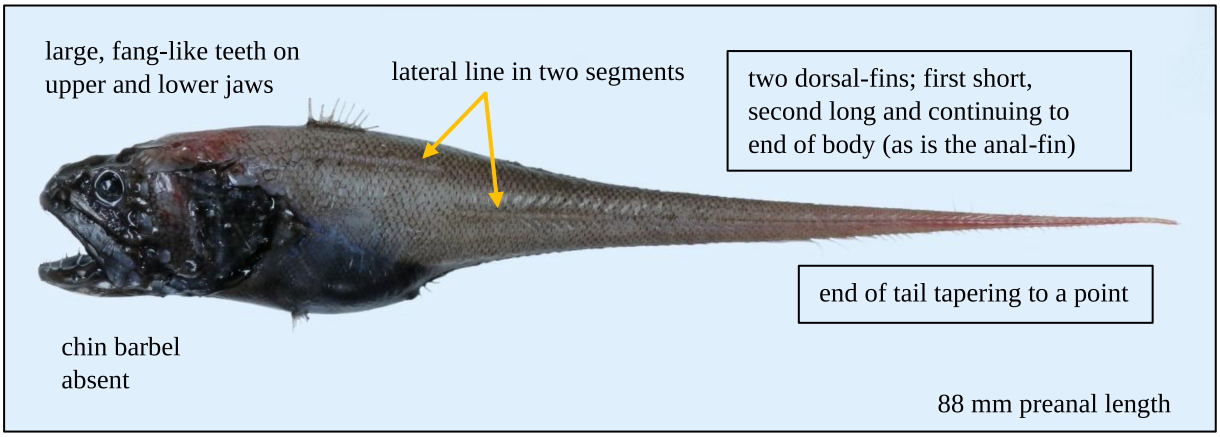

Four specimens were caught, two juveniles 8 and 19 mm, and two adults 68 and 88 mm preanal length.

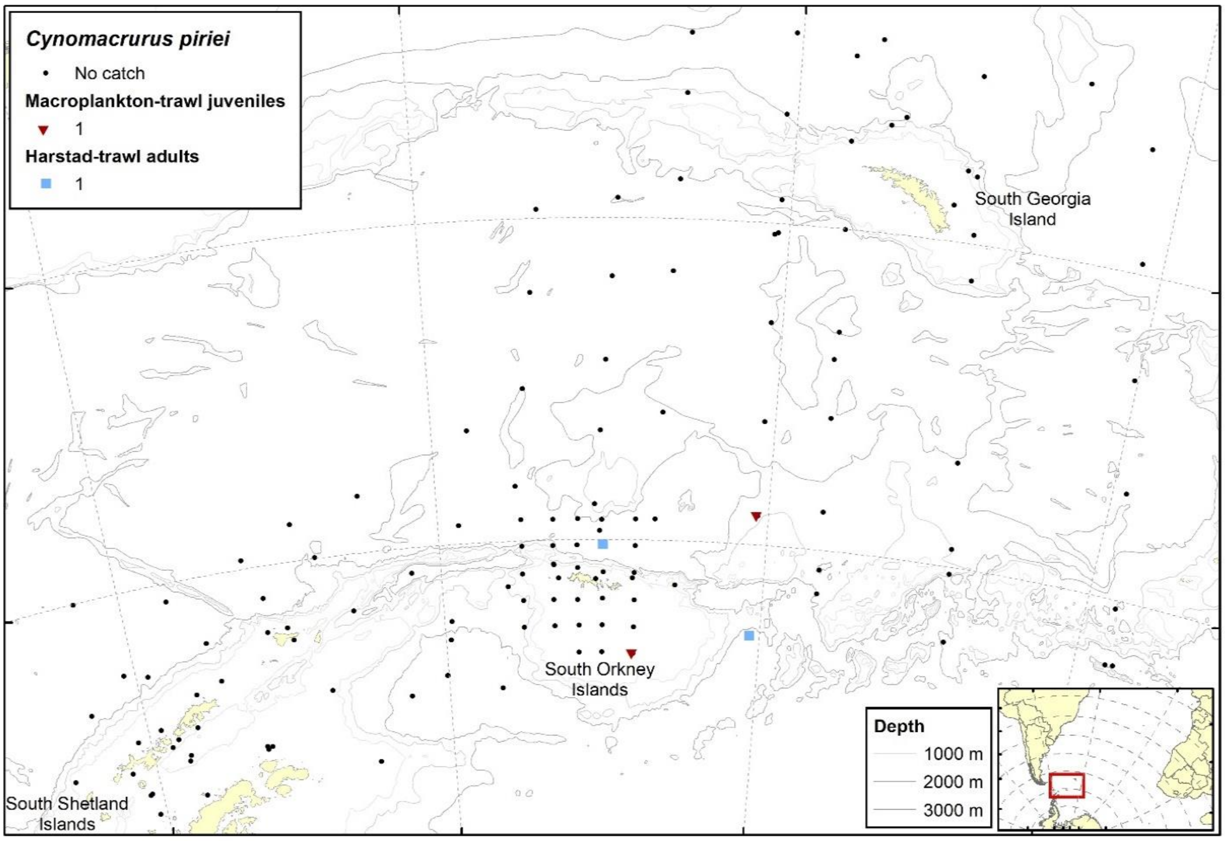

Gempylidae - snake mackerels

Paradiplospinus antarcticus Andriashev, 1960 - Antarctic escolar

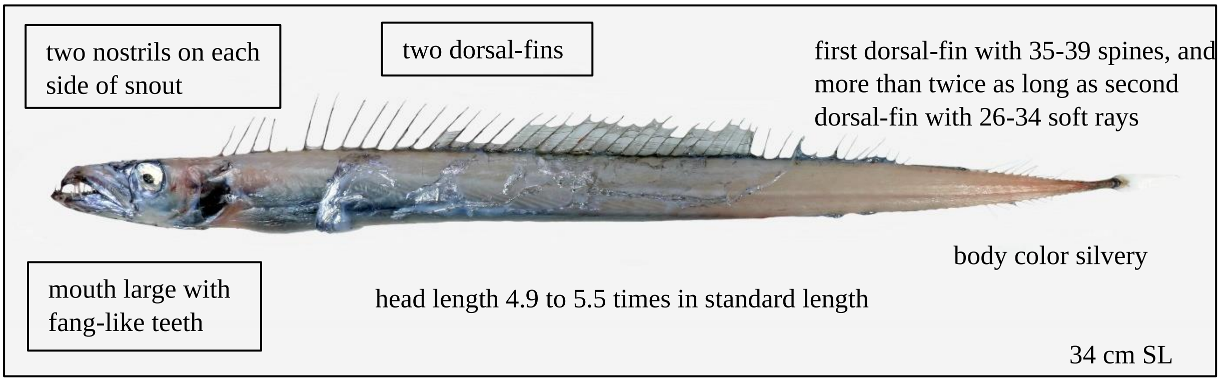

Eighteen specimens were caught, ranging in length from 26 to 43 cm TL.

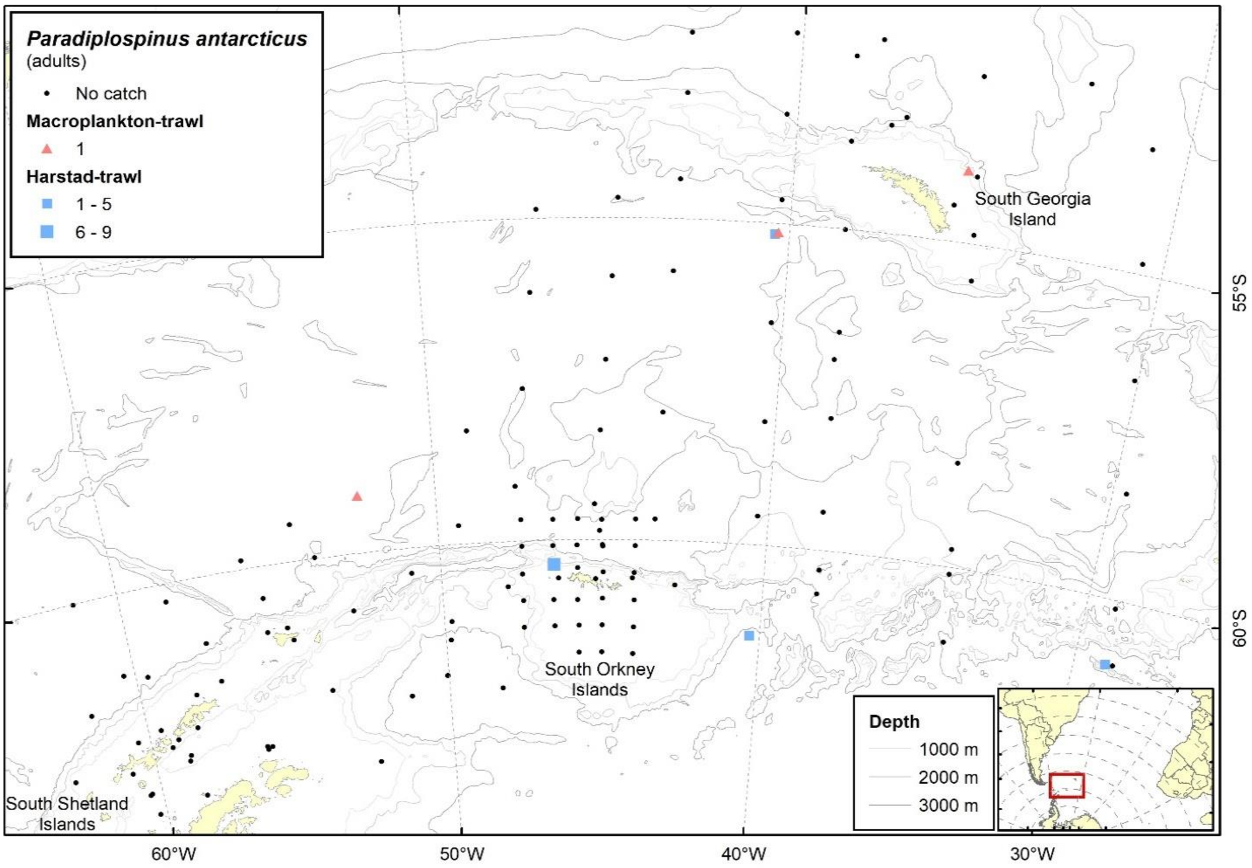

Centrolophidae - medusafishes

Pseudoicichthys australis Haedrich, 1966 - southern driftfish

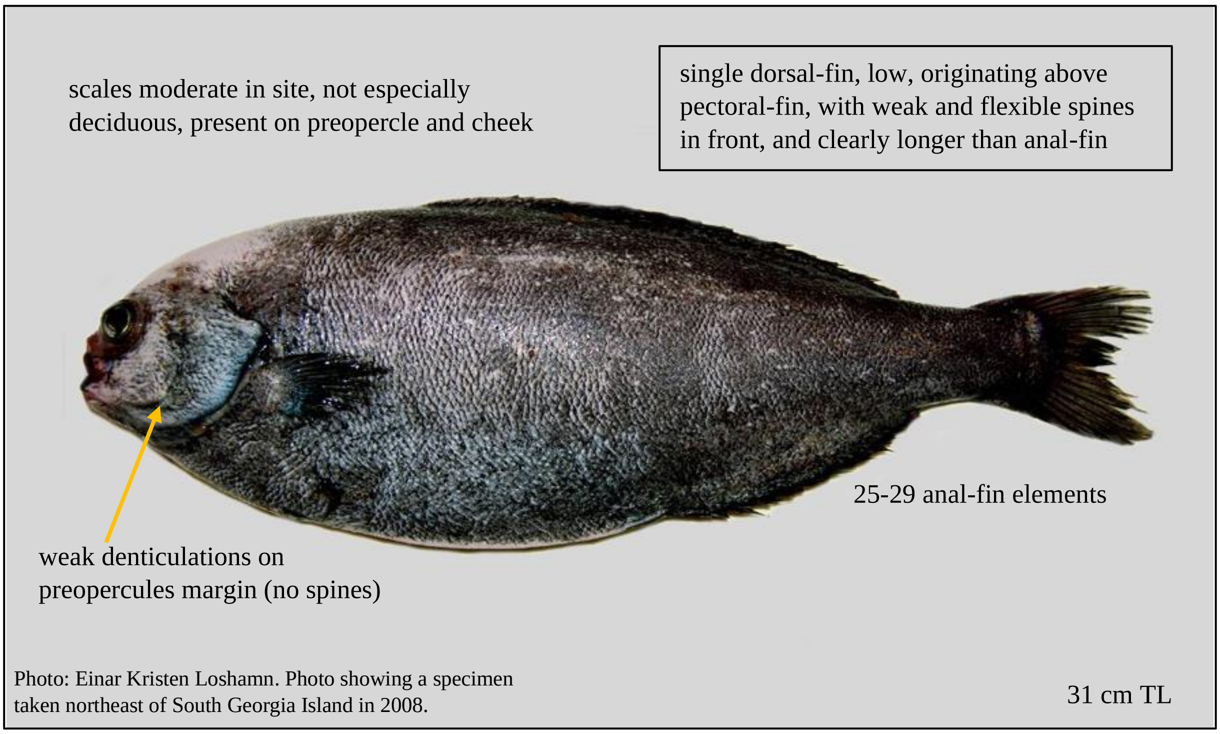

Photo: Einar Kristen Loshamn. Photo showing a specimen taken northeast of South Georgia Island in 2008.

One damaged specimen from the trawl, was caught at station 4019, 40 cm TL.

Achiropsettidae - southern or armless flounders

Mancopsetta maculata (Günther, 1880) - Antarctic armless flounder

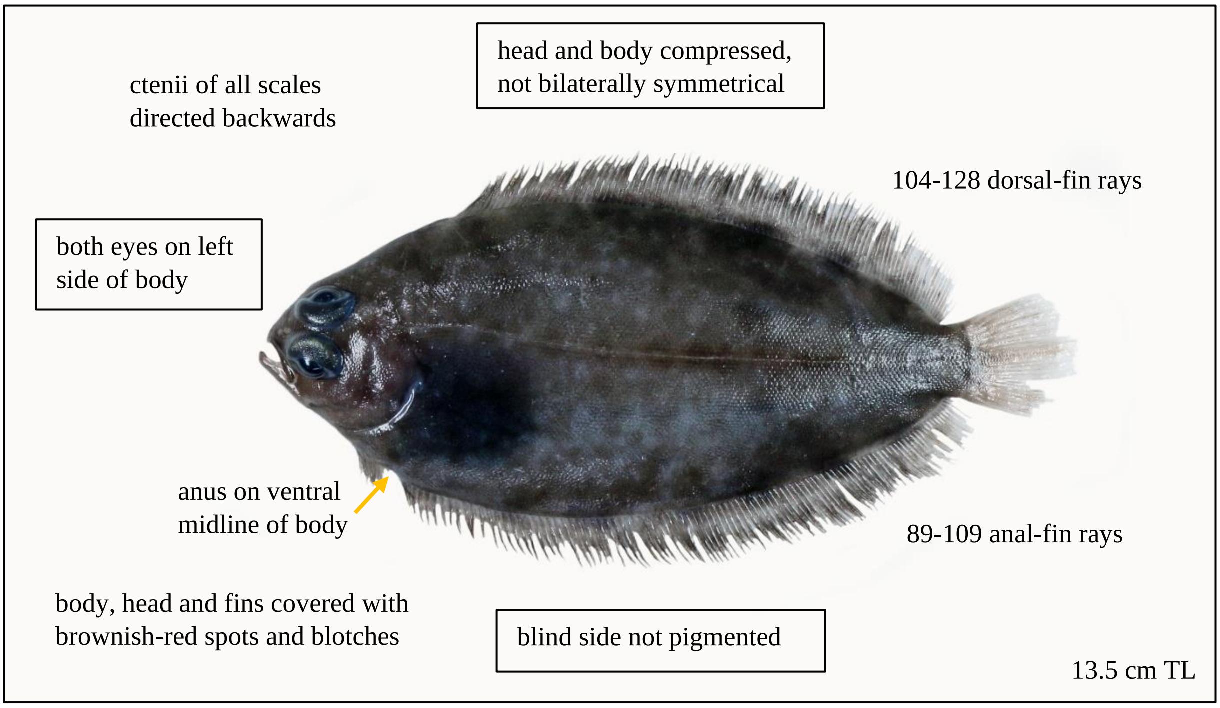

The specimen was caught at station 4019.

Nototheniidae - cod icefishes

Aethotaxis mitopteryx DeWitt, 1962 - longfin icedevil

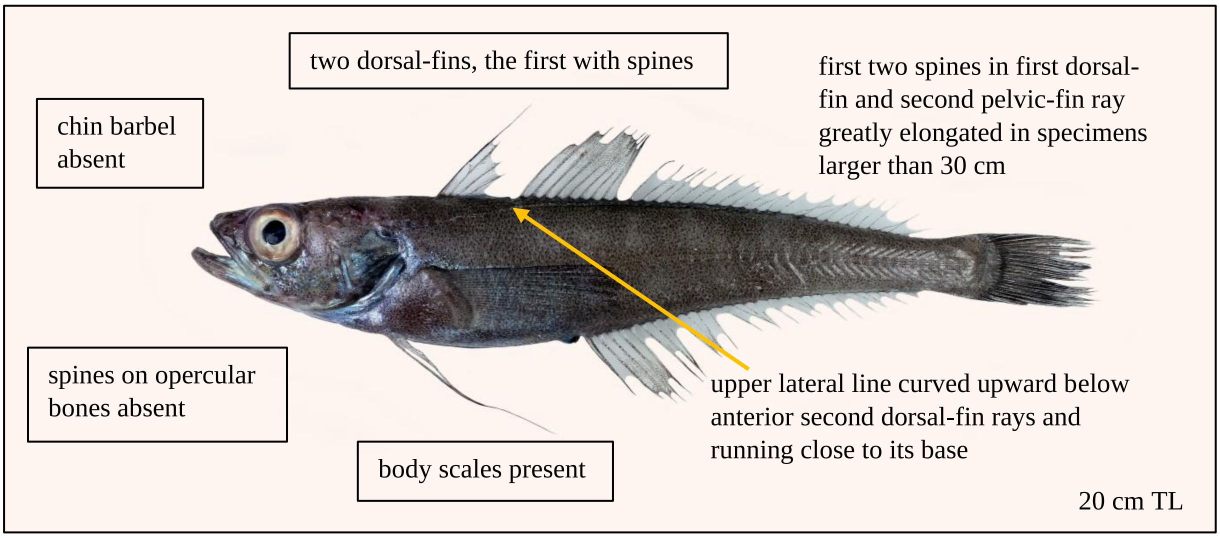

Three specimens were caught at station 4009, ranging in length from 20 to 24.5 cm TL.

Nototheniidae - cod icefishes

Dissostichus mawsoni Norman, 1937 - Antarctic toothfish

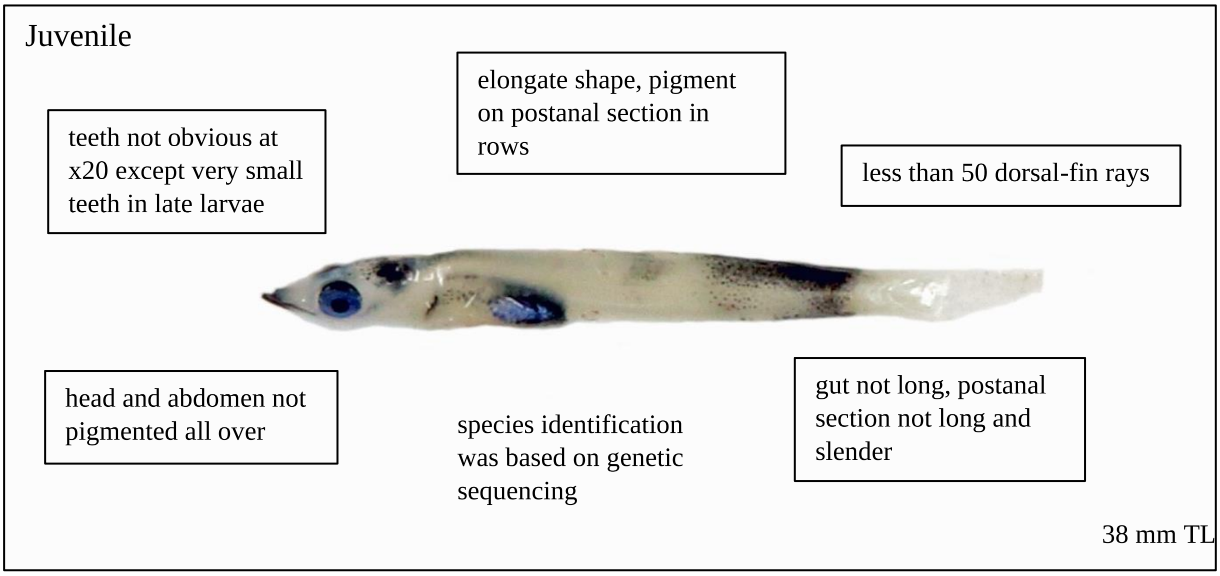

The specimen was caught at station 4062.

Nototheniidae - cod icefishes

Lepidonotothen squamifrons (Günther, 1880) - grey rockcod

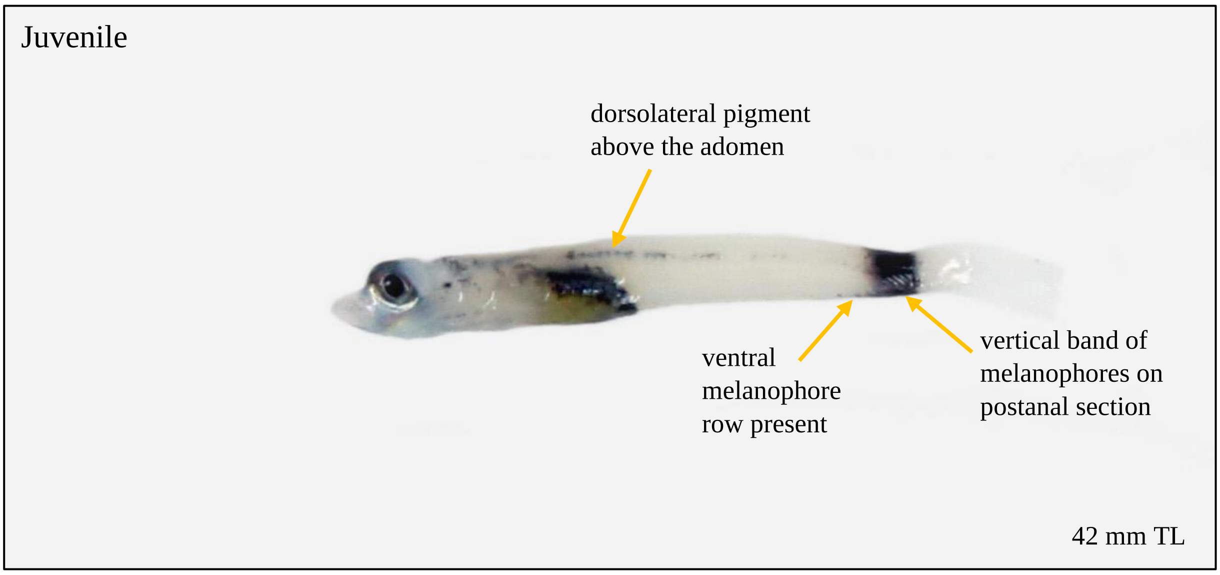

Four specimens were caught, ranging in length from 42 to 48 mm TL.

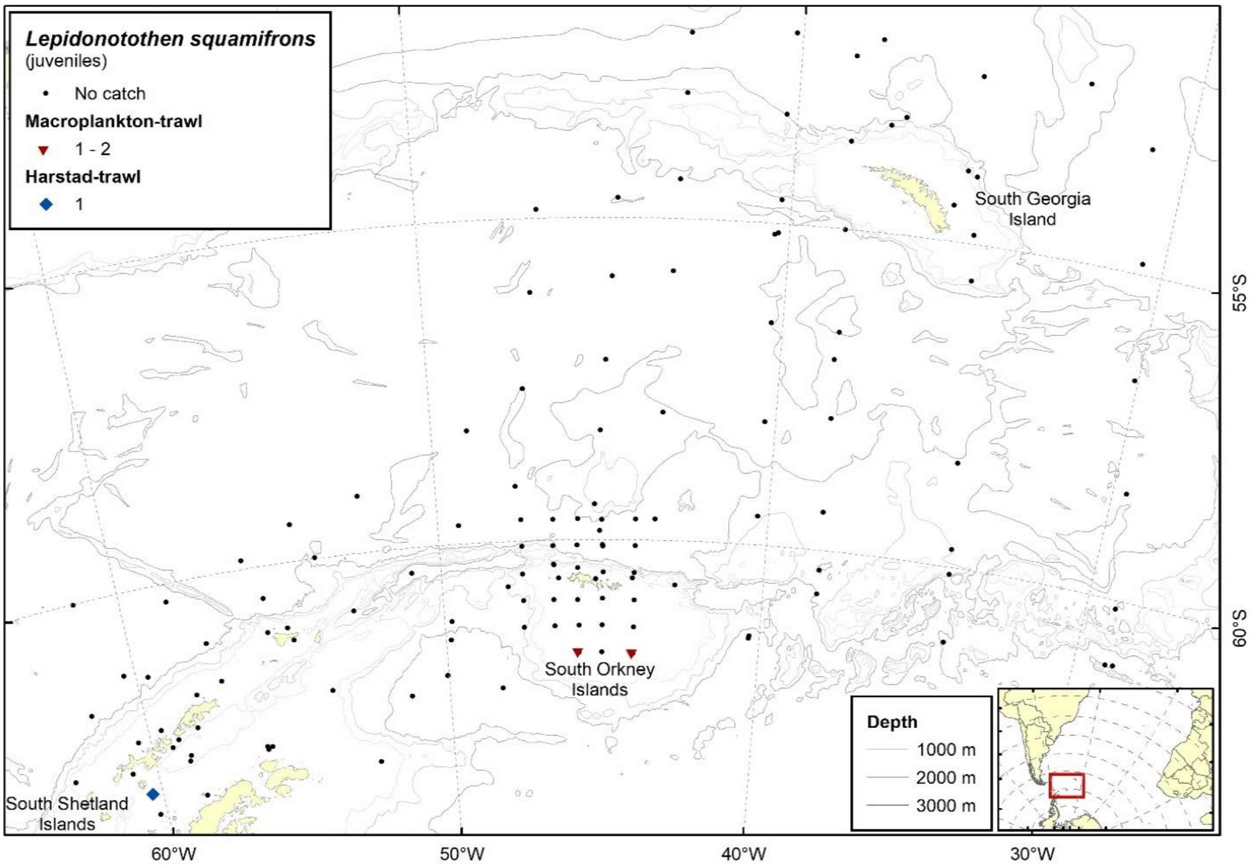

Nototheniidae - cod icefishes

Notothenia coriiceps Richardson, 1844 - black rockcod

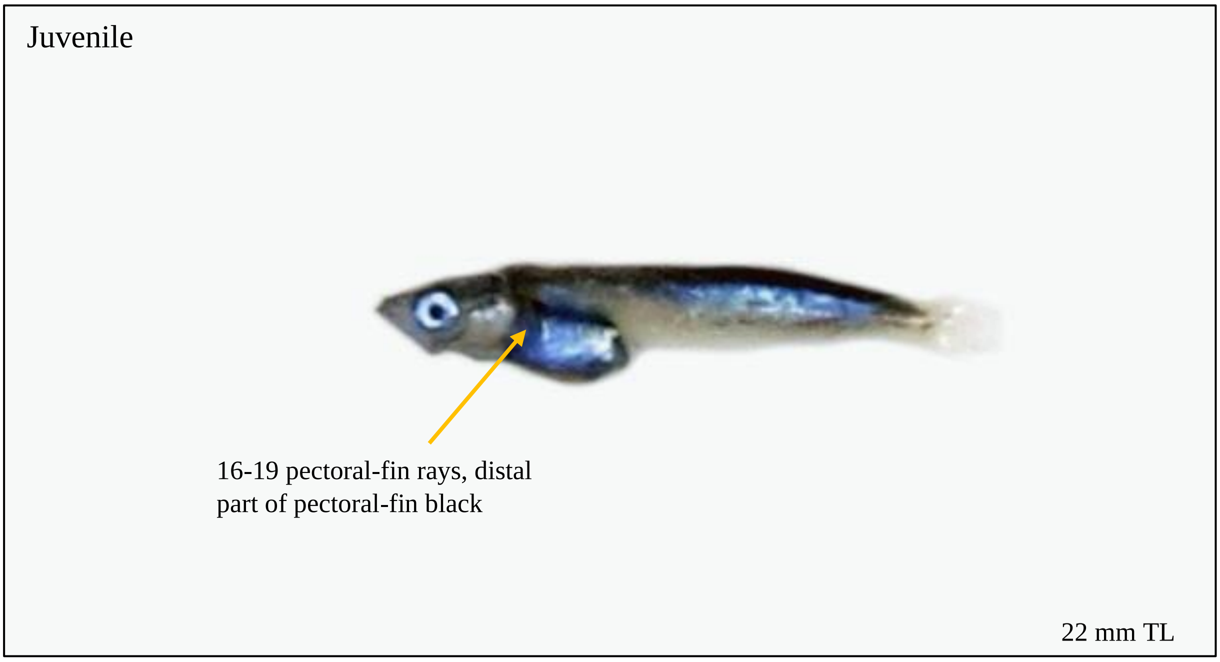

Nine specimens were caught, ranging in length from 22 to 34 mm TL.

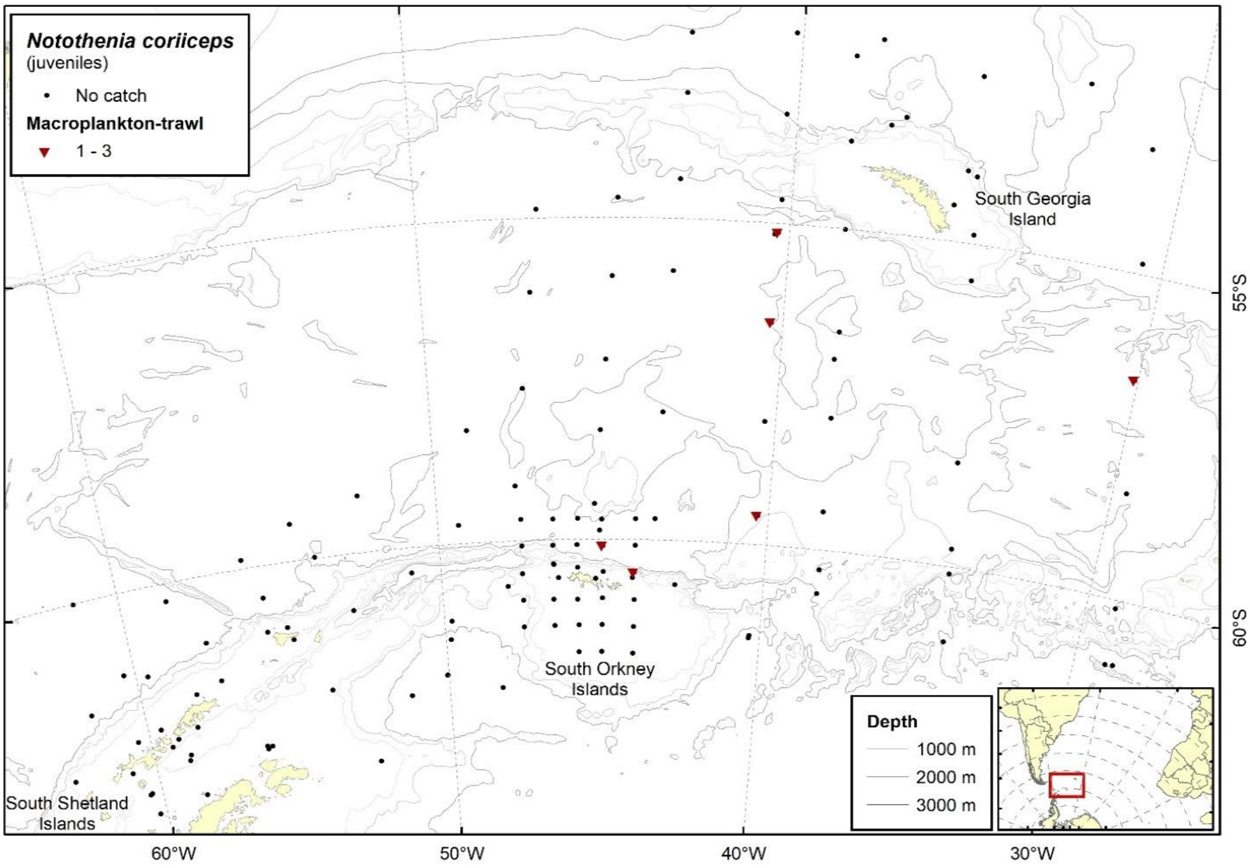

Nototheniidae - cod icefishes

Notothenia neglecta Nybelin, 1951 - yellowbelly rockcod

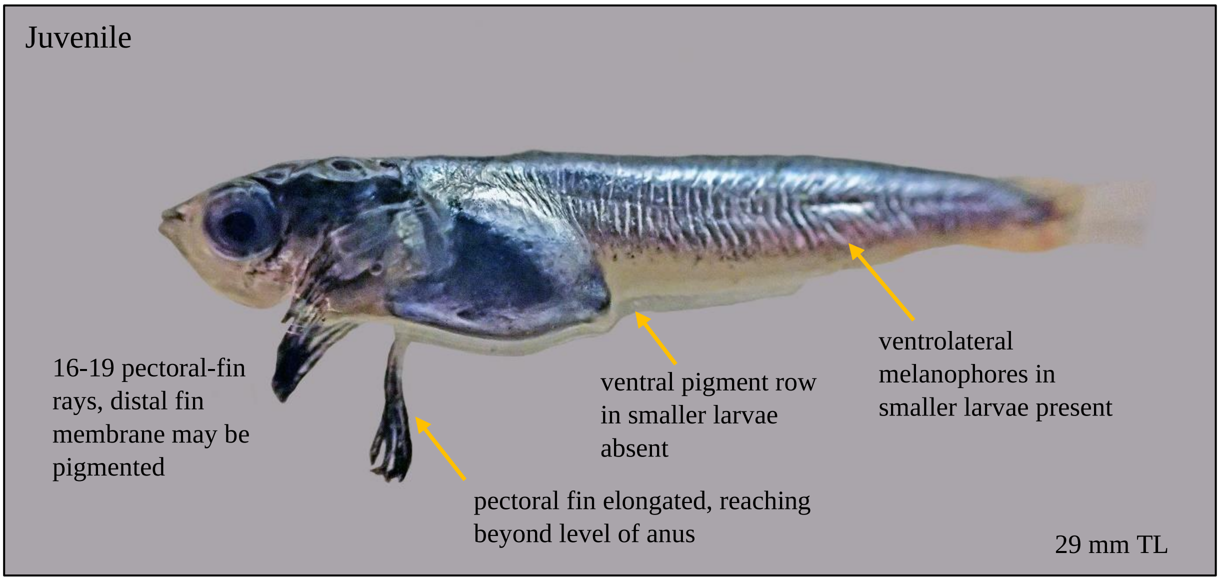

One specimen was caught at station 4271.

Nototheniidae - cod icefishes

Notothenia rossii Richardson, 1844 - marbled rockcod

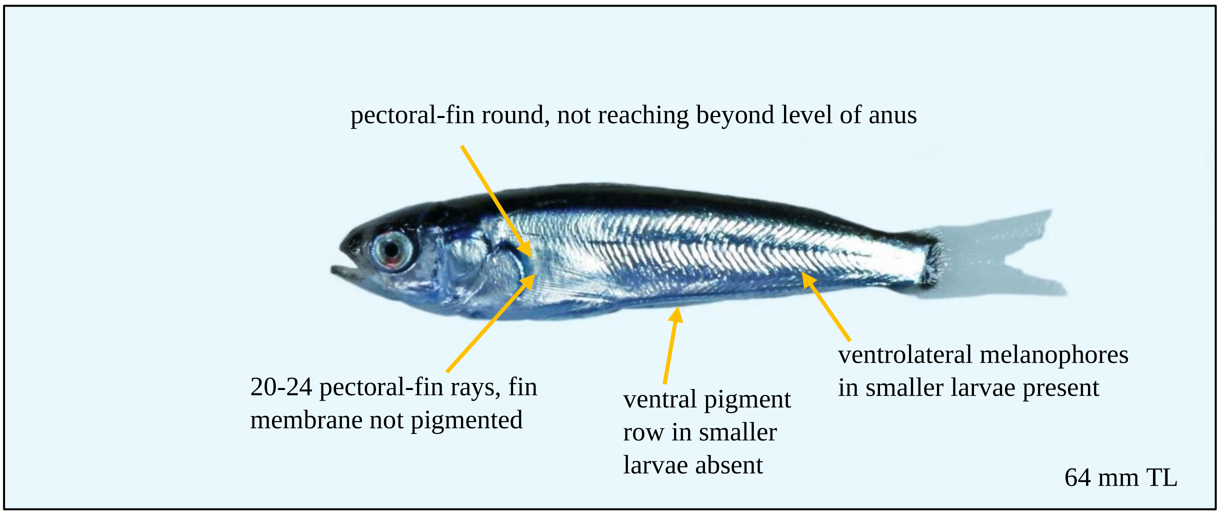

Nine specimens were caught, ranging in length from 34 to 65 mm TL.

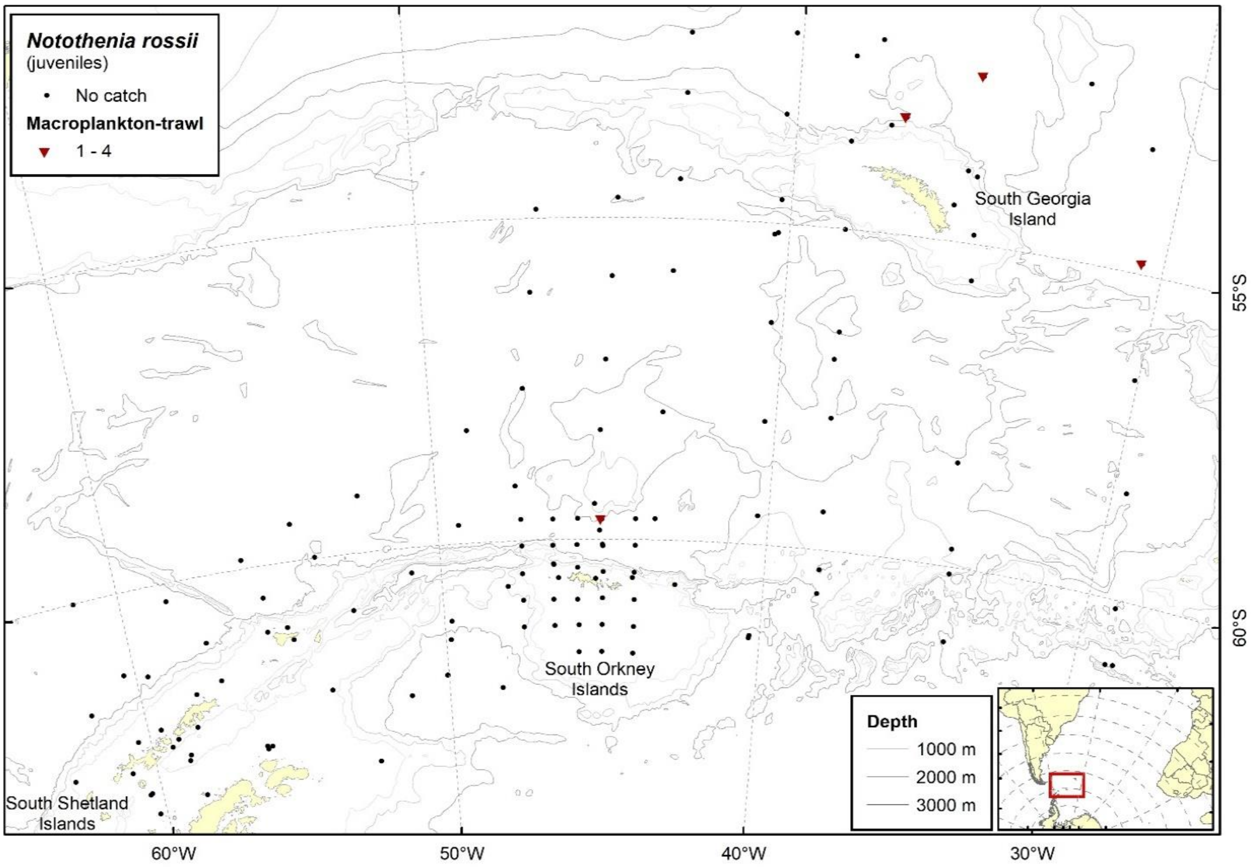

Nototheniidae - cod icefishes

Nototheniops larseni (Lönnberg, 1905) - painted notie

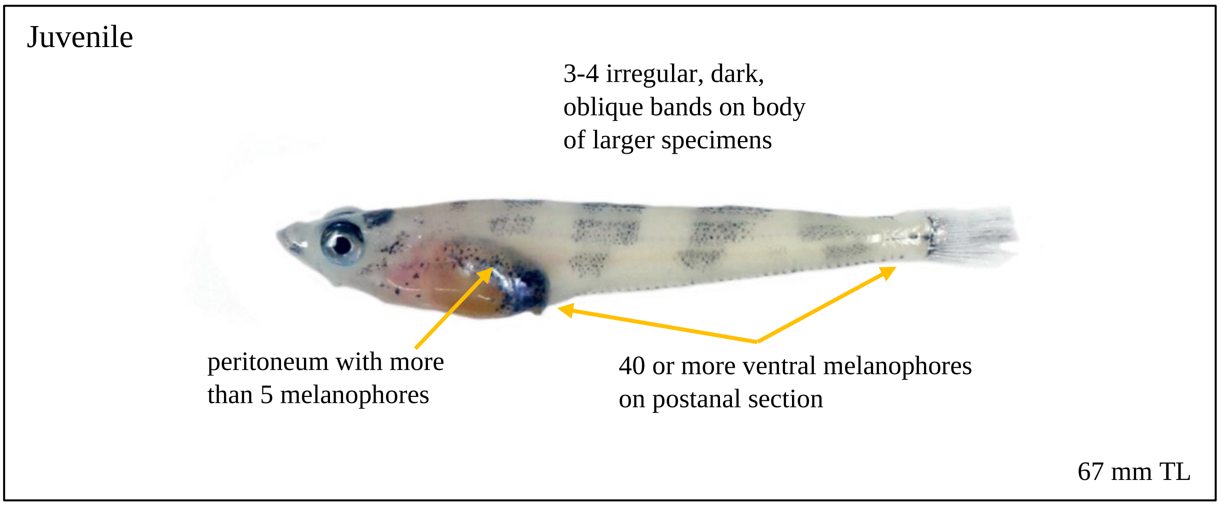

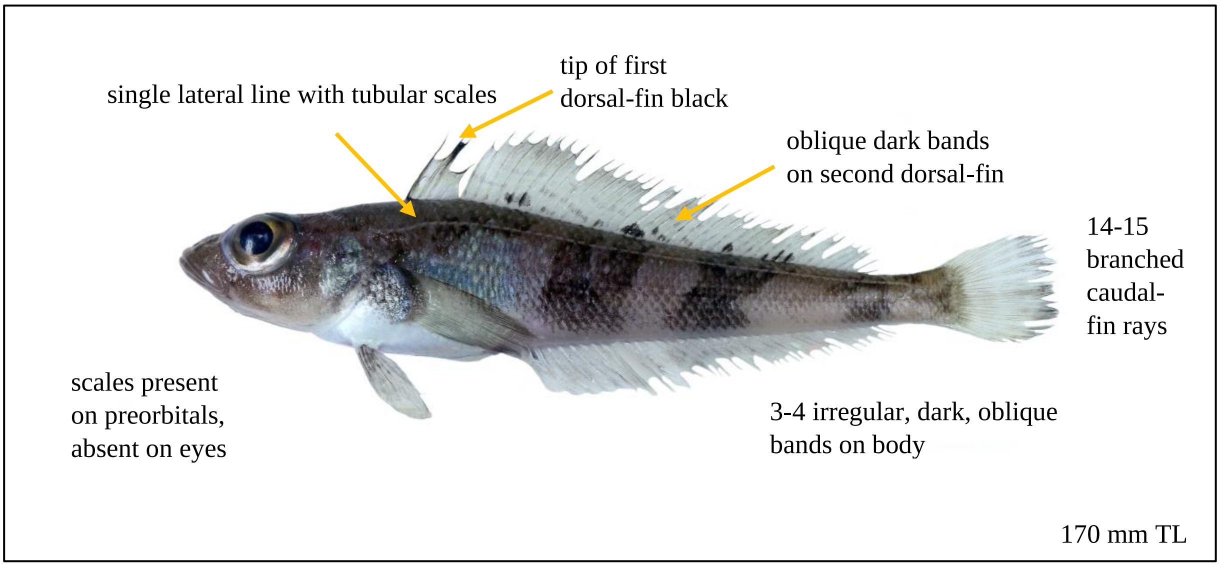

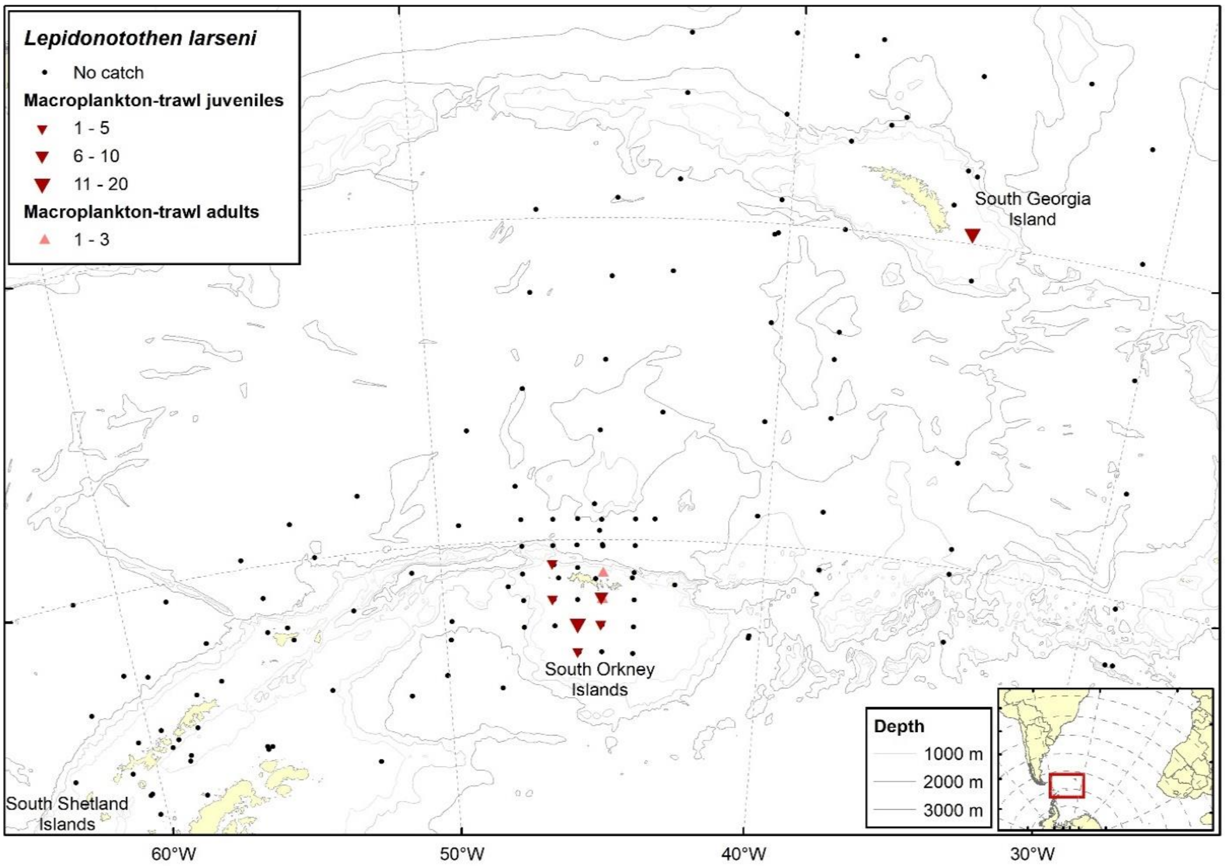

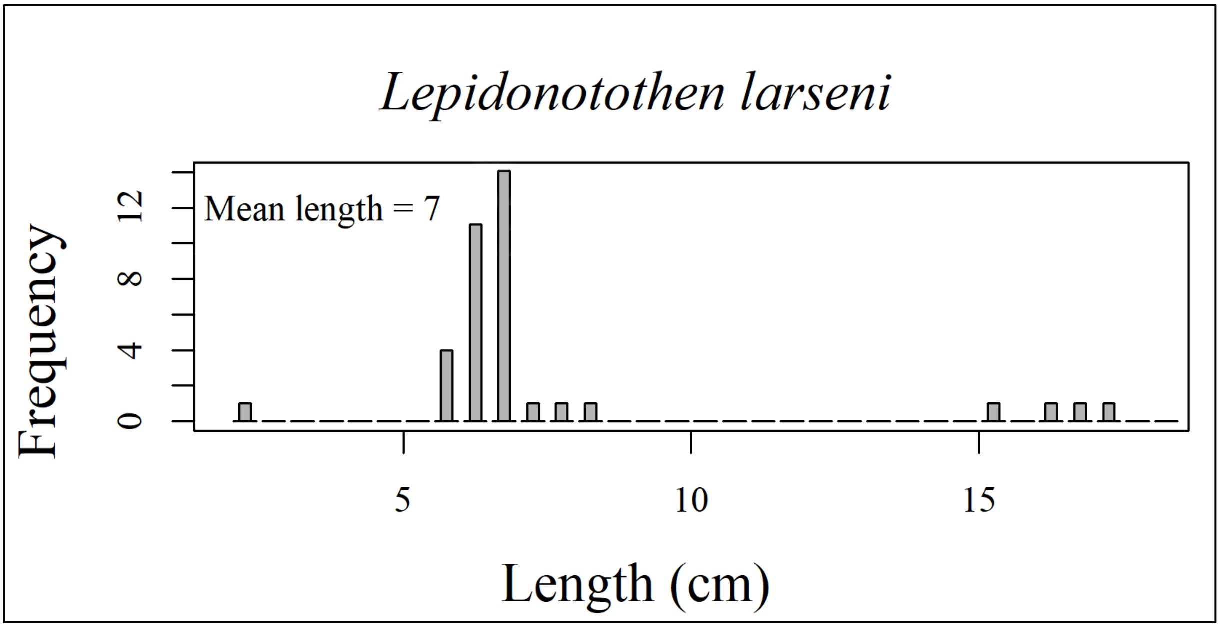

Length distribution of *Nototheniops larseni*, N = 37.

Nototheniidae - cod icefishes

Pleuragramma antarcticum Boulenger, 1902 - Antarctic silverfish

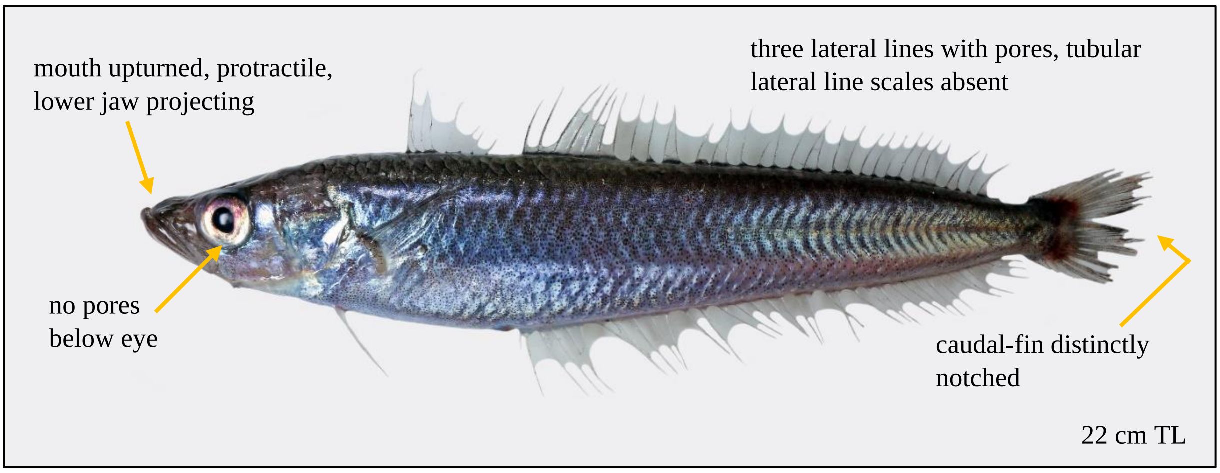

Seventeen specimens were caught, ranging in length from 10.5 to 23.5 cm TL.

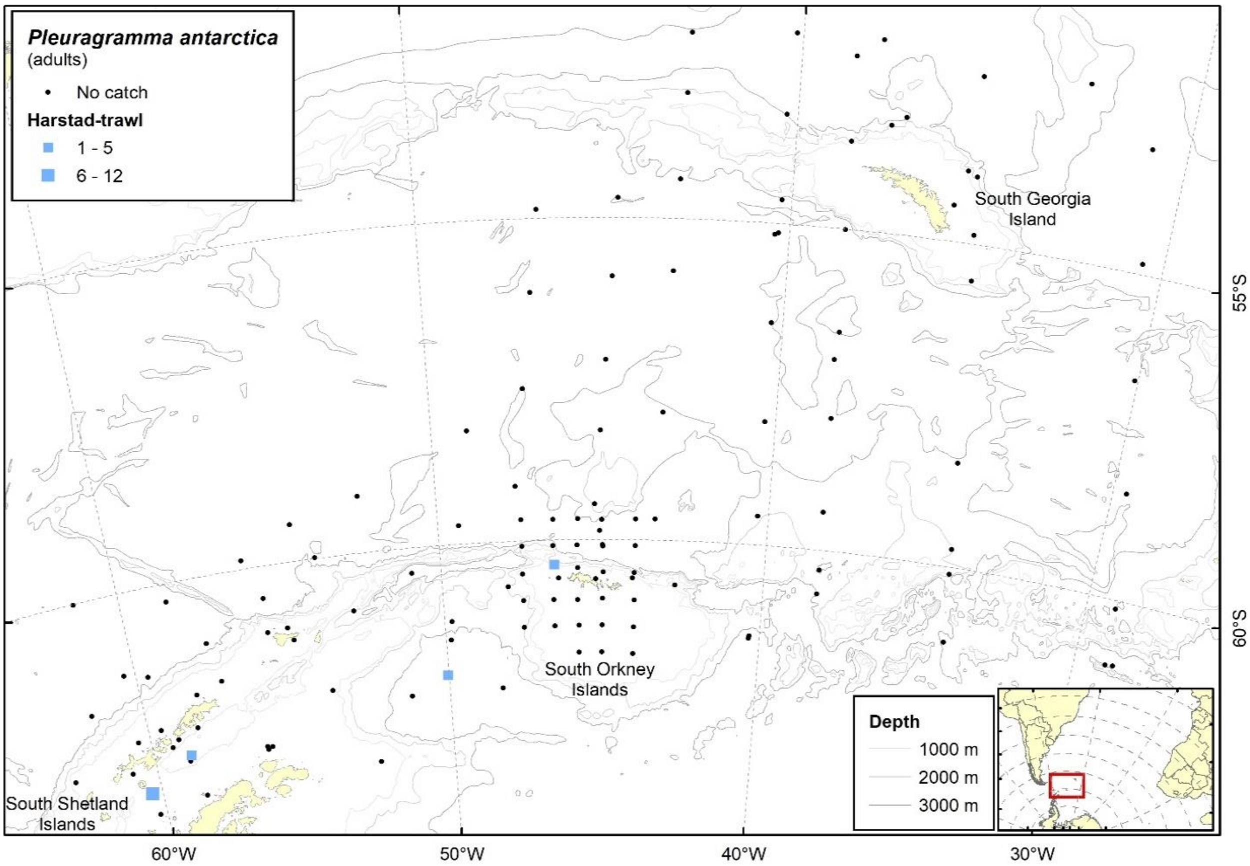

Nototheniidae - cod icefishes

Pseudotrematomus eulepidotus Regan, 1914 - blunt scalyhead

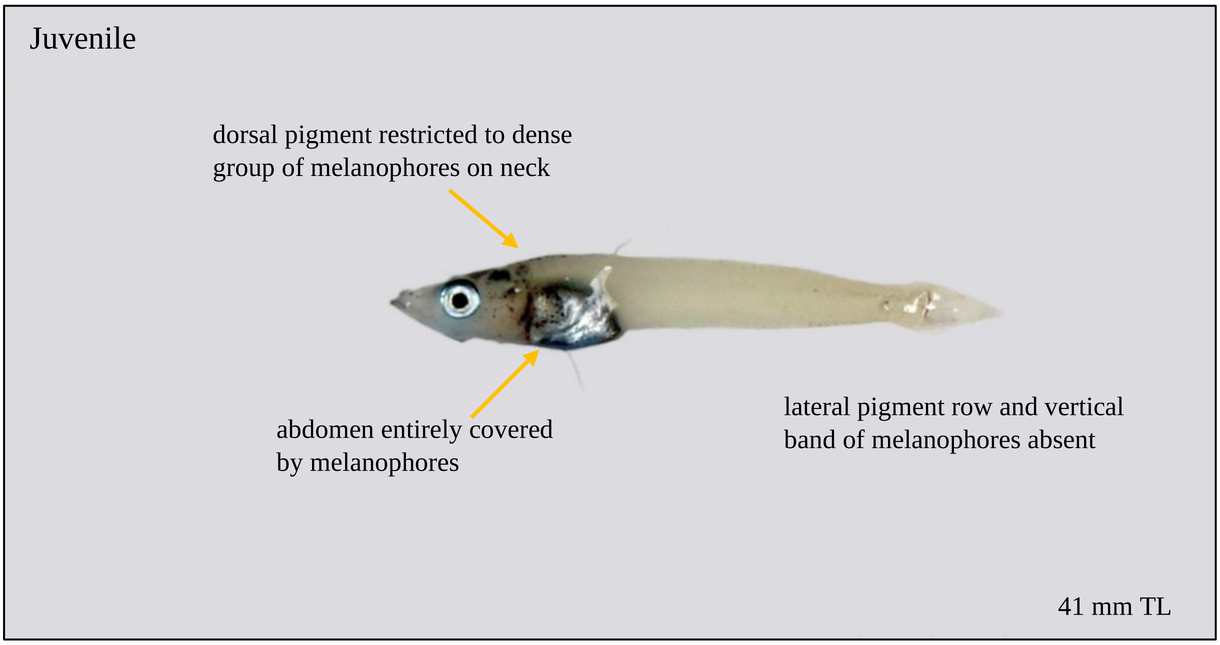

Three specimens (35-42 mm TL) were caught at station 4009 and 4029. The species identification was genetically verified.

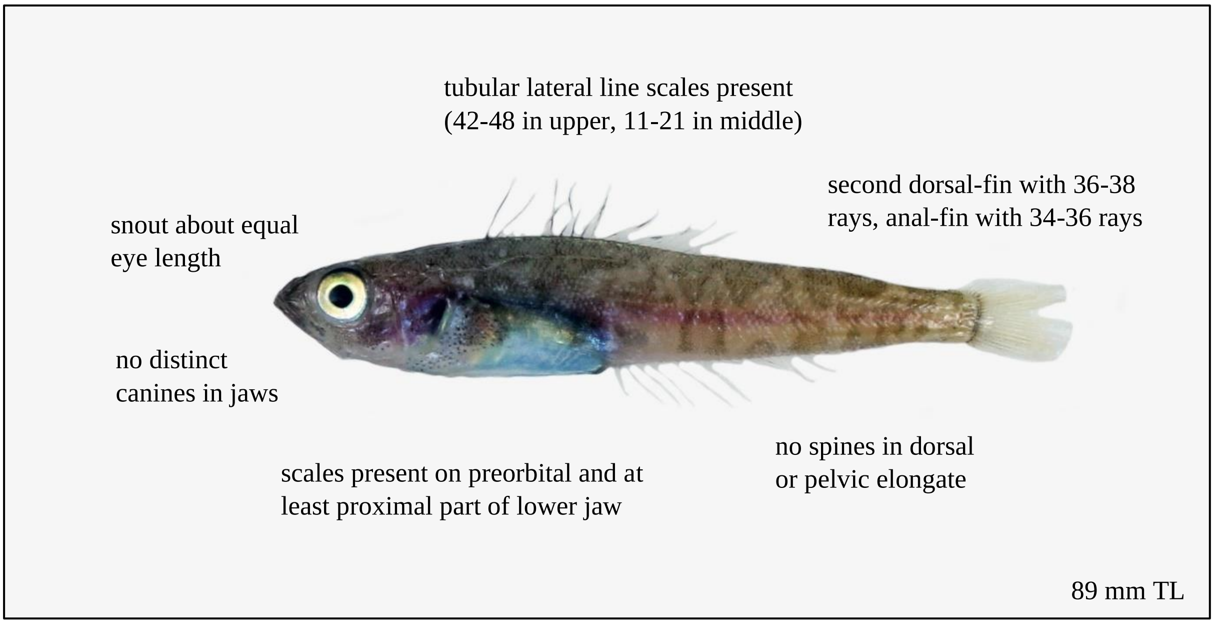

Three specimens were caught at station 4009 and 4029, ranging in length from 41 to 90 mm TL.

Nototheniidae - cod icefishes

Trematomus newnesi Boulenger, 1902 - dusky rockcod

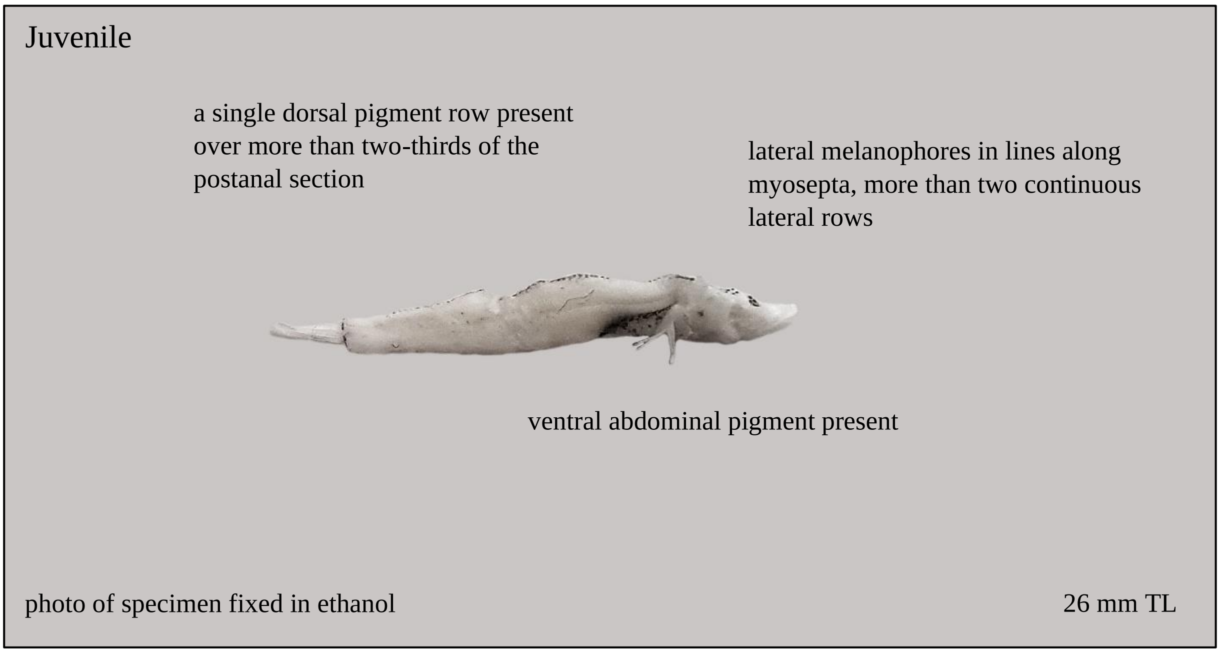

One specimen was caught at station 4030.

Nototheniidae - cod icefishes

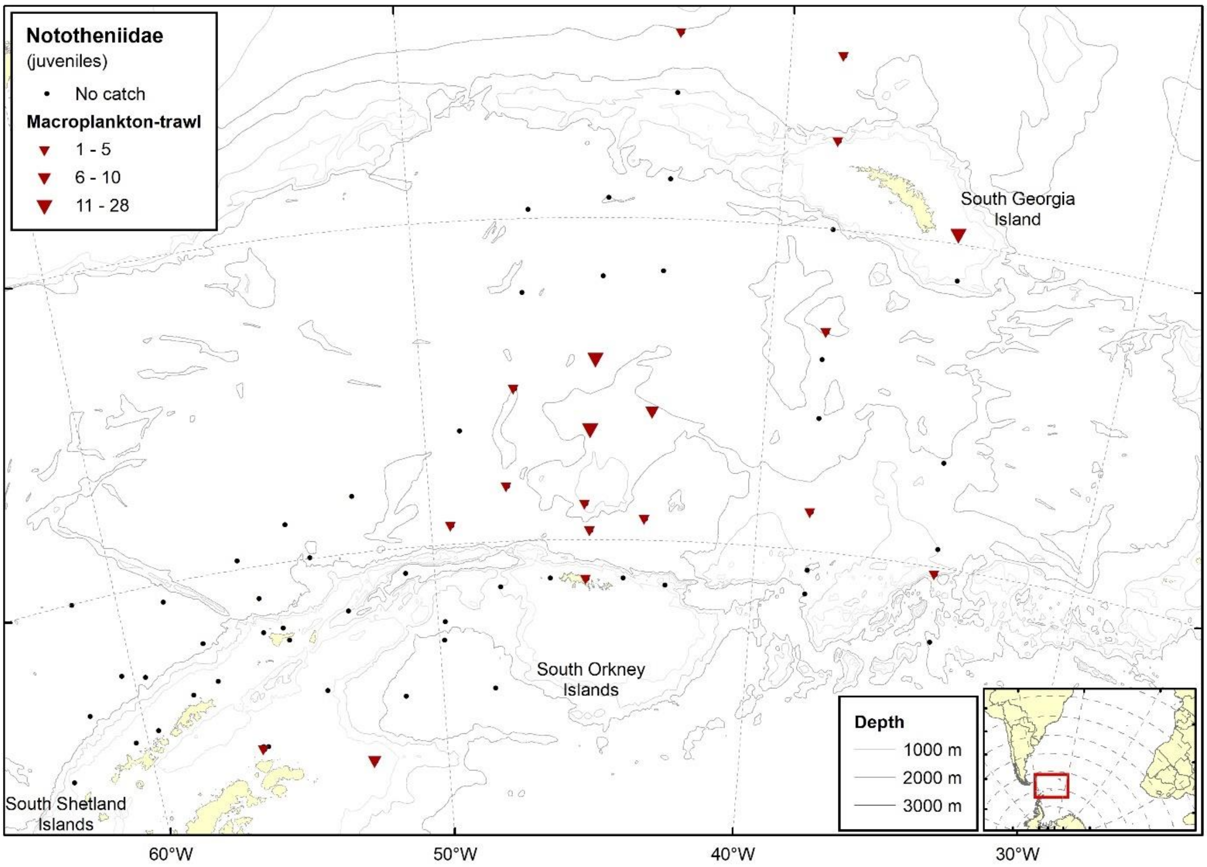

101 specimens taken on FV *Cabo de Hornos* could - based on pictures - not be verified to genus or species level.

Harpagiferidae - plunderfishes

One specimen (26 mm TL) was caught at station 4039, not identified to species or genus level.

Harpagiferidae - plunderfishes

Harpagifer sp. - juvenile

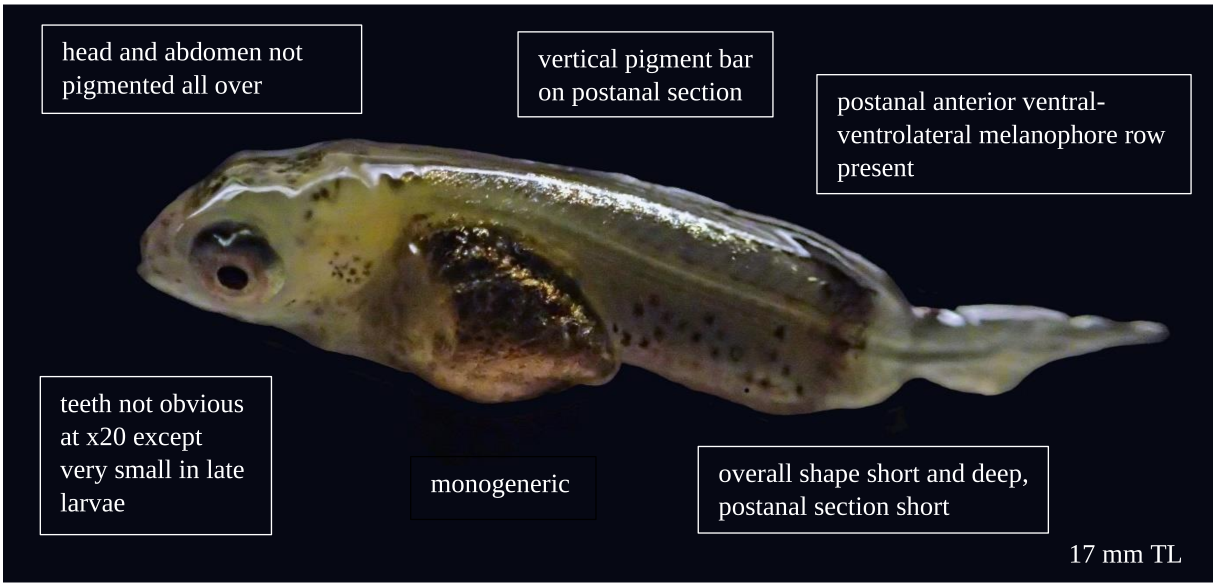

One specimen was caught at station 4265.

Harpagiferidae - plunderfishes

Neodraco skottsbergi (Lönnberg, 1905)

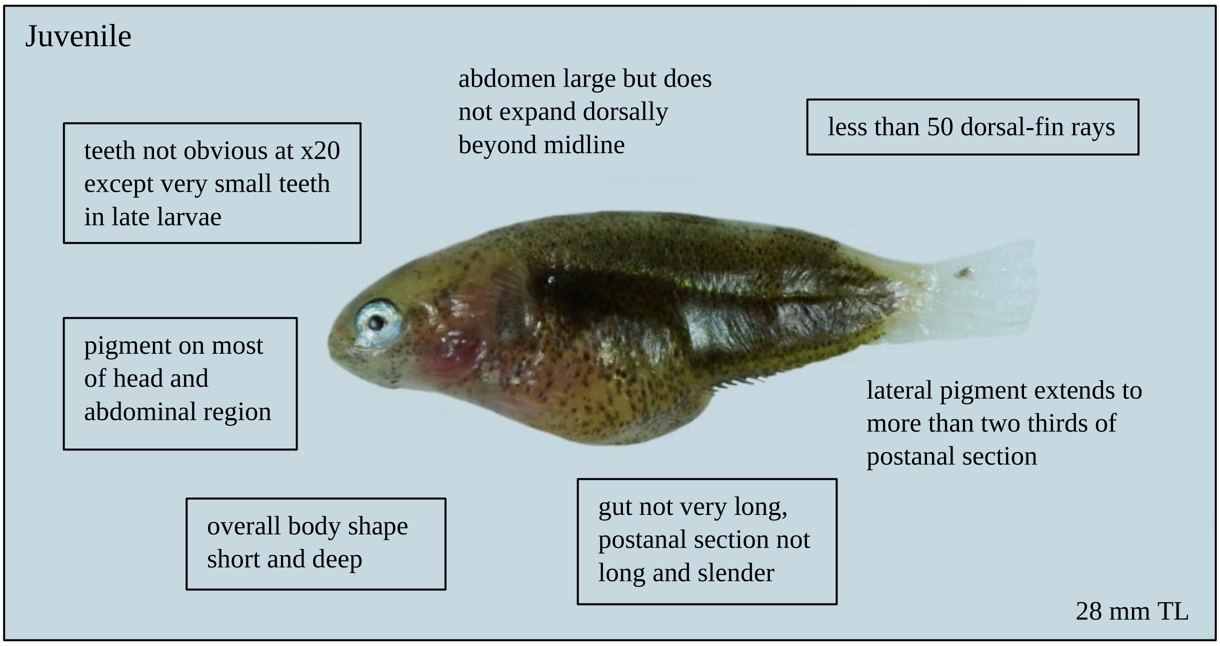

Thirteen specimens were caught at station 4026 and 4038, ranging in length from 20 to 30 mm TL.

Harpagiferidae - plunderfishes

Pogonophryne scotti Regan, 1914 - saddleback plunderfish

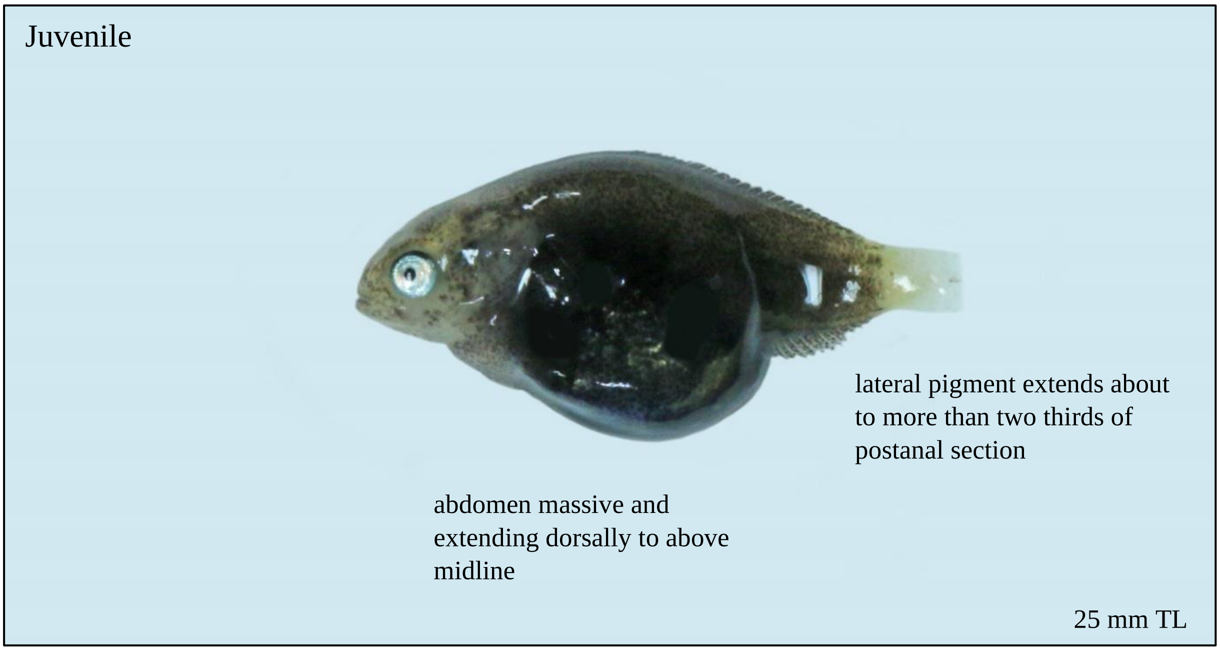

One specimen was caught at station 4038.

Bathydraconidae - Antarctic dragonfishes

Bathydraco spp. – juvenile

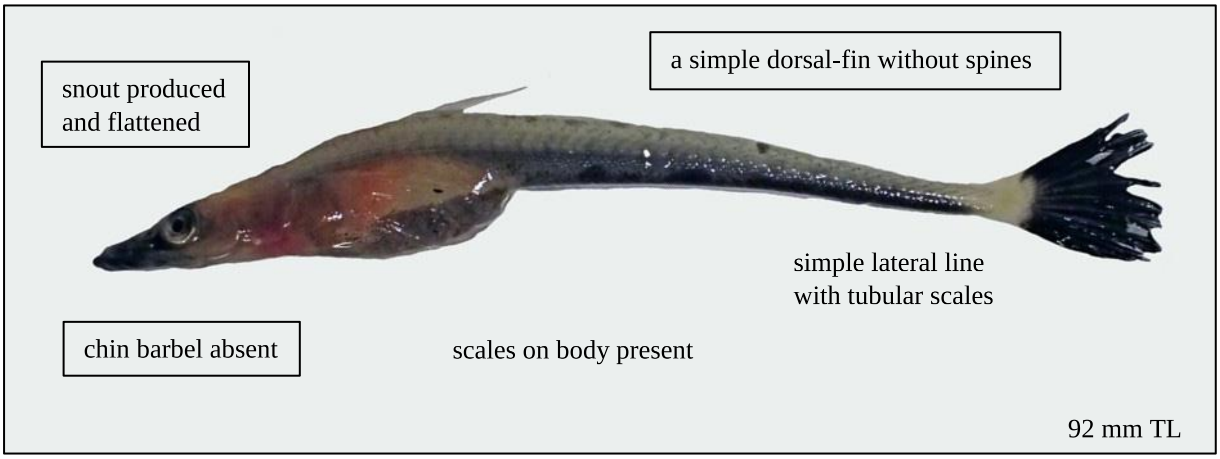

Two specimens were caught at station 4299 and 4307, 82 and 92 mm TL.

Bathydraconidae - Antarctic dragonfishes

Gymnodraco acuticeps Boulenger, 1902 - ploughfish

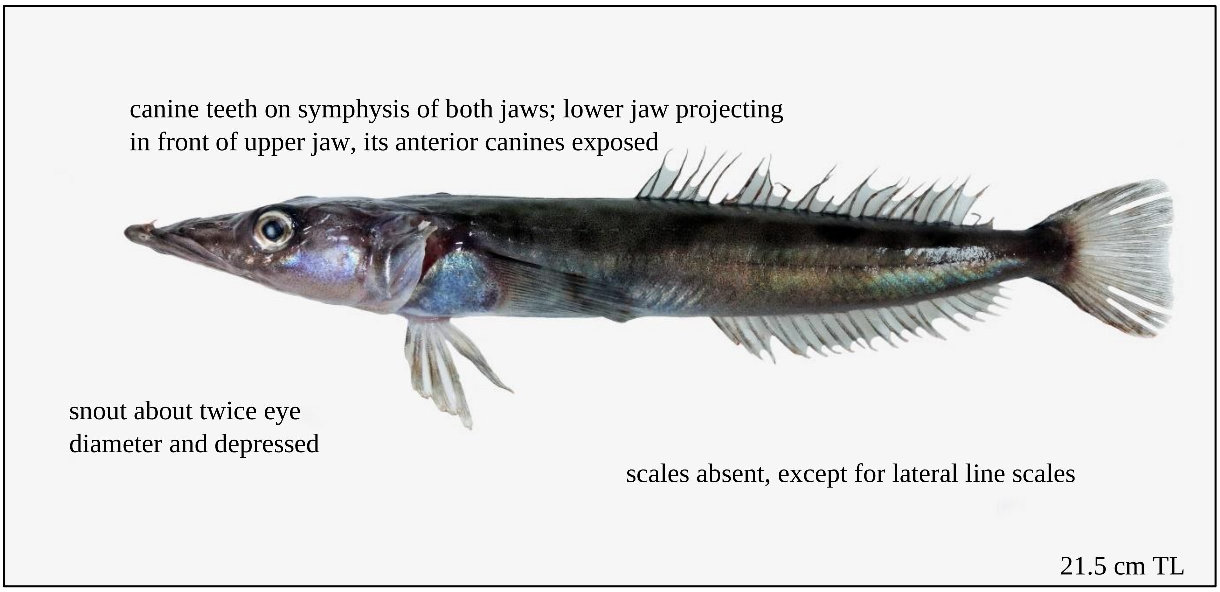

One specimen was caught at station 4067.

Bathydraconidae - Antarctic dragonfishes

Prionodraco evansii Regan, 1914

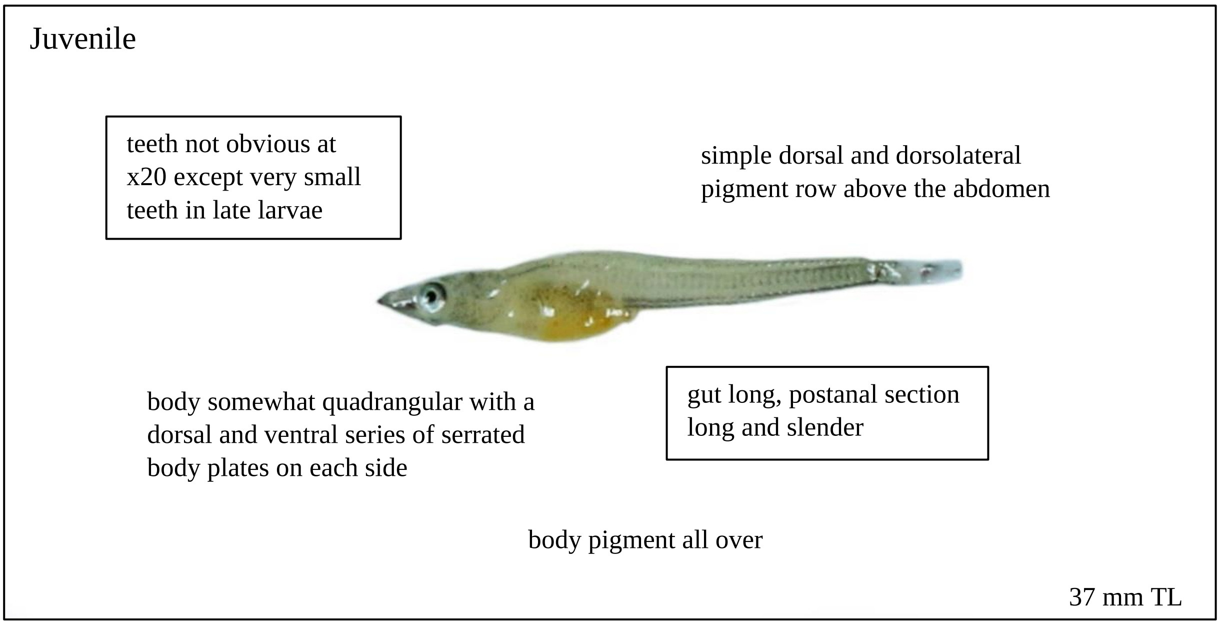

Seven specimens were caught, ranging in length from 26 to 40 mm TL.

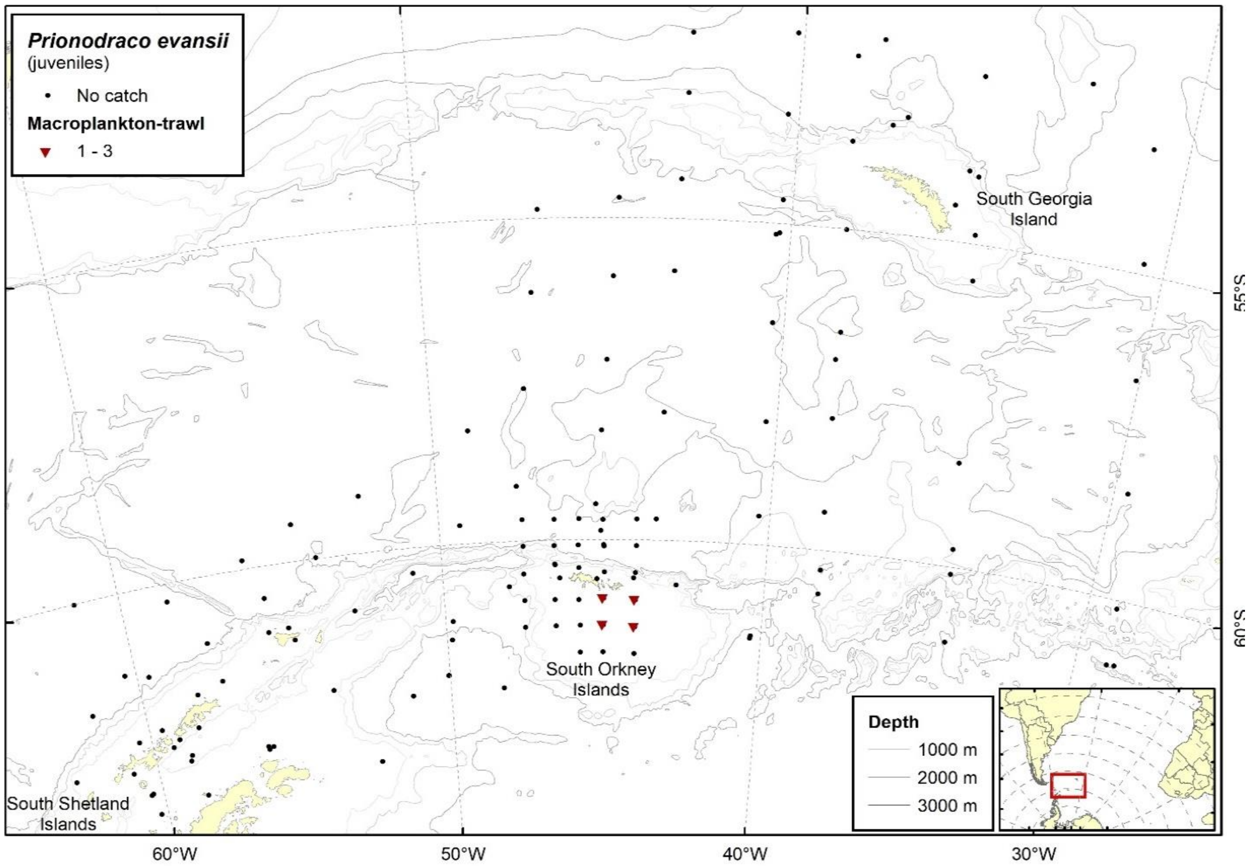
Prionodraco evansii
(juveniles)
No catch
Macroplankton-trawl
1 - 3
South Georgia Island
South Orkney Islands
South Shetland Islands
Depth
1000 m
2000 m
3000 m
55°S
60°S
60°W
50°W
40°W
30°W

Channichthyidae - crocodile icefishes

Chaenocephalus aceratus (Lönnberg, 1906) - blackfin icefish

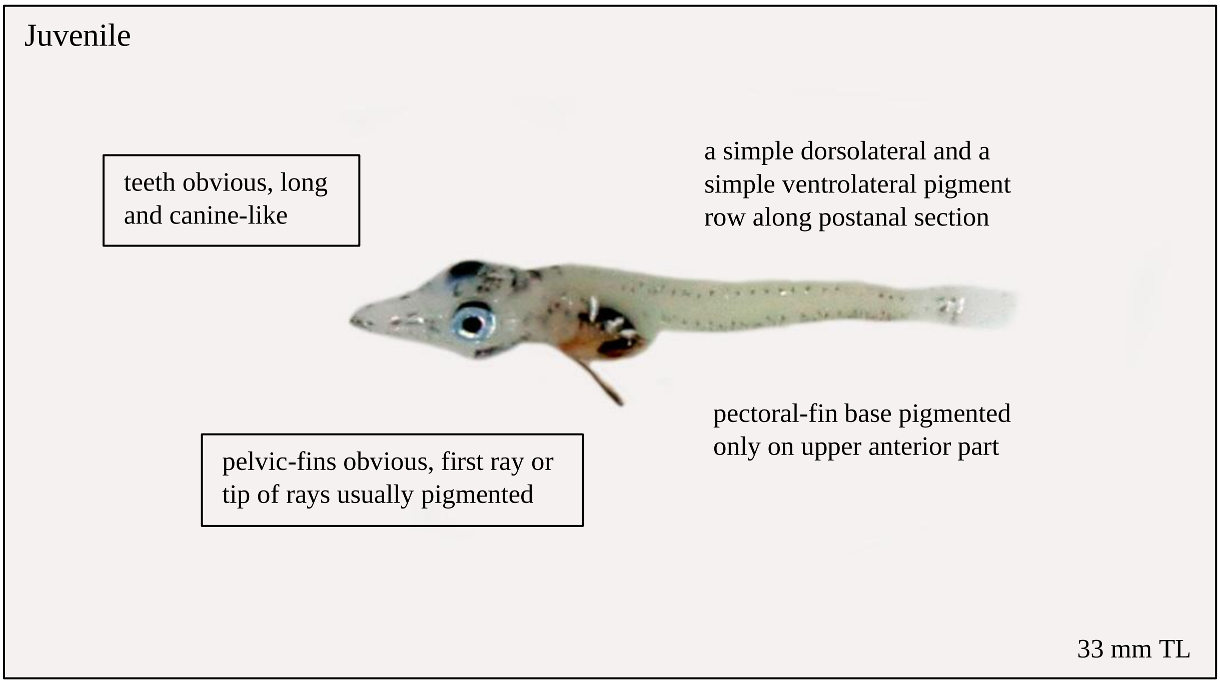

There were caught 23 juvenile specimens ranging in length from 29-53 mm TL (mean 40 mm), and one adult specimen with 22 cm TL.

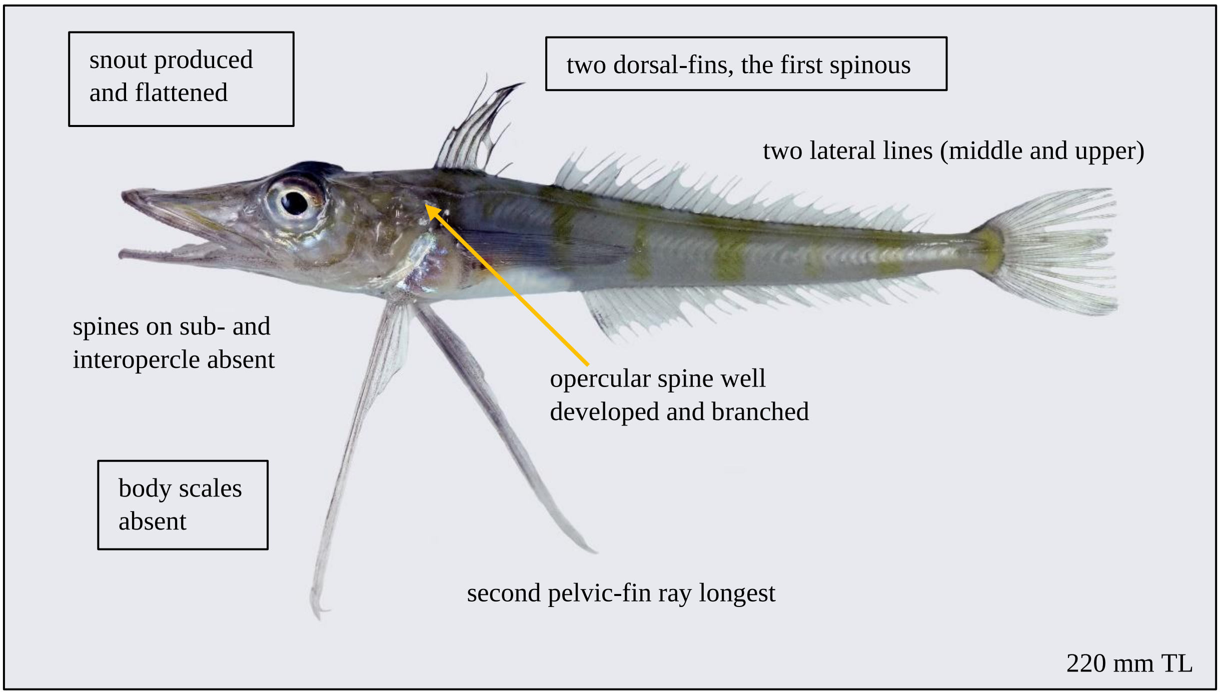

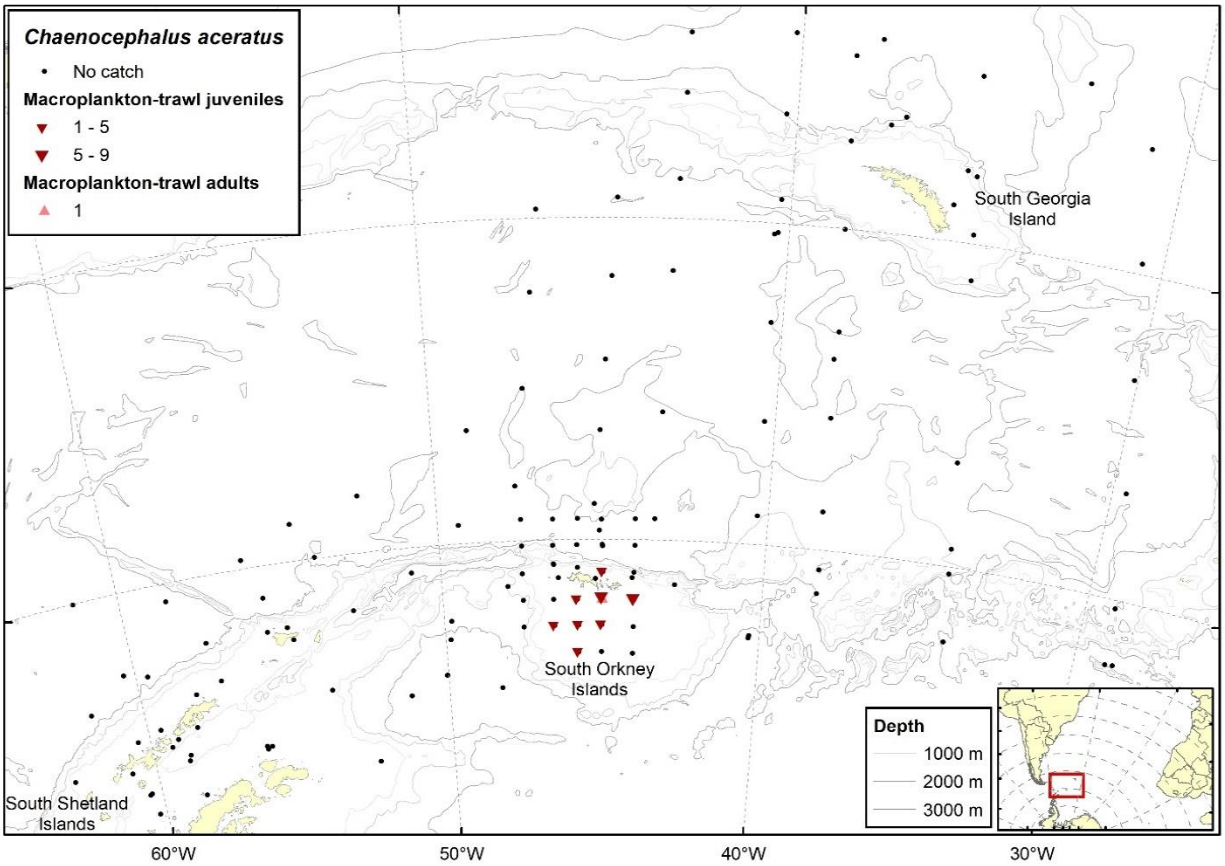

Channichthyidae - crocodile icefishes

Chaenodraco wilsoni Regan, 1914 - spiny icefish

Juvenile

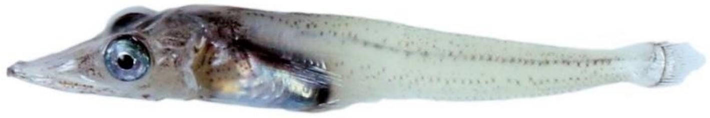

Seven specimens were caught, ranging in length from 46 to 70 mm TL.

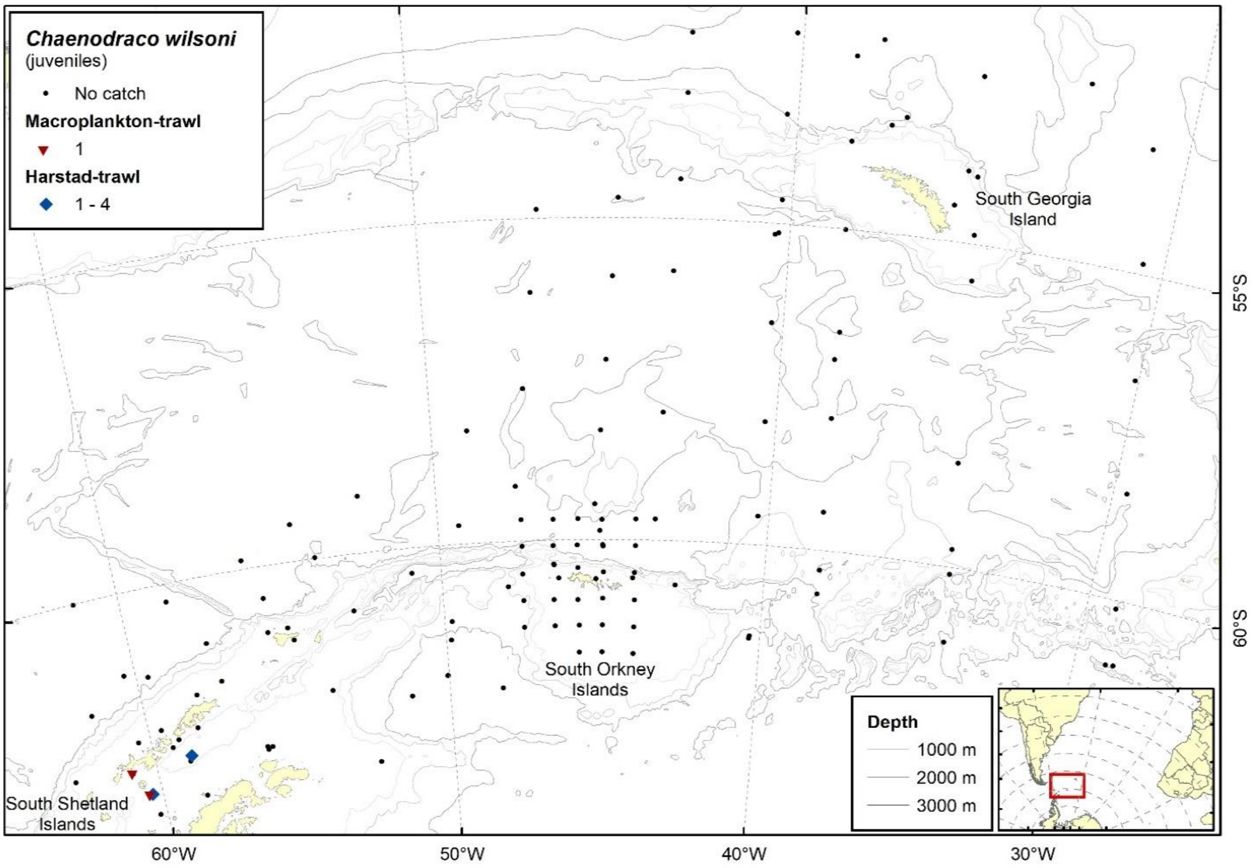

Channichthyidae - crocodile icefishes

Chionodraco rastrospinosus DeWitt & Hureau, 1979 - ocellated icefish

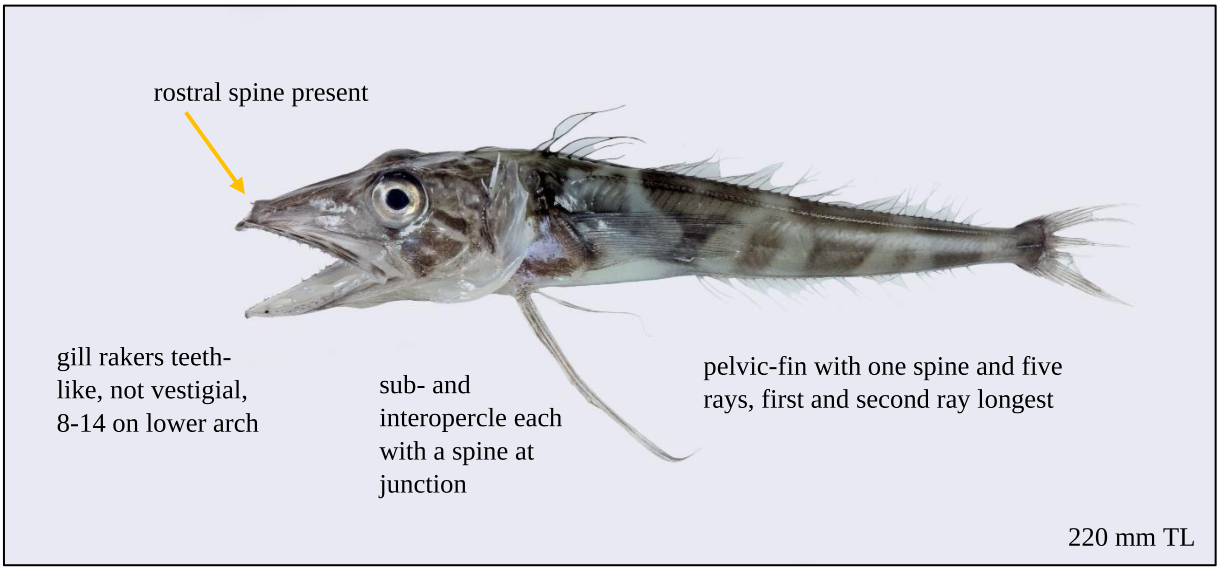

Juvenile

2-3 dorsolateral and 2-3 ventrolateral pigment rows on anterior postanal section and above abdomen

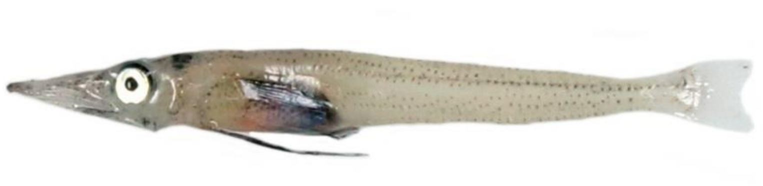

pelvic-fin with one spine and five rays

59 mm TL

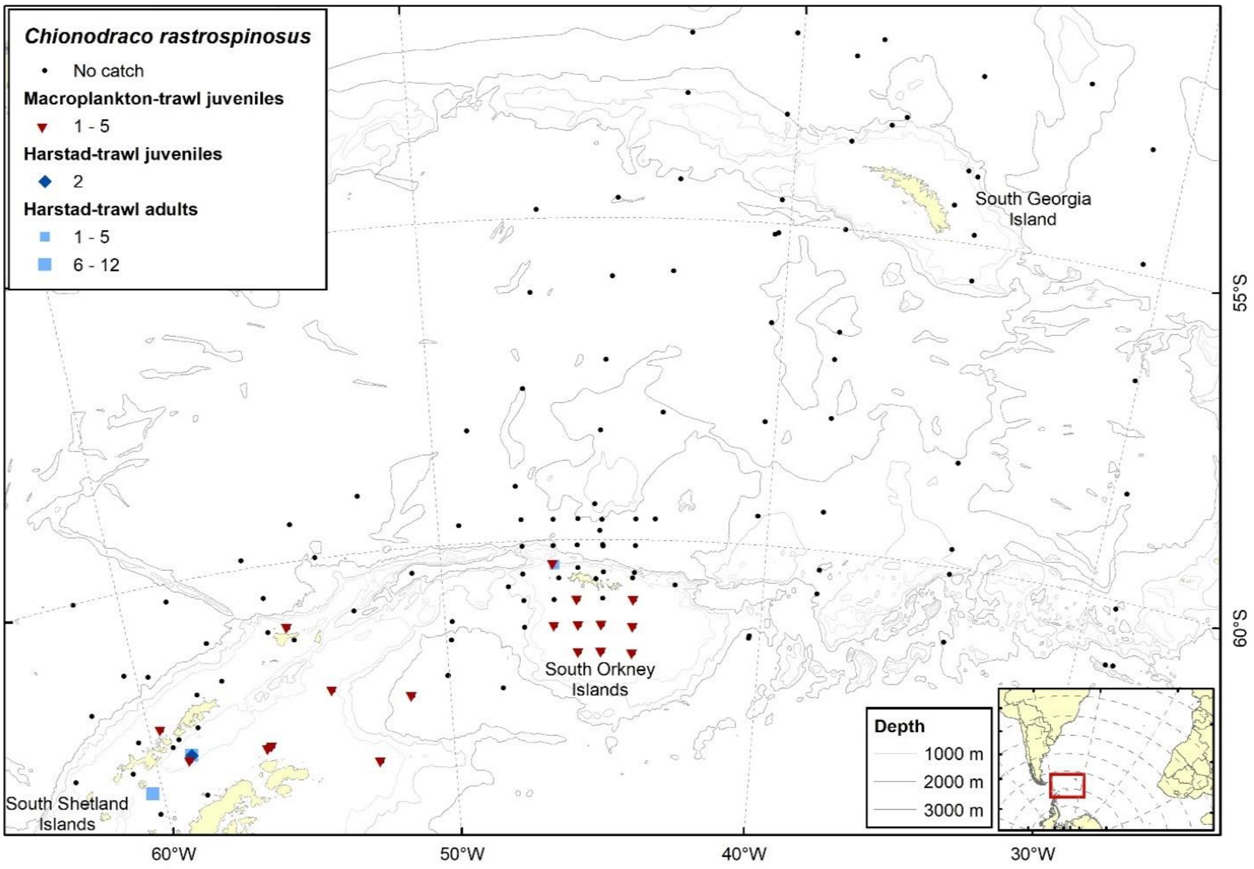

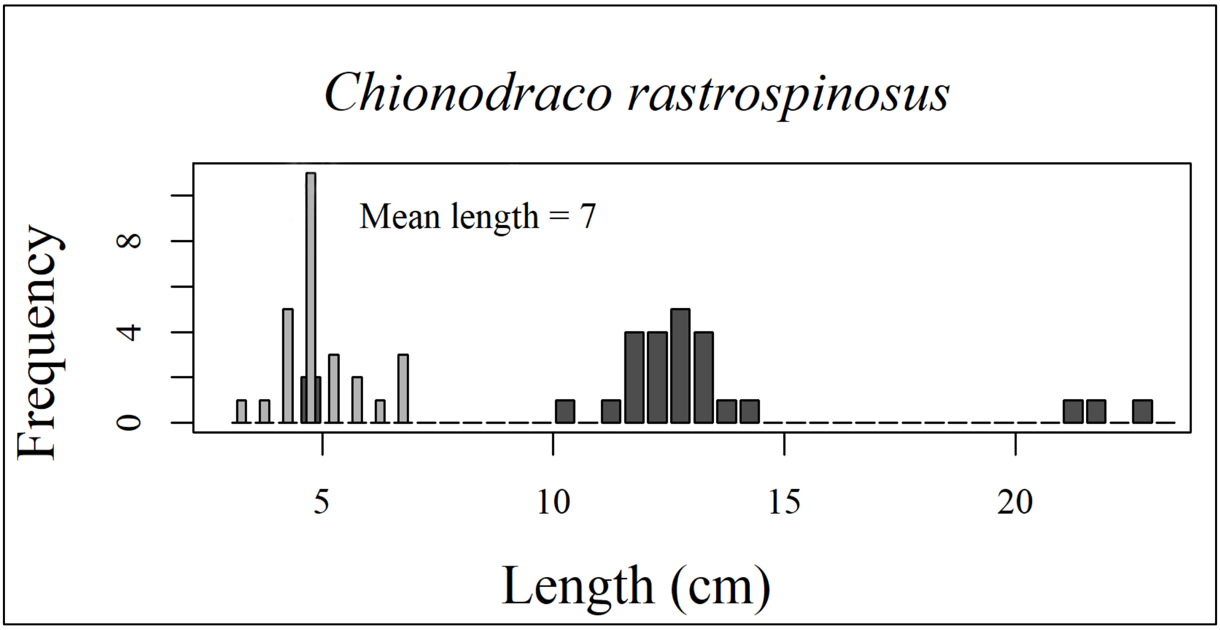

Length distribution of *C. rastrospinosus*, light grey: Macroplanktontrawl (N = 27), dark grey: Harstadtrawl (N = 26).

Channichthyidae - crocodile icefishes

Dacodraco hunteri Waite, 1916

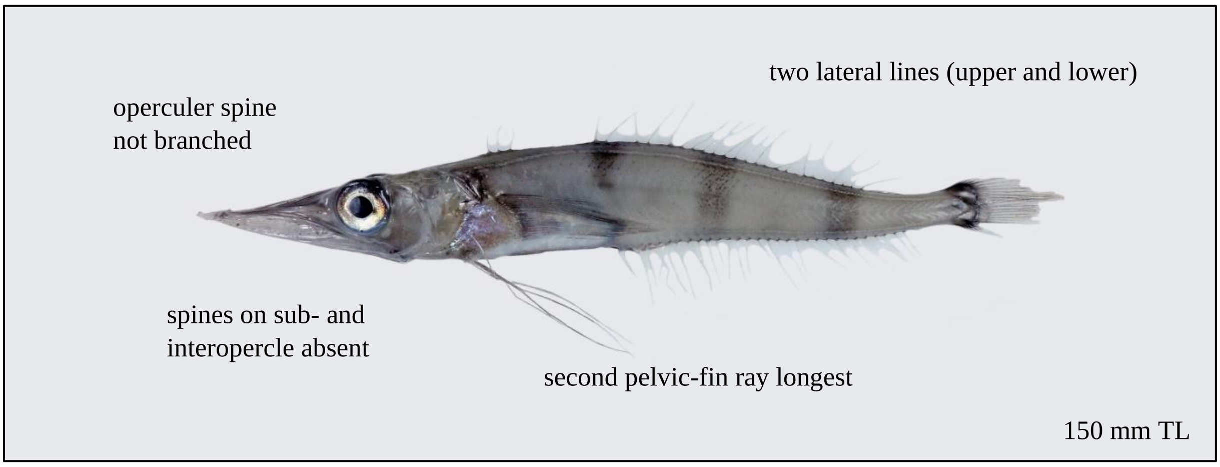

One specimen was caught at station 4005.

Channichthyidae - crocodile icefishes

Neopagetopsis ionah Nybelin, 1947 - Jonah's icefish

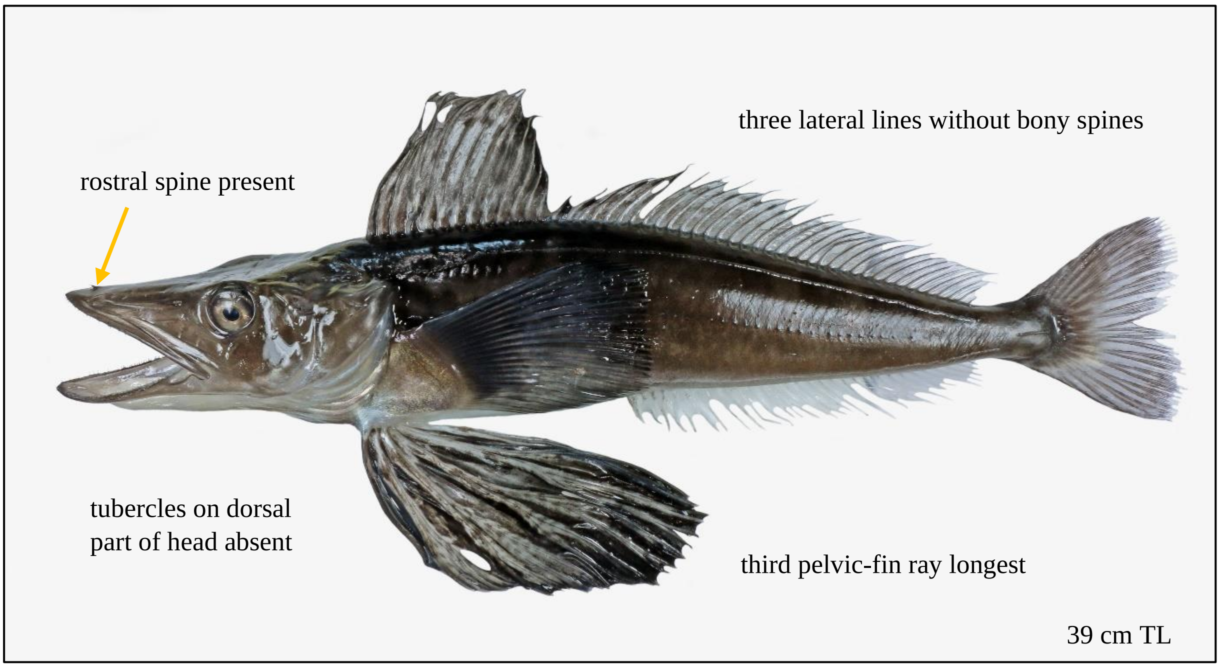

Thirteen specimens were caught, ranging in length from 18 to 48 cm TL.

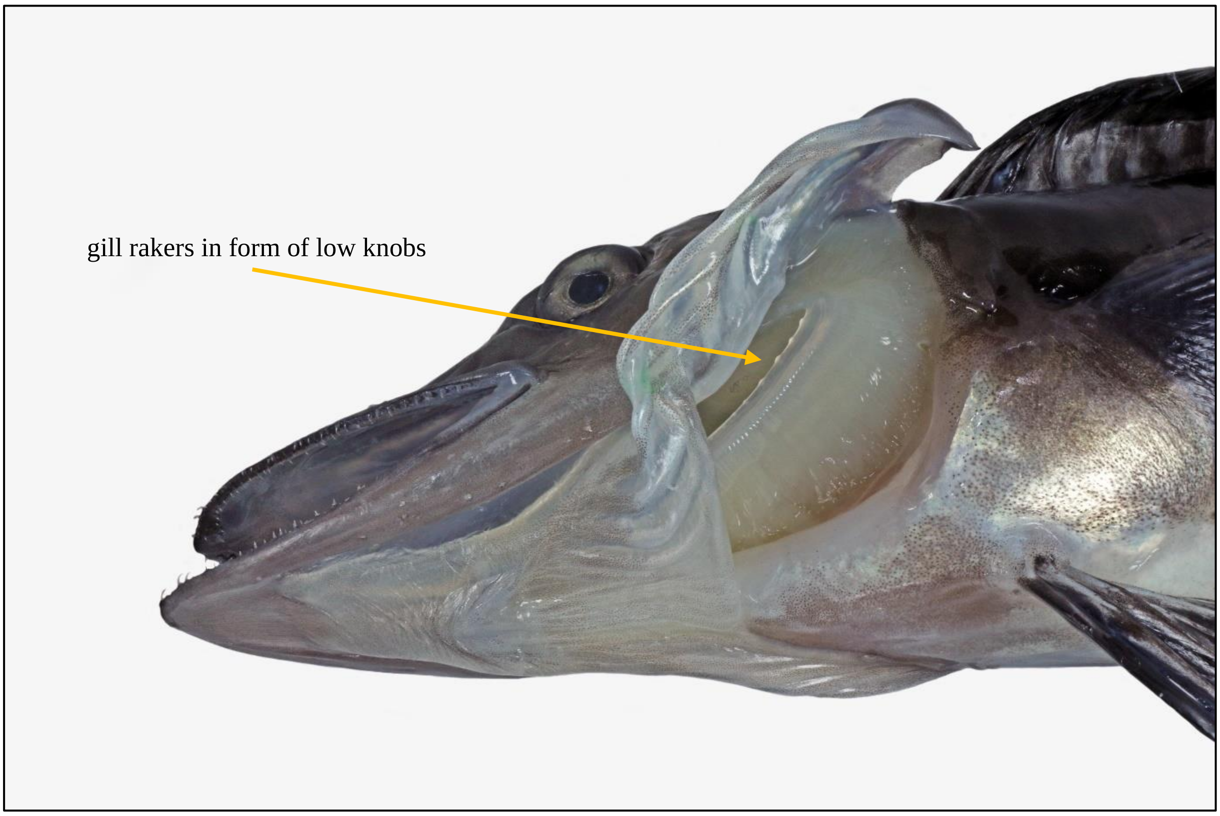

Gill filaments showing the lack of haemoglobin in crocodile icefishes (Channichthyidae).

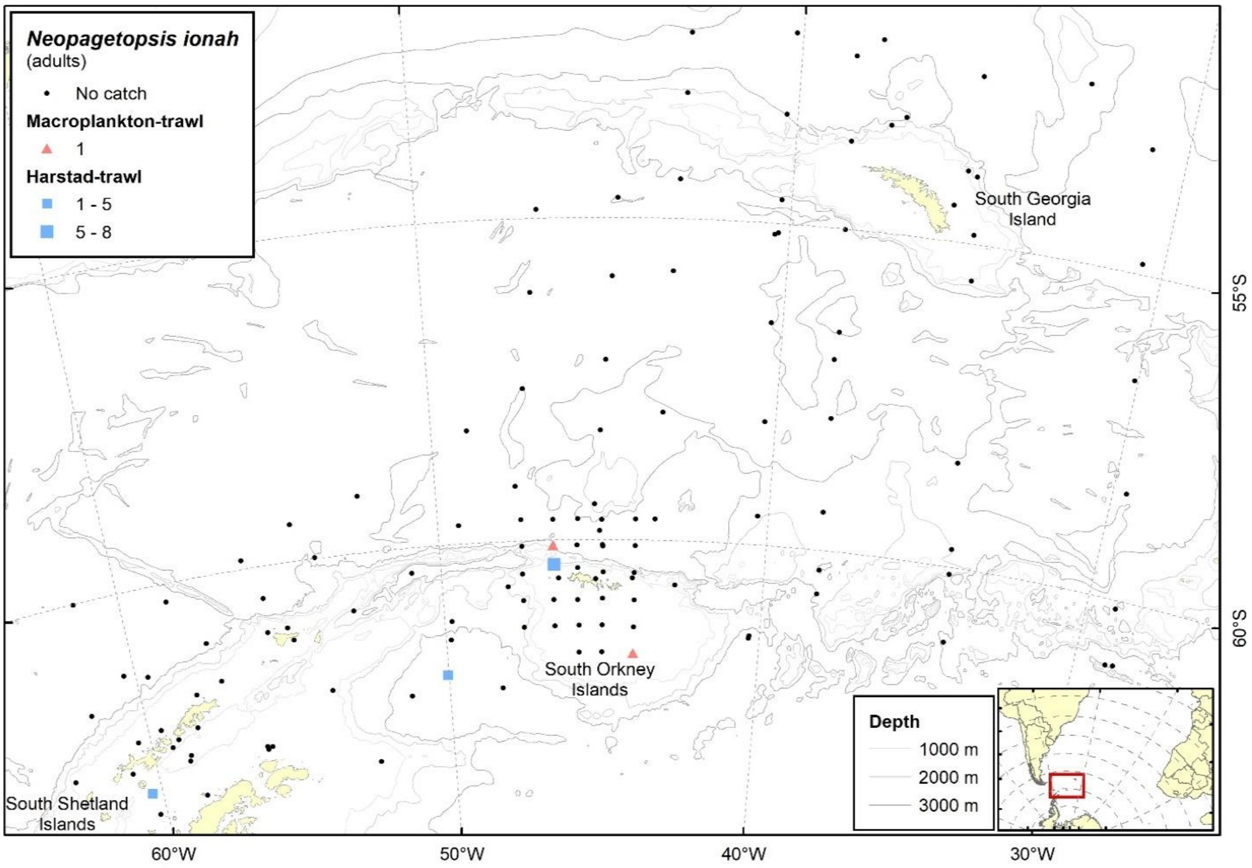

Channichthyidae - crocodile icefishes

Pagetodes antarcticus (Dollo, 1900) - long-fingered icefish

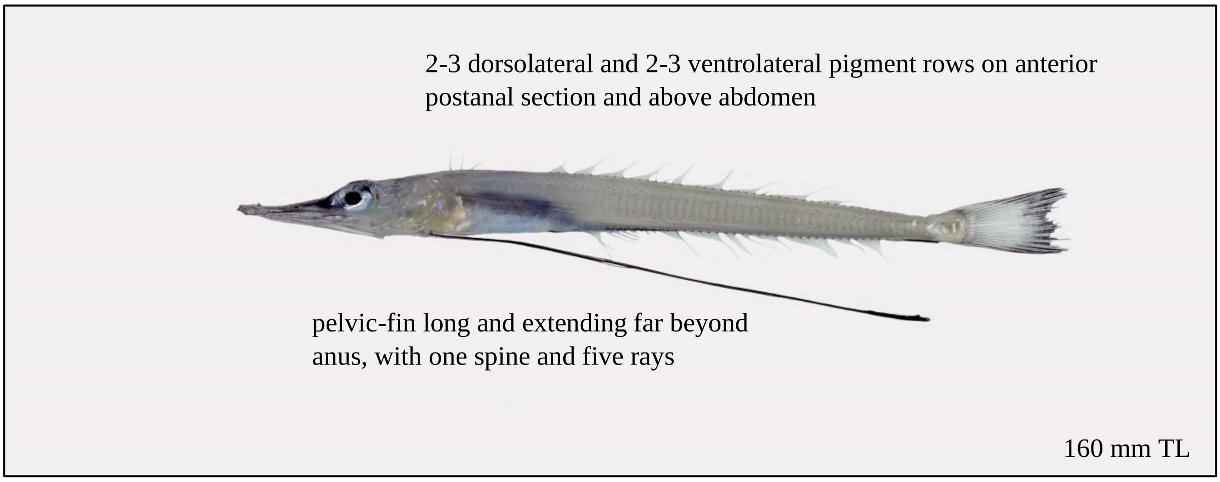

Seventeen specimens were caught, ranging in length from 63 to 160 mm TL.

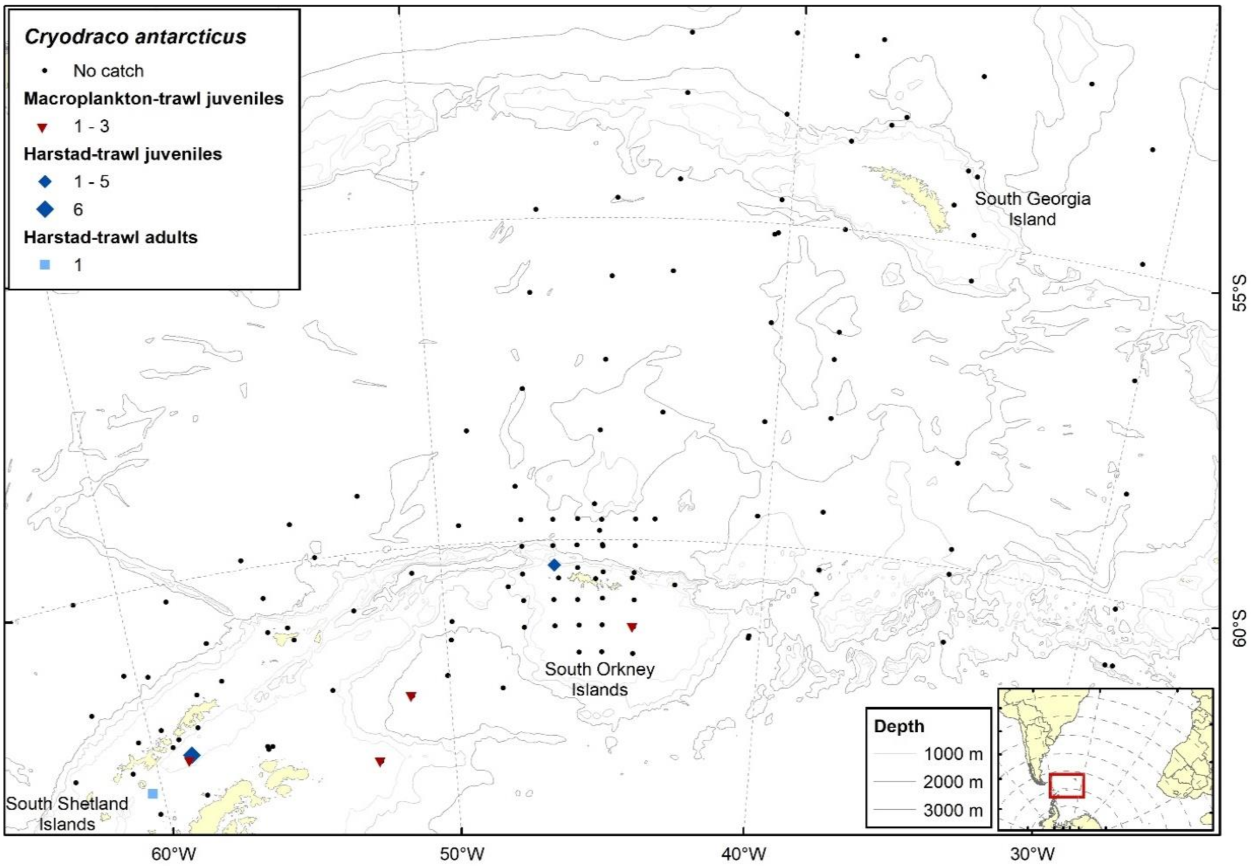

Channichthyidae - crocodile icefishes

Pseudochaenichthys georgianus Norman, 1937 - South Georgia icefish

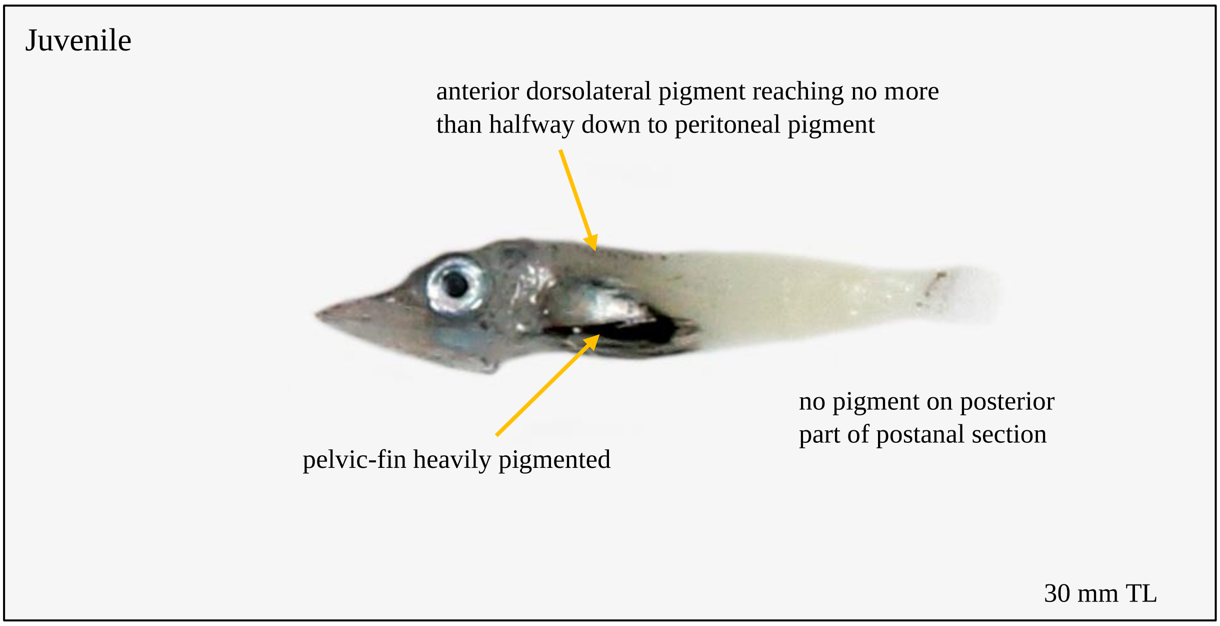

Four specimens were caught, ranging in length from 30 to 38 mm TL.

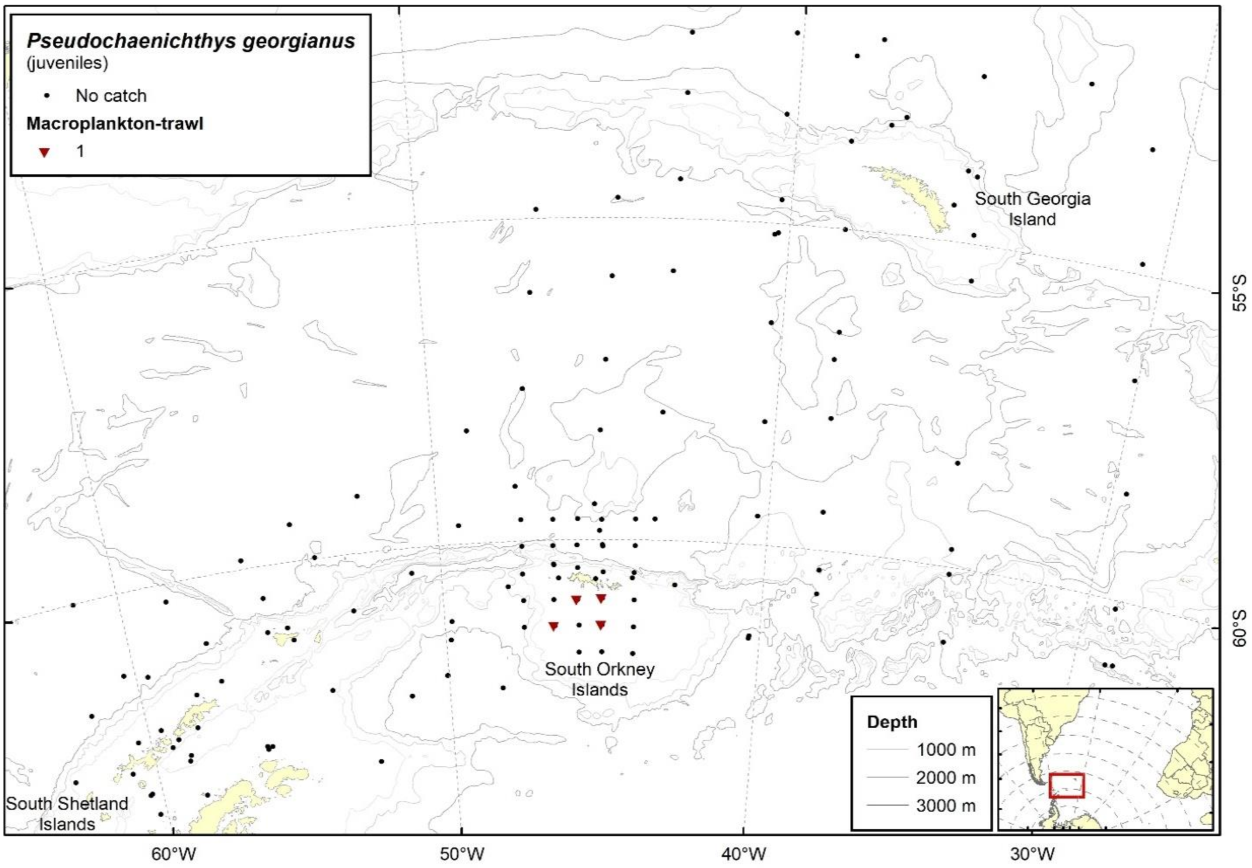
Pseudochaenichthys georgianus
(juveniles)
No catch
Macroplankton-trawl
1
South Georgia Island
South Orkney Islands
South Shetland Islands
Depth
1000 m
2000 m
3000 m
55°S
60°S
60°W
50°W
40°W
30°W

Table S1. List of stations. 4001-4067 R/V Kronprins Haakon, 4251-4318 F/V Cabo de Hornos; Depth: Max. trawling depth in m; Gear: MPT = Macroplanktontrawl, HT = Harstadtrawl; N species = number of fish species; N specimen = number of fish specimen, TW fish = total weight of fish in g, TW catch = total weight of the catch in g. Fresh weights measured onboard the vessels.

Station/ Seriesnumber	Date	Time (GMT)	Latitude	Longitude	Depth	Gear	N species	N specimen	TW fish	TW catch
4001	17.01.2019	17:03:59	-62.2826	-58.3781	244.06	MPT	1	3	0.3	5 080.0
4002	17.01.2019	22:05:16	-62.4031	-59.0856	61.44	MPT	0	0	0	40 000.0
4003	18.01.2019	10:24:23	-62.7661	-60.8217	98.38	MPT	1	1	0.4	55.2
4004	18.01.2019	16:24:46	-63.1530	-60.4168	211.25	MPT	1	1	0.3	49 658.4
4005	18.01.2019	18:18:30	-63.1371	-60.3506	682.31	HT	9	199	3 763.9	95 598.9
4006	18.01.2019	23:51:09	-63.4697	-60.2384	86.25	MPT	0	0	0	2 995.2
4007	20.01.2019	02:56:53	-63.3356	-58.5411	55.69	MPT	0	0	0	0.0
4008	20.01.2019	10:06:57	-62.7642	-58.8499	201.0	MPT	2	8	3.7	3 580.0
4009	20.01.2019	12:04:38	-62.6810	-58.7888	1001.25	HT	12	196	3 262.3	116 819.0
4010	20.01.2019	22:01:05	-62.5070	-59.3273	204.25	MPT	0	0	0	158.3
4011	22.01.2019	19:35:38	-59.6645	-47.4737	206.69	MPT	0	0	0	259.0
4012	22.01.2019	23:13:20	-60.0759	-47.4690	201.94	MPT	2	28	145.9	2 161.0
4013	23.01.2019	06:37:16	-60.5065	-47.4823	203.38	MPT	1	1	5.5	698.0
4014	23.01.2019	12:32:08	-60.9196	-47.4710	201.63	MPT	2	4	0.9	8 583.0
4015	23.01.2019	17:39:34	-61.3335	-47.4814	201.75	MPT	0	0	0	2 600.0
4016	24.01.2019	07:19:03	-61.3267	-46.5167	205.06	MPT	4	6	0.9	356.8
4017	24.01.2019	12:19:16	-60.9193	-46.5239	198.56	MPT	1	2	2.4	104.8
4018	24.01.2019	18:42:03	-60.3682	-46.5124	210.63	MPT	3	16	12.7	4 709.0
4019	24.01.2019	19:51:48	-60.3812	-46.4989	750.19	HT	12	683	7 745.7	175 486.7
4020	25.01.2019	03:58:16	-60.0794	-46.5215	203.88	MPT	5	88	813.0	4 587.0
4021	25.01.2019	10:19:32	-59.6734	-46.5033	200.81	MPT	2	4	4.2	5 025.0
4022	25.01.2019	18:18:24	-59.6670	-45.7513	214.56	MPT	0	0	0	5 032.8
4023	26.01.2019	00:16:23	-60.0747	-45.7757	210.81	MPT	6	49	438.9	9 891.0
4024	26.01.2019	05:16:50	-60.4280	-45.7678	207.88	MPT	0	0	0	320 100.0
4025	27.01.2019	20:29:18	-60.9272	-45.7739	201.5	MPT	5	5	1.2	252.3
4026	27.01.2019	23:52:00	-61.3189	-45.7429	210.19	MPT	4	19	25.1	1 011.7
4027	28.01.2019	05:35:55	-61.7392	-45.7556	209.5	MPT	4	10	15.4	1 979.9
4028	28.01.2019	13:38:37	-61.7387	-45.0177	198.94	MPT	1	5	1.7	4 658.0

4029	28.01.2019	17:12:07	-61.3195	-45.0143	211.81	MPT	6	7	1.9	1.9
4030	29.01.2019	03:37:01	-60.9062	-44.9934	184.5	MPT	5	20	205.1	420.7
4031	29.01.2019	09:32:20	-60.4990	-44.9763	214.13	MPT	2	3	27.8	15 521.9
4032	29.01.2019	15:08:46	-60.0942	-44.9838	211.63	MPT	1	2	0.3	1 565.9
4033	29.01.2019	16:41:00	-60.0717	-44.9987	1026.44	HT	16	299	5 674.1	37 779.1
4034	30.01.2019	02:28:36	-59.6826	-45.0183	201.19	MPT	8	126	1 002.4	4 494.4
4035	30.01.2019	06:47:53	-59.6737	-43.9964	210.19	MPT	6	22	196.9	4 354.9
4036	30.01.2019	10:43:02	-60.0875	-44.0000	212.31	MPT	1	1	17.0	1 932.0
4037	30.01.2019	16:20:26	-60.5069	-44.0158	204.44	MPT	1	1	0.2	373.6
4038	30.01.2019	21:24:00	-60.9278	-44.0038	208.19	MPT	8	42	15.0	3 584.9
4039	31.01.2019	01:06:16	-61.3472	-44.0154	216.13	MPT	8	27	147.5	3 112.6
4040	31.01.2019	06:26:12	-61.7586	-44.0174	206.5	MPT	4	5	432.7	5 772.0
4041	31.01.2019	20:33:14	-61.4502	-40.3319	227.25	MPT	1	11	1.0	410.0
4042	31.01.2019	21:57:29	-61.4065	-40.3216	1034.69	HT	11	301	3 030.3	79 177.3
4043	01.02.2019	16:04:44	-59.5500	-40.3191	203.19	MPT	4	16	2.4	657.8
4044	02.02.2019	03:51:42	-58.0790	-40.3033	207.19	MPT	6	37	198.5	3 007.6
4045	02.02.2019	16:37:35	-56.5395	-40.3203	206.5	MPT	3	25	1.3	708.0
4046	03.02.2019	04:00:42	-55.1415	-40.2958	214.94	MPT	8	47	363.6	8 392.0
4047	03.02.2019	07:50:04	-55.1632	-40.3914	1039.44	HT	13	428	5 579.4	41 404.1
4048	03.02.2019	15:51:45	-54.6252	-40.2613	226.31	MPT	0	0	0	225.8
4049	04.02.2019	04:02:31	-53.2898	-40.2830	235.94	MPT	11	93	359.3	3 666.2
4050	04.02.2019	16:46:48	-52.0155	-40.1618	205.0	MPT	6	27	38.6	822.2
4051	05.02.2019	02:07:14	-52.0071	-37.9938	211.75	MPT	13	118	829.9	1 650.4
4052	05.02.2019	14:18:17	-53.1726	-37.2341	249.44	MPT	3	13	3.2	705.5
4053	06.02.2019	02:09:31	-53.3180	-37.5956	240.88	MPT	9	188	896.9	4 026.2
4054	06.02.2019	15:58:52	-54.4321	-35.7343	208.25	MPT	0	0	0	40 560.0
4055	07.02.2019	02:09:00	-53.9519	-35.2357	204.94	MPT	6	36	213.1	2 587.6
4056	08.02.2019	02:12:06	-53.8752	-35.4882	199.94	MPT	7	64	327.6	929.2
4057	08.02.2019	14:10:52	-52.3894	-35.4186	217.44	MPT	3	15	5.5	694.0
4058	09.02.2019	02:09:15	-52.2359	-32.7354	210.88	MPT	6	75	361.1	2 115.8
4059	09.02.2019	14:08:54	-53.0597	-30.9225	212.94	MPT	0	0	0	304.0
4060	10.02.2019	02:04:00	-54.8292	-30.5643	210.0	MPT	9	169	1 035.5	11 682.0
4061	10.02.2019	14:06:40	-56.6194	-30.0825	208.31	MPT	1	3	0.2	395.0
4062	11.02.2019	02:05:24	-58.3571	-29.5872	208.19	MPT	4	42	296.3	2 206.6
4063	11.02.2019	14:09:03	-60.1259	-29.1044	203.94	MPT	3	4	0.3	8 299.4
4064	11.02.2019	20:58:04	-60.9909	-28.7563	213.69	MPT	2	22	2.3	3 333.5

4065	12.02.2019	01:40:53	-61.0018	-28.9980	1026.38	HT	9	473	5 438.1	23 612.1
4066	14.02.2019	21:09:54	-62.0166	-50.0245	201.19	MPT	0	0	0	738.0
4067	15.02.2019	00:27:44	-62.0149	-50.0461	1007.44	HT	12	656	9 496.4	45 916.4
4251	16.01.2019	16:11:33	-61.2051	-54.822	68.56	MPT	0	0	0	0.6
4252	17.01.2019	02:16:53	-60.4923	-55.5711	224.022	MPT	2	7	30.0	770.0
4253	17.01.2019	13:43:33	-61.0283	-55.6033	217.988	MPT	0	0	0	545.0
4254	18.01.2019	03:11:34	-61.0283	-57.5761	222.774	MPT	4	16	63.0	23 390.0
4255	18.01.2019	13:54:14	-61.7856	-58.195	221.933	MPT	0	0	0	620.0
4256	19.01.2019	01:39:44	-61.3615	-59.6127	203.259	MPT	0	0	0	5 420.0
4257	19.01.2019	14:13:14	-62.3144	-60.4018	78.343	MPT	0	0	0	1 030.0
4258	20.01.2019	01:46:00	-61.7457	-61.6754	208.0	MPT	0	0	0	34 870.0
4259	20.01.2019	15:13:00	-62.6725	-62.7516	204.0	MPT	0	0	0	2 215.0
4260	21.01.2019	06:23:00	-62.2073	-59.5783	60.0	MPT	2	2	5.1	100.0
4261	21.01.2019	15:15:00	-61.2620	-60.3584	205.0	MPT	0	0	0	1 660.0
4262	22.01.2019	02:58:00	-60.0059	-61.3593	206.0	MPT	2	17	60.0	12 300.0
4263	22.01.2019	15:09:00	-60.2781	-58.5528	206.0	MPT	1	2	10.0	12 000.0
4264	23.01.2019	02:59:42	-61.6494	-57.3246	204.0	MPT	2	3	45.0	18 040.0
4265	23.01.2019	13:12:00	-62.8140	-56.2097	172.0	MPT	3	6	1.0	23.0
4266	23.01.2019	14:25:00	-62.7691	-56.2345	45.0	MPT	0	0	0	0.0
4267	23.01.2019	15:33:00	-62.7797	-56.0656	81.0	MPT	1	1	0.5	3 345.0
4268	24.01.2019	04:25:00	-63.2465	-52.5145	203.0	MPT	6	16	5.5	2 585.0
4269	24.01.2019	15:10:00	-62.0599	-53.8163	208.0	MPT	2	2	5.0	2 290.0
4270	25.01.2019	03:32:00	-61.0011	-54.9653	210.0	MPT	3	4	48.5	13 290.0
4271	25.01.2019	15:07:00	-59.8604	-56.0624	209.0	MPT	1	1	0.1	7 495.0
4272	26.01.2019	03:03:00	-59.4259	-54.4345	200.0	MPT	3	94	660.0	8 338.0
4273	26.01.2019	18:33:00	-59.9856	-53.8157	40.0	MPT	1	3	20.0	15 340.0
4274	27.01.2019	03:07:00	-60.8770	-52.8291	210.0	MPT	3	52	566.6	4 780.0
4275	27.01.2019	15:14:45	-62.2848	-51.2465	198.0	MPT	6	11	47.2	715.0
4276	28.01.2019	15:05:00	-61.4699	-49.8415	204.0	MPT	1	2	10.0	1 235.0
4277	29.01.2019	03:04:53	-60.3907	-50.9055	211.0	MPT	2	31	280.0	32 700.0
4278	29.01.2019	15:07:00	-59.1185	-52.3247	203.0	MPT	3	4	100.0	9 235.0
4279	30.01.2019	15:13:00	-58.2493	-48.9511	214.0	MPT	0	0	0	12 411.0
4280	31.01.2019	03:02:00	-59.7099	-49.364	216.0	MPT	6	112	942.0	12 073.0
4281	31.01.2019	15:02:00	-61.1827	-49.7704	209.0	MPT	2	2	0.3	795.0
4282	01.02.2019	03:01:00	-62.2602	-48.2569	208.0	MPT	4	35	220.0	6 170.0
4283	01.02.2019	15:08:00	-60.6956	-47.9412	203.0	MPT	0	0	0	4 880.0

4284	02.02.2019	03:02:00	-59.1452	-47.6095	201.0	MPT	4	122	751.0	24 731.0
4285	02.02.2019	15:02:00	-57.6309	-47.2892	210.0	MPT	1	1	0.5	4 420.0
4286	03.02.2019	02:59:00	-56.1427	-46.9927	206.0	MPT	7	84	240.0	7 960.0
4287	03.02.2019	15:04:00	-54.8567	-46.7693	204.0	MPT	2	5	30.0	2 756.0
4288	04.02.2019	03:00:00	-54.6808	-44.5858	238.0	MPT	7	13	52.0	8 460.0
4289	04.02.2019	14:59:00	-55.8985	-44.7361	211.0	MPT	1	1	0.2	1 275.0
4290	05.02.2019	03:02:00	-57.1973	-44.907	217.0	MPT	9	56	165.0	5 850.0
4291	05.02.2019	15:03:00	-58.2935	-45.0572	233.0	MPT	3	18	5.0	695.0
4292	06.02.2019	02:58:00	-59.4400	-45.2402	225.0	MPT	5	21	83.0	2 835.0
4293	07.02.2019	05:22:00	-60.5841	-46.3691	87.0	MPT	0	0	0	151 070.0
4294	07.02.2019	15:04:00	-60.5994	-45.2076	155.0	MPT	2	2	0.3	1 280.0
4295	08.02.2019	01:30:00	-59.8500	-45.0825	208.0	MPT	6	47	428.0	10 600.0
4296	17.02.2019	19:01:00	-60.5879	-44.0652	205.0	MPT	0	0	0	140.0
4297	18.02.2019	03:08:00	-60.6808	-42.7444	211.0	MPT	2	8	7.5	665.0
4298	18.02.2019	15:04:00	-59.6649	-43.4130	210.0	MPT	2	5	1.5	20 960.0
4299	19.02.2019	02:59:00	-58.0054	-43.25	206.0	MPT	7	33	143.0	3 055.0
4300	20.02.2019	14:58:00	-55.8103	-43.0676	202.0	MPT	0	0	0	745.0
4301	21.02.2019	02:55:00	-54.3759	-42.9547	222.0	MPT	0	0	0	5 795.0
4302	21.02.2019	15:02:00	-53.0341	-42.8375	212.0	MPT	0	0	0	60.0
4303	22.02.2019	02:50:00	-52.092	-42.7687	203.0	MPT	6	14	75.0	24 120.0
4304	23.02.2019	03:01:00	-52.3019	-38.6355	218.0	MPT	11	60	1 053.0	5 698.0
4305	23.02.2019	15:06:00	-53.6279	-38.5746	225.0	MPT	1	3	1.9	410.0
4306	24.02.2019	02:56:00	-55.0042	-38.5236	231.0	MPT	5	14	126.0	2 500.0
4307	24.02.2019	15:01:00	-56.6014	-38.4130	205.0	MPT	4	7	2.5	420.0
4308	24.01.2019	19:31:00	-57.0284	-38.4832	100.0	MPT	0	0	0	32 015.0
4309	25.01.2019	02:57:00	-57.9446	-38.4099	214.0	MPT	4	16	120.0	2 740.0
4310	25.01.2019	14:57:09	-59.4005	-38.3632	204.0	MPT	3	7	2.0	1 165.0
4311	25.01.2019	22:36:00	-60.3005	-38.3083	11.0	MPT	0	0	0	2 160.0
4312	26.02.2019	03:01:00	-60.6712	-38.3031	200.0	MPT	4	76	166.0	9 425.0
4313	27.02.2019	02:55:00	-61.1584	-34.1532	200.0	MPT	3	72	186.0	21 376.0
4314	27.02.2019	21:45:00	-60.1042	-34.3202	204.0	MPT	4	12	13.0	685.0
4315	28.02.2019	02:54:00	-59.7193	-34.3760	223.0	MPT	5	33	97.0	1 980.0
4316	28.02.2019	15:01:00	-58.3860	-34.5982	200.0	MPT	2	3	0.2	5 547.0
4317	02.03.2019	14:21:43	-55.5615	-34.9923	124.0	MPT	0	0	0	5 095.0
4318	02.03.2019	23:17:00	-54.8511	-35.1108	157.0	MPT	3	37	110.0	18 150.0

Table S2. List of species and GenBank Accession Numbers. The table gives an overview of GenBank accession numbers for 27 species of Antarctic fish, and direct links to their GenBank repository. ZMUB numbers: Reference to voucher specimens at the University Museum of Bergen. – : No voucher specimen acquired.

Species	Specimen-voucher	GenBank Accession Number	Link
Bathylagus antarcticus	ZMUB 24351	PQ057120	https://www.ncbi.nlm.nih.gov/nuccore/PQ057120
Bathylagus tenuis	ZMUB 24384	PQ057121	https://www.ncbi.nlm.nih.gov/nuccore/PQ057121
Notolepis coatsorum	-	PQ057122	https://www.ncbi.nlm.nih.gov/nuccore/PQ057122
Nansenia antarctica	ZMUB 24393	PQ057123	https://www.ncbi.nlm.nih.gov/nuccore/PQ057123
Pseudoxenomystax albescens	ZMUB 24317	PQ057124	https://www.ncbi.nlm.nih.gov/nuccore/PQ057124
Muraenolepis evseenkoi	ZMUB 24330	PQ057125	https://www.ncbi.nlm.nih.gov/nuccore/PQ057125
Muraenolepis sp.	ZMUB 24332	PQ057126	https://www.ncbi.nlm.nih.gov/nuccore/PQ057126
Muraenolepis marmorata	ZMUB 24344	PQ057127	https://www.ncbi.nlm.nih.gov/nuccore/PQ057127
Electrona antarctica	-	PQ057128	https://www.ncbi.nlm.nih.gov/nuccore/PQ057128
Gymnoscopelus fraseri	ZMUB 24435	PQ057129	https://www.ncbi.nlm.nih.gov/nuccore/PQ057129
Gymnoscopelus hintonoides	ZMUB 24375	PQ057130	https://www.ncbi.nlm.nih.gov/nuccore/PQ057130
Gymnoscopelus piabilis	ZMUB 24427	PQ057131	https://www.ncbi.nlm.nih.gov/nuccore/PQ057131
Protomyctophum choriodon	ZMUB 24449	PQ057132	https://www.ncbi.nlm.nih.gov/nuccore/PQ057132
Artedidraco skottsbergi	ZMUB 24430	PQ057133	https://www.ncbi.nlm.nih.gov/nuccore/PQ057133
Pogonophryne scotti	ZMUB 24326	PQ057134	https://www.ncbi.nlm.nih.gov/nuccore/PQ057134
Prionodraco evansii	ZMUB 24431	PQ057135	https://www.ncbi.nlm.nih.gov/nuccore/PQ057135
Chaenocephalus aceratus	ZMUB 24337	PQ057136	https://www.ncbi.nlm.nih.gov/nuccore/PQ057136
Chionodraco rastrospinosus	ZMUB 24324	PQ057137	https://www.ncbi.nlm.nih.gov/nuccore/PQ057137
Cryodraco antarcticus	ZMUB 24320	PQ057138	https://www.ncbi.nlm.nih.gov/nuccore/PQ057138
Dissostichus mawsoni	ZMUB 24321	PQ057139	https://www.ncbi.nlm.nih.gov/nuccore/PQ057139
Trematomus newnesi	-	PQ057140	https://www.ncbi.nlm.nih.gov/nuccore/PQ057140
Lepidonotothen larseni	ZMUB 24335	PQ057141	https://www.ncbi.nlm.nih.gov/nuccore/PQ057141
Lepidonotothen squamifrons	ZMUB 24340	PQ057142	https://www.ncbi.nlm.nih.gov/nuccore/PQ057142
Notothenia coriiceps	ZMUB 24325	PQ057143	https://www.ncbi.nlm.nih.gov/nuccore/PQ057143
Notothenia rossii	ZMUB 24322	PQ057144	https://www.ncbi.nlm.nih.gov/nuccore/PQ057144
Pseudochaenichthys georgianus	ZMUB 24349	PQ057145	https://www.ncbi.nlm.nih.gov/nuccore/PQ057145
Pseudotrematomus eulepidotus	ZMUB 24363	PQ057146	https://www.ncbi.nlm.nih.gov/nuccore/PQ057146
